# Supplementary material for: Evaluating the feasibility and acceptability of a community dialogue intervention in the prevention and control of schistosomiasis in Nampula province, Mozambique
Source: PLoS One. 2021 Aug 5;16(8):e0255647. doi: 10.1371/journal.pone.0255647 (PMC8341517; doi:10.1371/journal.pone.0255647)

DIRECÇÃO PROVINCIAL DE SAÚDE DE NAMPULA

malaria consortium
disease control, better health

Manual do Formador

Para Treinar Facilitadores Comunitários na Condução de Diálogos sobre Bilharziose

Moçambique

Junho de 2014

Conteúdo

Notas para os Formadores ... 4
Introdução sobre o projecto de Diálogos Comunitários ... 4
Ficha Técnica do Treino ... 5
As responsabilidades do Formador ... 5
Organização do Manual ... 7
Material necessário ... 9
Agenda do Treino ... 10
Informação técnica sobre a bilharziose para o formador ... 11
Sessão 1: Introdução (2 horas) ... 16
Actividade: Abertura do Treino (10 min) ... 16
Actividade: Saudação dos participantes e Objectivos (30 min) ... 17
Actividade: Regras de convivência (10 min) ... 18
Actividade: Pre-teste (30 min) ... 19
Actividade: Apresentação sobre o Projecto (40 min) ... 20
Sessão 2: O que é Bilharziose? (1 hora e meia) ... 21
Actividade: O que sabemos sobre Bilharziose? (60 minutos) ... 21
Actividade: Informações básicas sobre Bilharziose? (30 min) ... 22
Sessão 3: O que é Diálogo? (1 hora e 20 min) ... 23
Actividade: Definição do diálogo comunitário (15 min) ... 23
Actividade: Objectivos do Diálogo Comunitário (15 min) ... 24
Cartaz: O que significa Diálogo? ... 25
Actividade: Diferença entre diálogo e sensibilização (20 min) ... 26
Cartaz: Qual é a diferença entre uma sensibilização da comunidade e um diálogo comunitário? ... 27
Actividade: Familiarização com o Guião de diálogo comunitário (20 min) ... 28
Actividade: Os 10 passos para o diálogo (30 min) ... 29
Sessão 4: Como conduzir o diálogo? (1 hora e meia) ... 30
Actividade: Apresentação do Álbum Seriado (40 min) ... 30
Actividade: Como usar o Álbum Seriado (10 min) ... 31
Actividade: Habilidades de facilitação (30 min) ... 32
Cartaz: Dicas adicionais para o facilitador do diálogo ... 33
Actividade: Temas de discussão (10 min) ... 34
Actividade: Uso da ficha de planificação e seguimento (10 min) ... 36
Sessão 5: Prática (3 horas) ... 38
Actividade: Dramatização 1 (1 hora e meia) ... 38
Cartaz: Perguntas para avaliação ... 39
Actividade: Dramatização 2 (1 hora e meia) ... 40
Sessão 6: Planificação e Monitoria (2 horas e meia) ... 41
Actividade: Primeiro Dialogo (10 min) ... 41
Exemplo de discurso de introdução durante o primeiro diálogo ... 42
Actividade: Apoio do projecto aos facilitadores (10 min) ... 44
Actividade: Ficha de Monitoria (20 min) ... 46
Actividade: Planificação (60 min) ... 47
Actividade: Encerramento do treino (60 min) ... 48
Anexos ... 50
Anexo 1: Ficha de informação sobre o projecto ... 51
Anexo 2: Ficha de monitoria ... 53

Anexo 3: Ficha de planificação e seguimento dos diálogos 55
Anexo 4: Ficha de presença – Treino em Diálogos Comunitários 56
Anexo 5: Ficha individual de PRÉ / PÓS - TESTE 59
Anexo 6: Respostas as perguntas de PRÉ / PÓS - TESTE 62
Anexo 7: Ficha de compilação de resultados individuais do pre e pós test 64
Anexo 8: Ficha de avaliação individual do treino 65
Anexo 9: Ficha de resumo da avaliação do treino 66
Anexo 10: Modelo de relatório do treino 67
Anexo 11: Modelo de certificado de participação no Treino 69

Notas para os Formadores

Introdução sobre o projecto de Diálogos Comunitários

Em Moçambique, o programa nacional de controlo das Doenças Tropicais Negligenciadas (DTN) foi criado em 2009 pelo Ministério da Saúde (MISAU). As taxas de prevalência de DTN no país são extremamente elevadas, com cerca de 2.036.633 crianças do ensino primário infectados com *schistosomose* (bilharziose) e 1.937.441 com *geohelmintos* (Augusto et al., 2009). A Província de Nampula apresenta as maiores taxas de prevalência do país para *schistosomose* (77,7%) e parasitoses (62,0%).

O programa nacional de controlo das Doenças Tropicais Negligenciadas está a implementar várias actividades, incluído campanhas integradas de administração massiva de medicamentos. Para alcançar a interrupção sustentável da transmissão das doenças, as campanhas anuais precisam manter elevadas taxas de cobertura. As campanhas de massa são por vezes prejudicadas pela fraca participação da comunidade, especialmente em áreas rurais, com alfabetização limitada. Vários factores contribuem na baixa participação em campanhas de tratamento massivo, como a compreensão limitada da sua finalidade pela comunidade e também percepções locais destas doenças por parte dos trabalhadores de saúde.

A Direcção Provincial de Saúde de Nampula, em parceria com Malaria Consortium, lançou um projecto de pesquisa operacional sobre diálogos comunitários (DC) na Província de Nampula, para apoiar a redução das doenças tropicais negligenciadas (DTNs), com financiamento da Fundação Bill & Melinda Gates e do COMDIS-HSD.

Este projecto irá adaptar e testar o modelo de diálogos comunitários no âmbito do programa de controlo da Bilharziose para aumentar a adesão ao tratamento e o conhecimento da população sobre os métodos de prevenção destas doenças, em 4 distritos da província de Nampula: Eráti, Mecuburi, Murrupula e Mogovolas.

A abordagem do Diálogo Comunitário é uma forma do processo de mobilização e capacitação da comunidade, que visa fornecer às comunidades informações, competências e confiança para ganhar o controlo sobre as decisões relacionadas com as suas próprias vidas.

A abordagem de diálogo comunitário consiste em um processo de 10 passos repetíveis, com um kit de ferramentas (guião do facilitador e cartões ilustrados), liderado por líderes da comunidade (tradicionais, religiosos ou administrativos) e activistas de saúde, que receberam um treino curto em técnicas de facilitação participativa.

A Malaria Consortium, em parceria com a Direcção Provincial de Saúde de Nampula, irá treinar um total de 200 membros de comités de saúde (50 em cada distrito) na abordagem de diálogo comunitário e uso do kit de ferramentas (guião do facilitador e álbum seriado).

Eles serão capacitados para conduzir diálogos comunitários regulares sobre a bilharziose dentro das suas comunidades para aumentar conhecimentos da população sobre medidas preventivas e de tratamento para reduzir o peso desta doença.

Ficha Técnica do Treino

Duração total: 3 dias completos com intervalos

Participantes: Facilitadores comunitários seleccionados por cada Comité de Saúde.

Objectivos de aprendizagem:

No fim do treino, os facilitadores estarão:

- Familiarizados com principais sinais e sintomas, causas, e medidas preventivas e de tratamento da bilharziose,
- Familiarizados com os objectivos, processo e o Kit de DC,
- Capazes de liderar sessões regulares de DC nas suas respectivas comunidades,
- Capazes de desenvolver um plano de acção para a implementação de DCs regulares.

As responsabilidades do Formador

➔ Planificar a formação nos vários distritos identificados.

➔ Oferecer a formação:

  - ***em língua local*** usando este Manual;
  - Aplicar uma variedade de técnicas eficazes de formação participativa para aumentar a retenção e aplicação do conteúdo da formação.
  - Dar aos participantes retro-informação construtiva para melhorar seu desempenho.

➔ Avaliar o impacto e eficácia da formação e fazer relatório

Formar adultos é mais do que dar-lhes informações; é preciso assegurar que os adultos podem aplicar o que aprenderam de modo a produzir resultados positivos para a saúde da comunidade. Isso é feito permitindo que os participantes partilhem as suas experiências, façam perguntas, e processem a informação, a fim de absorver o conteúdo fundamental.

➢ Criar um ambiente de aprendizagem onde os participantes se sentem confortáveis para trocar e partilhar ideias, e fazer perguntas livremente.

- Envolver todos os participantes e incentivar a participação.
- Dar instruções de forma clara.
- Facilitar discussões em grupo, demonstrações, prática e dramatização.
- Ajudar os participantes a identificar como aplicar as habilidades aprendidas no treino para o seu trabalho na comunidade.

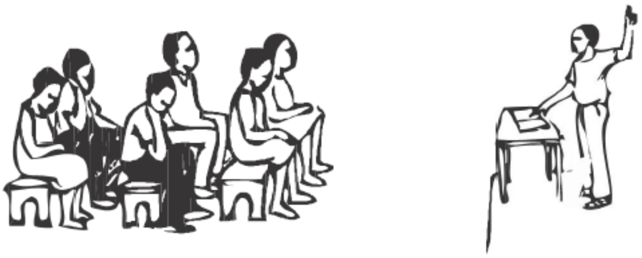	Não falar para as pessoas
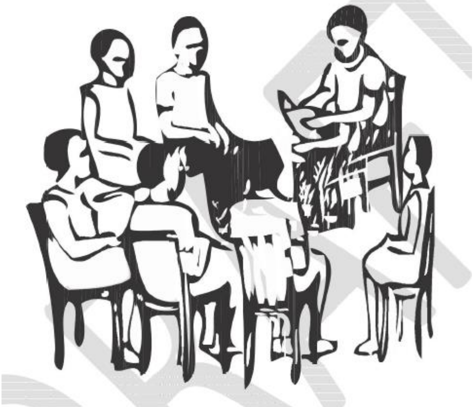	Falar com as pessoas

Organização do Manual

O presente manual está estruturado em 6 **sessões**:

- Sessão 1: Introdução
- Sessão 2: O que é Bilharziose?
- Sessão 3: O que é Diálogo?
- Sessão 4: Como conduzir diálogo?
- Sessão 5: Prática
- Sessão 6: Planificação e Monitoria

Cada sessão tem um conjunto de actividades. Cada actividade será feita seguindo uma série de passos. A ficha de cada actividade tem:

- ✓ Um Título
- ✓ Um Objectivo específico
- ✓ Uma Duração indicativa
- ✓ Uma descrição dos Passos: os passos para realizar a actividade são ilustrados com um símbolo do método.
- ✓ Cada sessão acaba com um sumário das mensagens chave.

Cada passo para realizar a actividade é ilustrado com um símbolo, como se segue:

**Actividade**	Título da actividade
**Objectivos**	Objectivos específicos de aprendizagem
**Duração**	Duração aproximada em minutos
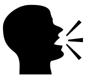	**Apresentação**<br>O formador vai mostrar ou explicar um assunto.
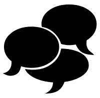	**Discussão em plenário**<br>Chuva de ideias ou perguntas e respostas com todo o grupo de participantes.
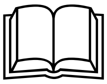	**Leitura**<br>Leitura individual ou entre um grupo pequeno
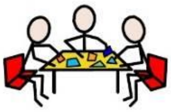	**Prática**<br>Instruções para a dramatização, exercício, ou jogo.
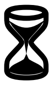	**Tempo necessário**<br>O formador dá tempo aos participantes para pensar, ler ou escrever.
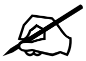	**Notas**<br>O Formador escreve as respostas ou sugestões dos participantes no papel gigante.
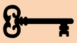	**Mensagem chave**<br>O Formador deve encerrar cada sessão com um sumário das mensagens chave no fim da actividade.
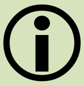	**Dicas**<br>Informações adicionais úteis para o formador executar ou preparar a actividade.

Material necessário

Item	Número
Ficha de presença	3 cópias (1 cópia por cada dia) por treino
Papel gigante e Marcadores	1 pacote por treino
Blocos de notas e canetas	1 para cada participante
Pasta de Plástico	1 para cada participante
Certificado	1 para cada participante
Camisetas	1 para cada participante
Guião de diálogo comunitário	1 para cada participante
Álbum Seriado sobre Bilharziose	1 para cada participante
Ficha de monitoria de diálogo comunitário	1 para cada participante
Ficha de planificação	1 para cada participante
Ficha individual de avaliação do treino	1 cópia para cada participante
Ficha individual de pré e pós teste	2 cópias para cada participante

Agenda do Treino

**Horário**	**DIA 1**	**DIA 2**	**DIA 3**
07:30 – 08:00	Inscrição dos participantes. Logística	Síntese do dia anterior	Síntese do dia anterior
	**Sessão 1: Introdução**	**Sessão 4: Como conduzir o diálogo?**	
08:00 – 09:00	Actividade: Abertura do Treino<br>Actividade: Saudação e Objectivos	Actividade: Álbum seriado<br>Actividade: Uso do Álbum	Actividade: Dramatização 2
09:00 – 10:30	Actividade: Regras de convivência<br>Actividade: Pré-teste<br>Actividade: Projecto	Actividade: Dicas de facilitação	
**10:30**	**Lanche**	**Lanche**	**Lanche**
	**Sessão 2: O que é Bilharziose?**	Actividade: Temas de discussão	**Sessão 6: Planificação**
11:00 – 12:00	Actividade: O que já sabemos?	Actividade: Ficha de planificação	Actividade: Apoio do projecto
12:00 – 12:30	Actividade: Informações Bilharziose		Actividade: Ficha de monitoria
**12:30 – 13:30**	**Almoço**	**Almoço**	**Almoço**
	**Sessão 3: O que é diálogo?**	**Sessão 5: Pratica**	
14:00 – 15:00	Actividade: Definição<br>Actividade: Objectivos<br>Actividade: Diferença com palestra	Actividade: Dramatização 1	Actividade: Planificação
15:00 – 16:00	Actividade: O guião de diálogo<br>Actividade: Os 10 passos	Actividade: Dramatização 1	Actividade: Encerramento
16:00 -16:30	Avaliação do dia	Avaliação do dia	

Informação técnica sobre a bilharziose para o formador

Definição

Bilharziose (Schistosomíase) é uma parasitose helmíntica, geralmente com carácter crónico que infecta os seres humanos, causada pelo trematódo do género *Schistossoma.* É uma doença muito antiga, tendo sido encontrado ovos desses helmintos em múmias egípcias de pessoas que viveram em torno de 3.500 antes de Cristo.

Epidemiologia

A Bilharziose (Schistosomíase) é um problema de saúde pública que afecta cerca de 240 milhões de pessoas em todo mundo, principalmente nas áreas tropicais e subtropicais. Em Moçambique, a prevalência da Schistosomíase é bastante elevada, sendo a mais grave a forma urinária (que afecta a bexiga) do que a forma intestinal.

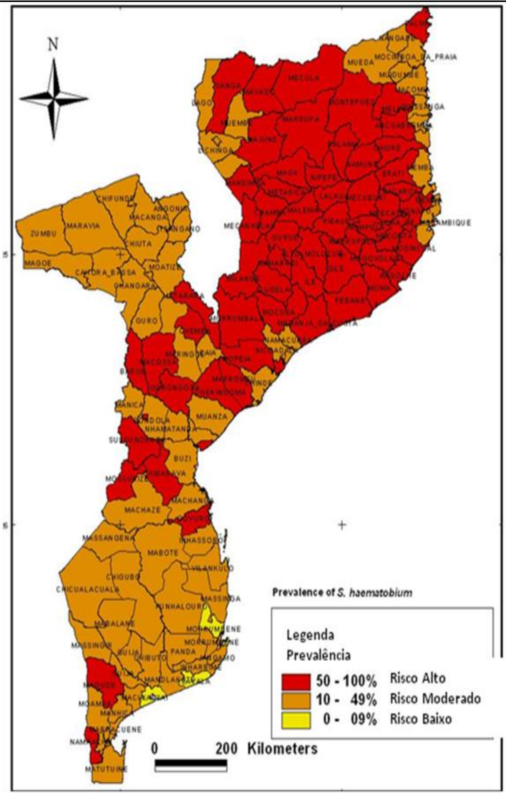

Fonte: Ministério de Saúde, Instituto Nacional de Saúde

Figura 1. Prevalência da Forma Urinária da Schistosomíase (*Schistosoma haematobium*) em Moçambique

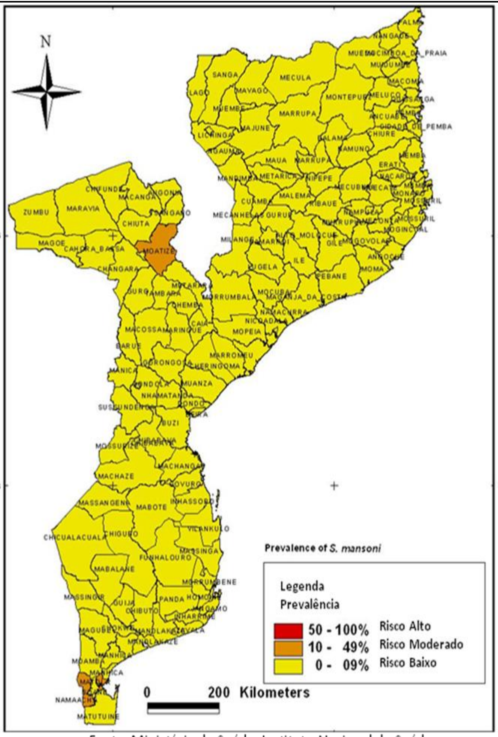

Fonte: Ministério de Saúde, Instituto Nacional de Saúde

Figura 2. Prevalência da Forma Intestinal da Schistosomíase (*Schistosoma mansoni*) em Moçambique

Causa

A causa da Bilharziose (Schistosomíase) é o trematode do género *Schistosoma*. O *S. haematobium e o S. mansoni* são os únicos agentes etiológicos em Moçambique.

O *Schistosoma haematonium* é o agente etiológico da forma urinária. O *Schistosoma mansoni, S. japonicum, S. mekongi e S. intercalatum* são os agentes etiológicos da forma intestinal.

Transmissão e Ciclo de Vida

A transmissão é efectuada de forma indirecta através do caracol de água doce. O ciclo de vida da Schistosomíase ocorre entre o caracol e o ser humano.

Ciclo de Vida no Ser Humano

A infecção começa quando pequenos bichinhos (chamadas de cercárias) libertadas pelo caracol penetram na pele do ser humano. Na pele ficam durante 2 – 4 dias, onde se transformam em esquistossômulos. Os esquistossômulos migram através das veias e vasos linfáticos, passando pelos pulmões, coração e fígado e desenvolvem-se transformando-se em vermes. Os vermes sexualmente maduros migram para locais específicos onde acasalam sexualmente. Após acasalamento, as fêmeas grávidas "viajam" através do sangue para depositar os ovos. Metade dos ovos tem acesso ao intestino ou bexiga (dependendo da espécie) e são eliminados. A outra metade fica retida no corpo e através do sangue vão para o fígado e outras partes do corpo.

Ciclo de Vida no Caracol

Após eliminação dos ovos (pelas fezes – *S. mansoni* e pela urina – *S. haematobium*), os mesmos alcançam colecções de água doce, eclodem e libertam bichinhos (chamados de miracídeos) livres que penetram nos caracóis onde se multiplicam e depois de 4 a 6 semanas são eliminados pelo caracol na forma de pequenos bichinhos (cercárias), reiniciando o ciclo.

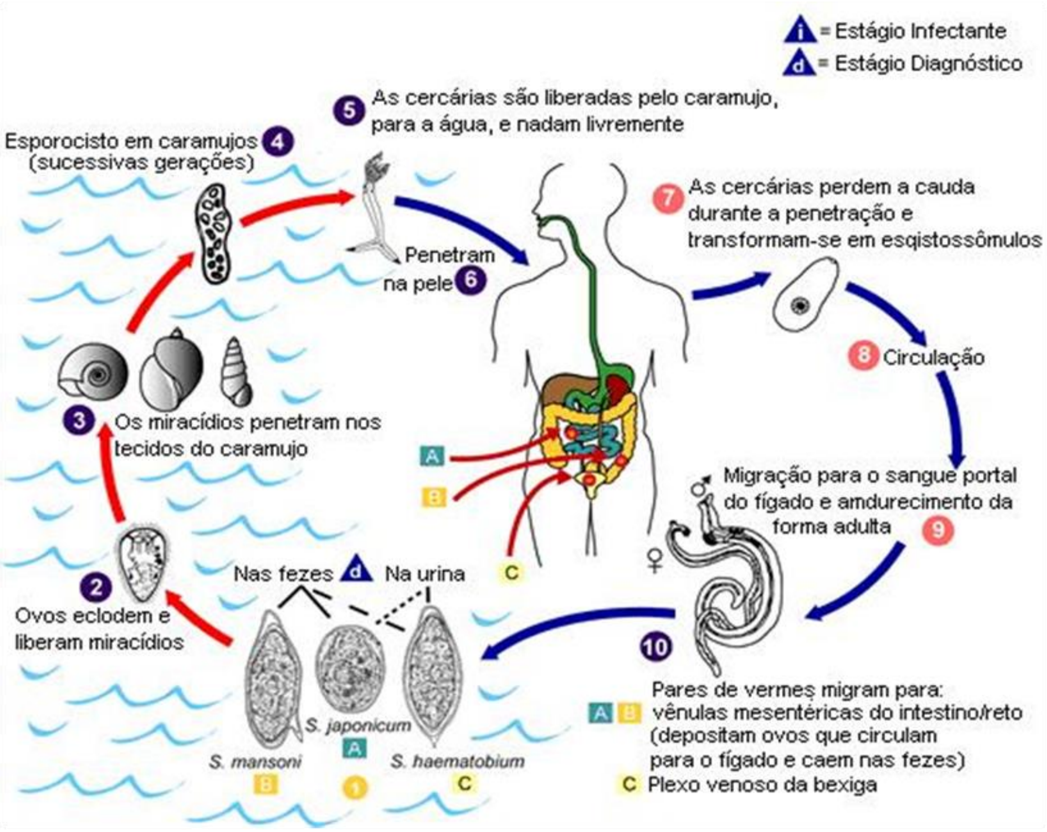

Fonte: CDC
www.dpd.cdc.gov

Figura. Ciclo de Vida do Schistosoma

Factores de Risco

Os factores de risco para a doença são determinados pela baixa condição socioeconómica, com baixa disponibilidade de água potável e precárias condições de sanidade. Hábitos de fecalismo e urinar a céu aberto são factores de risco para a infecção. A presença de caracóis de água doce em lagoas e rios, em que as pessoas vão realizar as suas actividades (tomar banho, pescar, natação de lazer, lavar a roupa, ou carregar a água para consumo) desempenha um papel importante na transmissão da infecção. Os pescadores e agricultores representam um grupo de risco importante.

Manifestações, tratamento, prevenção

A maior parte das pessoas infectadas são assintomáticas (NÃO TEM MANIFESTAÇÕES DA DOENÇA).

Manifestações da Bilharziose

Podemos dividir as manifestações da bilharziose em 3 fases:

- Dermatite cercária (após penetração das cercárias – pequenos bichinhos) – bilharziose no início
    - Também chamada de comichão do nadador

    - O doente fica com comichão e pele vermelha
    - Geralmente a comichão passa por si só e dura cerca de 5 dias
- Bilharziose (Schistosomíase) aguda: Caracterizada por febre, mal-estar, dores musculares, comichão com pele vermelha, diarreia (por vezes com sangue), tosse seca (sem escarro).
- Bilharziose (Schistosomíase) crónica.
    - Dor ao urinar, **com sangue no fim da urina** e a pessoa urina muitas vezes por dia mas em pequenas quantidades;
    - **Diarreia (pode ter sangue)**, podendo alternar com prisão de ventre (obstipação – não evacuar durante muito tempo)
    - Cansaço, incapacidade de realizar actividades rotineiras é frequente (devido a anemia)
    - **A barriga pode ficar inchada (barriga de água)**; podem surgir "bolas" dentro da barriga ou "caroços"

Diagnóstico

O diagnóstico é fundamentalmente clínico (através das manifestações que o doente apresenta) e confirmado pelo exame de urina ou fezes.

Doenças semelhantes incluem: Malária; Tuberculose; Cancros; Infecção urinária; Outras doenças

Tratamento

O tratamento da Bilharziose (Schistosomíase) é dividido em tratamento médico (com medicamentos) e cirúrgico (operação).

Tratamento Médico (com medicamentos): Praziquantel – em dose única.

Tratamento Cirúrgico (operação): Opera-se quando a doença está numa fase avançada, por exemplo quando apresenta barriga de água, ou quando a pessoa começa a vomitar sangue

Prevenção

- Tratamento 2 vezes por ano de toda população, nas zonas infestadas (tratamento massivo); Os facilitadores devem saber que o tratamento é para todos porque as autoridades sanitárias presumem que grande parte das pessoas têm a doença, apesar de não apresentarem sinais e sintomas. Assim através das campanhas, está-se a curar várias pessoas já com bilharziose;
- Tratamento das pessoas adoecidas;
- Informar a população das medidas preventivas da doença;
- Saneamento de meio (latrinas);
- Uso de Água tratada ou fervida;
- Evitar entrar em contacto com água parada infectada (presença de caracóis);

- Protecção dos pés e pernas com botas de borracha.

Sessão 1: Introdução (2 horas)

Actividade: Abertura do Treino (10 min)

**Actividade**	Abertura do treino
**Objectivos**	• Explicar o peso da bilharziose e a importância de prevenir e tratar através de acções simples que as pessoas podem tomar ao nível individual e das comunidades<br>• Explicar a importância para eles em participar neste treino e o que eles têm a ganhar com a aprendizagem
**Duração**	10 Minutos
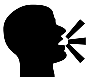	O formador deseja boas vindas aos participantes e explica qual é o propósito do treino usando o Guião de diálogo comunitário, com foco sobre:<br>• Porque falar da bilharziose?<br>• Porque fazer diálogos comunitários sobre a bilharziose?<br><br>**Objectivo do treino**<br>Esta formação visa capacitar cada pessoa que foi escolhida pela sua própria comunidade para ser capaz de conduzir diálogos comunitários regulares, dentro da sua aldeia ou bairro, para aumentar os conhecimentos das pessoas e reduzir o peso da doença.
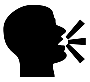	O formador o (s) convidado (s) é convidado (s) a acolher e abrir a formação oficialmente.

Actividade: Saudação dos participantes e Objectivos (30 min)

**Actividade**	Saudação dos participantes e Objectivos
**Objectivos**	Os participantes são capazes de identificar o outro pelo seu nome<br>Os participantes estão claros sobre os objectivos do treino
**Duração**	30 minutos
	O formador pede aos participantes para trabalharem aos pares
	Tempo: 3-5 minutos para os participantes colherem informação básica um do outro acerca de:<br>• Nome preferido; De onde vem<br>• Outra coisa interessante (alimentação favorita, cores, o que gosta de fazer, etc.)<br>• Expectativas a volta da formação
	**Discussão em plenário**<br>Aos pares, os participantes apresentam um ao outro, incluindo suas expectativas.
	O Formador escreve as expectativas dos participantes no papel gigante.
	**Apresentação**<br>O Formador apresenta os objectivos da formação<br>✓ Os participantes estão familiarizados com a Bilharziose e as mensagens chave para prevenção e tratamento<br>✓ Os participantes compreendem os objectivos e o processo de diálogo comunitário<br>✓ Os participantes são capazes de liderar diálogos comunitários regulares e com sucesso na sua comunidade<br><br>O Formador apresenta a Agenda do treino.
	O Formador deve preparar um cartaz (papel gigante) com os objectivos, e um outro com o Agenda do treino, antes do treino. Os cartazes devem ser pendurados na sala durante todo o dia do treino.
	O Formador pergunta se os participantes concordam com os objectivos ou se existem outras sugestões.

Actividade: Regras de convivência (10 min)

**Actividade**	Regras de convivência
**Objectivos**	Os participantes concordam sobre as regras.
**Duração**	10 Minutos
	O formador explica que as regras básicas de convivência são usadas para ajudar o grupo a trabalhar e aprender junto como uma equipe; em seguida, o formador apresenta as regras básicas:<br>✓ Respeitar as opiniões dos outros<br>✓ Falar uma pessoa de cada vez<br>✓ Fazer perguntas e participar activamente<br>✓ Participar em todas actividades<br>✓ Cumprir com as horas de início, pausa, lanche, almoço<br>✓ Assistir a todas as sessões do princípio ao fim<br>✓ Telefones celulares desligados (se houver)<br>✓ Evitar saídas desnecessárias das aulas.
	O Formador deve ter as regras básicas escritas num Cartaz (Papel gigante) antes do treino começar. O Cartaz deve ser afixado na sala durante todo treino.
	**Discussão em plenário**<br>O Formador pergunta aos participantes se concordam com as regras ou se existem outras a acrescentar.
	O Formador escreve as regras adicionais no papel gigante/cartaz.

Actividade: Pre-teste (30 min)

**Actividade**	Pré-teste
**Objectivos**	Testar os conhecimentos dos participantes com o propósito de:<br>• Avaliar as necessidades específicas de aprendizagem;<br>• Adequar a formação às necessidades reais
**Duração**	30 Minutos
	O formador distribui as fichas de pré-teste e explica que cada participante deverá marcar um círculo na sua resposta (ou nas suas respostas): Algumas perguntas têm só uma resposta correcta, outras perguntas têm várias repostas correctas e todas devem ser marcadas com círculo.
	Dar 20 minutos aos participantes para preencher<br>E aconselhável o Formador ler em voz alta cada pergunta, para permitir aos participantes com baixa capacidades de leitura de fazer o teste.
	O Formador deve compilar e analisar os resultados destes testes no final do primeiro dia da formação, a fim de ser capaz de adaptar o conteúdo da formação às necessidades de aprendizagem dos participantes.

Actividade: Apresentação sobre o Projecto (40 min)

**Actividade**	**Apresentação breve sobre o Projecto Diálogos comunitários para o controlo e prevenção das DTNs em Moçambique**
**Objectivos**	Os Participantes estão familiarizados com o projecto de diálogos comunitários para o controlo da bilharziose
**Duração**	40 Minutos
	O Formador pergunta se os participantes neste treino já participaram numa reunião de informação da comunidade sobre este projecto, antes de chegar ao treino.<br>O Formador deixa os participantes partilhar as suas ideias sobre:<br>✓ Que projecto é esse?<br>✓ O que pretende o projecto?<br>✓ O que são diálogos comunitários?<br>✓ O que são doenças tropicais negligenciadas?<br>✓ Quais distritos vão ser abrangidos?<br>✓ Quais actividades vão acontecer?
	O Formador usa a Ficha sobre o Projecto para esclarecer dúvidas dos participantes e assegurar que todos estão ao mesmo nível de informação.
	Distribuir aos participantes o guião de diálogo comunitário; a Ficha de Informação sobre o projecto está na primeira parte do guião, mas NÃO distribuir o álbum seriado
	O Formador explica as responsabilidades dos facilitadores comunitários no âmbito deste projecto, após o treino.
	**Mensagem chave**<br>✓ *Os **facilitadores** são responsáveis (depois do treino) por conduzir diálogos regulares nas suas próprias comunidades e preencher um resumo de cada diálogo na ficha de monitoria.*<br>✓ Os ***facilitadores*** irão receber um subsídio de comunicação (crédito) a ser usado para reportar questões técnicas e desafios logísticos urgentes aos supervisores a nível da província (ponto focal DTNs, DPS).<br>✓ *Os **Supervisores** (distrito, DPS e Malaria Consortium) são responsáveis para responder a perguntas técnicas ou ajudar a resolver dificuldades;*<br>✓ *Os supervisores não vão visitar cada um dos facilitadores para visita de observação. Só algumas comunidades serão observadas; os supervisores irão informar com antecedência da visita.*<br>✓ *Reuniões de balanço: trazer registo dos diálogos realizados; compartilhar experiência; resolver problemas juntos.*

Sessão 2: O que é Bilharziose? (1 hora e meia)

Actividade: O que sabemos sobre Bilharziose? (60 minutos)

**Actividade**	O que sabemos sobre bilharziose?
**Objectivos**	Explorar percepções e conhecimentos dos participantes sobre bilharziose<br>Demonstrar habilidades de facilitação de diálogo
**Duração**	60 Minutos
	Um diálogo comunitário acontece quando os membros da comunidade se reúnem para discutir questões do seu interesse. Este Diálogo Comunitário já está a acontecer na maioria das comunidades para discutir vários temas durante as reuniões da comunidade.<br>O Formador pede aos participantes de se sentar num círculo, duma maneira similar a uma reunião comunitária, e explica: “ Agora, eu vou liderar um diálogo para a nossa comunidade; eu vou jogar o papel do facilitador, e vocês dos participantes no diálogo”.
	O Facilitador deve usar os guiões de discussão do Guião de Dialogo Comunitário e liderar um diálogo como se fosse ao nível da comunidade, usando o Álbum Seriado
	Depois, o Formador estimula um debate:<br>Esta formação é sobre como organizar e conduzir diálogos dentro da sua comunidade para reduzir a bilharziose. Estimular o debate:<br>• O que acharam deste diálogo?<br>• O que gostaram e não gostaram?<br>• Acham que será difícil de conduzir um diálogo na vossa comunidade? Porquê? Que dificuldades podem surgir?
	Esta formação é sobre como organizar e conduzir diálogos dentro da sua comunidade para reduzir a bilharziose. Os objectivos do diálogo são:<br>• **Partilhar conhecimento sobre como reduzir** a bilharziose e promover uma comunidade saudável;<br>• **Identificar acções** factíveis para tratar e prevenir a bilharziose nas famílias e nas comunidades.<br>• **Concordar e implementar juntos** acções individuais e colectivas para efectivamente reduzir a doença.

Actividade: Informações básicas sobre Bilharziose? (30 min)

**Actividade**	Informações básicas sobre Bilharziose?
**Objectivos**	Ter domínio de factos básicos sobre a bilharziose<br>Responder as questões técnicas sobre a doença
**Duração**	30 Minutos
	O Formador apresenta factos sobre a doença, usando **"Informações básicas sobre a bilharziose" do Guião de Dialogo Comunitário**:<br>✓ Como se manifesta a bilharziose?<br>✓ Como se trata a bilharziose?<br>✓ Como se apanha a doença?<br>✓ Como se trata?<br>✓ Como se previne?
	Perguntas e Respostas
	O Facilitador poderia também usar as usar a ficha de "Informação técnica sobre a bilharziose para o formador", no *início* deste manual para responder às perguntas dos participantes.
	A Bilharziose (Schistosomíase) é uma doença, geralmente crónica, em Moçambique, que afecta todos os membros da comunidade que usam a água dos rios, lagoas ou charcos. A doença é causada por bichos, que vivem em caracóis na água.<br><br>A bilharziose pode aparecer de várias formas, com dor ao urinar, com sangue na urina, diarreia (que pode ter sangue) e alternar com prisão de ventre. Nas fases mais avançadas pode haver barriga de água.<br><br>O praziquantel é o medicamento para tratar a bilharziose.<br><br>As medidas de higiene individual e colectiva, saneamento do meio e o tratamento de toda a população com praziquantel são medidas de prevenção da bilharziose.

Sessão 3: O que é Diálogo? (1 hora e 20 min)

Actividade: Definição do diálogo comunitário (15 min)

**Actividade**	Definição do diálogo comunitário
**Objectivos**	Participantes concordam sobre a Definição do diálogo comunitário
**Duração**	15 Minutos
	**Discussão em plenário**<br><br>Chuva de ideias: O Formador pergunta aos participantes:<br><br>• *Na vossa opinião, o que é diálogo comunitário?*<br>• *O diálogo já ocorre nas vossas comunidades?*<br>• *Quais são os benefícios das comunidades dialogarem sobre questões de saúde?*
	**Notas**<br>O Formador escreve as respostas no papel gigante.
	"O diálogo comunitário" é diferente da "sensibilização da comunidade" ou palestra. Numa palestra, usualmente, uma pessoa fala para a comunidade sobre um tema. Num diálogo, todos os participantes podem partilhar informação e experiências sobre o tema. Durante o Diálogo, os participantes têm que discutir em conjunto o que a comunidade deveria fazer para resolver os problemas de saúde e assim melhorar a situação. No final do diálogo, os membros da comunidade devem chegar a acordo sobre acções a serem tomadas e como essas acções serão implementadas.
	**Mensagem chave**<br>✓ *Um diálogo é uma conversa com a comunidade que pode assumir muitas formas, tais como reuniões regulares ou extraordinárias ao nível da comunidade. Diálogo comunitário é quando os membros da comunidade se reúnem para discutir questões de interesse para a comunidade.*

Actividade: Objectivos do Diálogo Comunitário (15 min)

**Actividade**	Objectivos do Diálogo Comunitário
**Objectivos**	Participantes concordam sobre os objectivos do diálogo comunitário
**Duração**	15 Minutos
	**Apresentação**<br>O Facilitador apresenta os 3 objectivos, e explica que precisamos de:<br>○ *Informar as pessoas sobre os sintomas da doença; Aumentar a conscientização dos membros da comunidade sobre os riscos de não prevenir ou tratar a bilharziose*<br>○ *Explicar como prevenir a como tratar a doença*<br>○ *Mobilizar as comunidades para prevenir a doença na comunidade.*
	Em seguida, o facilitador apresenta e explica quais são os três passos importantes do diálogo comunitário, usando o cartaz:<br>• Explorar: *informações*<br>• Identificar acções: *Como esta informação pode ser aplicada em nosso quotidiano pessoal e como esta acção pode melhorar as nossas vidas?*<br>• Tomar decisões: *o que vamos fazer para melhorar a situação?*
	Antes do treino, o formador deve reproduzir o Cartaz fornecido abaixo em papéis gigantes utilizando marcadores coloridos.
	**Discussão em plenário**<br>Chuva de ideias ou perguntas e respostas com todo o grupo de participantes.
	**Mensagem chave**<br>✓ *Os três passos importantes do diálogo comunitário são:*<br>❒ *Explorar:* para dar todos os participantes o mesmo nível de informação<br>❒ *Identificar acções:* para identificar o que as pessoas estão a fazer para prevenir e tratar a doença, e identificar o que eles poderiam fazer diferente ou mais para melhor resultados.<br>❒ *Tomar decisões:* para trabalhar em conjunto para resolver problemas, incluindo quem vai fazer o quê? Quando? Como?

Cartaz: O que significa Diálogo?

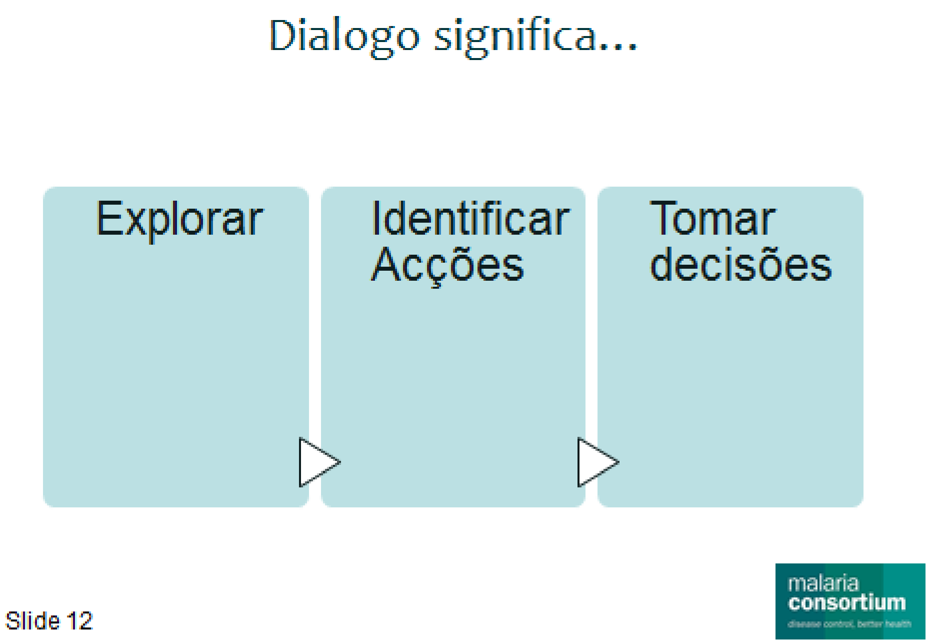

Actividade: Diferença entre diálogo e sensibilização (20 min)

**Actividade**	Diferença entre diálogo comunitário e sensibilização
**Objectivos**	Participantes conhecem a Diferença entre diálogo comunitário e sensibilização
**Duração**	20 Minutos
	**Discussão em plenário**<br><br>Pergunte aos participantes: *Na vossa opinião, quais são as diferenças entre um diálogo comunitário e uma sensibilização da comunidade?*
	**Notas**<br>O Formador escreve as respostas em um papel gigante em duas colunas.
	**Apresentação**<br>Em seguida, faça um resumo, comparando as respostas dos participantes com a tabela no cartaz abaixo. O Formador explica:<br>○ *"O diálogo comunitário" é diferente da mais clássica "sensibilização da comunidade" uma sessão de diálogo comunitário não é apenas sobre a partilha de informação e sensibilização, mas é essencialmente discutir, negociar e decidir as acções que precisam ser tomadas para melhorar a situação.*<br>○ *É feito para ser mais interactivo e orientado para a acção de sessões de sensibilização da comunidade. Discussão de viabilidade, aceitabilidade, barreiras para a adopção de comportamentos e práticas recomendadas, bem como maneiras de superar as barreiras e resistência.*<br>○ *No final de um diálogo comunitário, membros da comunidade devem chegar a acordo sobre medidas a serem tomadas e como essas acções serão monitoradas.*
	Antes do treino, o formador deve reproduzir o Cartaz fornecido abaixo em papel gigante utilizando marcadores coloridos.
	**Mensagem chave**<br>✓ *A sensibilização da comunidade destina-se a partilha de informação.*<br>✓ *O Diálogo Comunitário é uma discussão aberta e interactiva para resolver problemas de saúde através de acções simples que os membros da comunidade podem fazer juntos.*

Cartaz: Qual é a diferença entre uma sensibilização da comunidade e um diálogo comunitário?

Qual é a diferença entre uma sensibilização da comunidade e um diálogo comunitário?

	**Sensibilização comunitária**	**Diálogo Comunitário**
**Objectivo**	Sensibilização e partilha de informação sobre um tópico específico	Chegar a um acordo sobre as medidas a serem tomadas e como essas acções serão monitoradas
**Método**	Os líderes comunitários ou pessoa que tem bons conhecimentos sobre o assunto fala para a comunidade e os membros da comunidade podem fazer perguntas para esclarecimentos no final da palestra.	O líder comunitário facilita mas os membros da comunidade falam e discutem quando os problemas de saúde surgem entre as crianças e discutem o que os membros da comunidade podem fazer juntos para evitar ou resolver os problemas.
**Tópicos**	• Nova informação, serviços ou produtos<br>• Recomendações sobre comportamentos e práticas	• Discussão em profundidade de informações disponíveis/recebidos;<br>• Discussão sobre a viabilidade, aceitabilidade, barreiras para adopção de comportamentos e praticas recomendadas bem como as maneiras de superar as barreiras e resistência.

Enquanto sensibilização da comunidade destina-se a sensibilização e partilha de informação sobre um tópico específico, Diálogo Comunitário é uma discussão aberta, inclusiva e interactiva entre os membros da comunidade sobre o porquê de surgirem problemas de saúde entre as crianças e o que os membros da comunidade podem fazer juntos para evitar ou resolver problemas, melhorar a saúde, o bem-estar e futuro das crianças e da comunidade como um todo. No final de um diálogo comunitário, membros da comunidade devem chegar a acordo sobre medidas a serem tomadas e como essas acções serão monitoradas.

Actividade: Familiarização com o Guião de diálogo comunitário (20 min)

**Actividade**	Familiarização com o Guião
**Objectivos**	Os Participantes estão familiarizados com o Guião do diálogo comunitário
**Duração**	20 minutos
	**Apresentação**<br>O Formador apresenta o Guião do diálogo comunitário aos participantes.<br>*O Guião para o diálogo comunitário destina-se a ser usado por facilitadores comunitários, para se preparar para as sessões de diálogo com a comunidade. Este Guião é um memorando.*
	**Leitura**<br>O Formador pede os participantes para olhar a parte do guião "O que é diálogo comunitário" (10 minutos)
	**Discussão em plenário**<br>Em seguida, o Formador estimule a discussão:<br>• *Quem irá facilitar?*<br>• *Quem devera participar?*<br>• *Quanto tempo deverá durar?*<br>• *Quantas vezes deverá ser realizado?*<br>• *Onde pode ser realizado?*
	**Apresentação**<br>Fazer um sumário usando as páginas do Guião do diálogo comunitário.
	**Mensagem chave**<br>✓ *O diálogo com a comunidade não deve ser uma actividade artificial, mas tem que ser muito flexível, adaptado a cada comunidade.*<br>✓ *É importante **preparar – se de antemão**, de modo a seleccionar questões-chave dependendo de cada comunidade.*

Actividade: Os 10 passos para o diálogo (30 min)

**Actividade**	10 passos para um bom diálogo
**Objectivos**	Os Participantes estão familiarizados com os 10 passos
**Duração**	30 Minutos
	O formador apresenta os 10 passos:<br>1. Preparar; escolher um tema<br>2. Fazer ligação com membros influentes e comité de saúde<br>3. Marcar a data, hora e tema, e convidar os participantes.<br>4. Introdução<br>5. Explorar o tema – usando o Álbum Seriado<br>6. Identificar acções<br>7. Tomar decisões – usando a ficha de planificação e seguimento<br>8. Resumir – usando o Álbum Seriado<br>9. Agradecer<br>10. Preencher a Ficha de Monitoria
	**Prática**<br>O Formador pede aos participantes para formar grupos.<br>Cada grupo vai ter 10 minutos para ler e rever os 10 passos no Guião do diálogo comunitário. Cada grupo deve discutir como vai realizar os 3 primeiros passos na sua comunidade.
	**Discussão em plenário**<br>Em seguida, em sessão plenária, o Formador pede cada grupo para apresentar os 3 primeiros passos.<br>• *Como os 2 facilitadores vão partilhar os papéis? Como assegurar que os líderes comunitários são informados e vão apoiar?*<br>• *Quem vai ser convidado? Onde que vai ter lugar? Como mobilizar a comunidade para participar?*
	**Mensagem chave**<br>✓ Seria ideal que pelo menos um representante de cada família na comunidade participe nos diálogos. No entanto, o grupo de participantes por diálogo não deveria ser mais de 30 pessoas, porque é difícil todas pessoas ouvirem e participar quando o grupo é muito grande.<br>✓ Combinar diversos métodos de informação e mobilização da comunidade é mais eficiente. Exemplo: os facilitadores têm-se reunido com os LCs para confirmar a data, hora, local e tópico do DC, e seguidamente o LC comunica e coordena a disseminação de informação com outros membros influentes e chefes de 10 casas que andarem de casa em casa a convidar os cuidadores; disseminação de informação nas escolas, igrejas e outros espaços públicos como furos de água.

Sessão 4: Como conduzir o diálogo? (1 hora e meia)

Actividade: Apresentação do Álbum Seriado (40 min)

**Actividade**	Apresentação do Álbum seriado
**Objectivos**	Os facilitadores estão familiarizados com o conteúdo dos materiais
**Duração**	40 Minutos
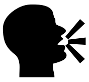	O formador apresenta os *materiais visuais para:*<br>○ *ajudar a comunidade a ganhar mais conhecimento sobre a doença durante o diálogo comunitário;*<br>○ *ajudar a comunidade a identificar acções saudáveis e tomar decisões para a melhoria da saúde.*
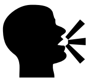	*O Álbum seriado tem na parte frontal uma ilustração que será usada como base para o debate do tema e no verso, tem um pequeno texto explicativo. As mesmas imagens cobrem:*<br>✓ *sinais e sintomas da doença*<br>✓ *práticas de risco*<br>✓ *práticas preventivas*<br>.
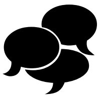	**Discussão em plenário**<br>Chuva de ideias: O Formador pergunta aos participantes:<br>• *Na vossa experiência, já usaram materiais similares?*<br>• *Quais são os benefícios das imagens para comunicar com os membros da comunidade?*
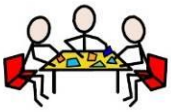	**Prática**<br>O Formador pede aos participantes para formar 3 grupos.<br>Cada grupo vai ter 10 minutos para rever 2 ou 3 páginas do Álbum.
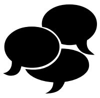	**Discussão em plenário (20 min)**<br>Em seguida, em sessão plenária, o Formador pede a cada grupo para apresentar as imagens/páginas. Fazer uma descrição da imagem e resumir a mensagem chave.
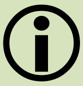	Esclarecer qualquer dúvida sobre as mensagens chaves.
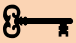	**Mensagem chave**<br>✓ *O Material que pode incentivar a participação das pessoas na discussão através de “tocar” e descrever o material (o material não deve ser um material de palestra)*<br>✓ *O Material que pode estimular a discussão sobre acções concretas que as pessoas podem tomar e as barreiras a levantar*

Actividade: Como usar o Álbum Seriado (10 min)

**Actividade**	Uso do álbum
**Objectivos**	Os Participantes estão familiarizados como usar o álbum durante o diálogo
**Duração**	10 Minutos
	**As imagens são úteis para estimular a discussão durante a fase de "explorar" o diálogo:**<br>• Não usar as imagens no começo do diálogo; deixar as pessoas explorar o tema usando as perguntas do guião de diálogo comunitário.<br>• Depois, mostrar uma página ao grupo.<br>• Pedir a uma pessoa para descrever a imagem<br>• Usar o Álbum Seriado para mostrar a todos a imagem que esta discutida<br>• Depois, pedir aos participantes para debaterem.<br>• Pedir a uma pessoa para ler o texto que consta no verso da carta. (Se não sabe ler, pede a uma outra pessoa do grupo para ajuda-la).<br>• Perguntar se todos entendem as frases, e clarificar se for necessário.<br>Repetir este processo com a página seguinte.
	**Discussão em plenário**<br>Perguntas e respostas
	**Mensagem chave**<br>**Mostrando uma imagem:**<br>• As imagens ajudam as pessoas a se lembrarem e a entenderem claramente o que você está dizendo.<br>• Mostrar a imagem duma forma que todos possam vê-la.<br>• Se o grupo fôr em número maior, circule a página pelos participantes para que todos possam ter oportunidade de olhar de perto a imagem no Álbum Seriado.<br><br>**Fazer perguntas:**<br>• Dar tempo às pessoas para responder a cada pergunta. Se ninguém responde, faça a mesma pergunta usando outras palavras.<br>• Quando alguém dá uma resposta que é errada, continue perguntando se alguém tem uma resposta diferente. Quando fôr dada a resposta correcta, repita-a, para que todos possam retê-la.<br>• Se você não conhece a resposta a uma pergunta (que é feita pelos participantes), diga a eles que irá consultar a um profissional de saúde e responderá a essa pergunta no próximo encontro.

Actividade: Habilidades de facilitação (30 min)

**Actividade**	Habilidades de facilitação
**Objectivos**	Os participantes pensam sobre habilidades de facilitação necessárias para conduzir um diálogo
**Duração**	30 Minutos
	**Discussão em plenário**<br>O Formador pede aos participantes para pensarem sobre as suas próprias experiências de facilitar discussões em grupo ou aconselhamento individual. Quais são algumas sugestões ou lições aprendidas que poderiam partilhar com todos os participantes no sentido de facilitar uma discussão?
	O Formador escreve as sugestões dos participantes no papel gigante.
	**Apresentação**<br>O Formador explica as dicas de facilitação, que estão no Guião: estas são destinadas a ajudar a criar um ambiente propício ao diálogo (usando o Cartaz a seguir)
	**Discussão em plenário**<br>O Formador pede aos participantes para compararem as suas sugestões com as dicas de facilitação.
	**Mensagem chave**<br>✓ *Se algumas questões técnicas não puderem ser respondidas durante o diálogo, estas devem ser registadas e depois colocadas a um profissional de saúde.*<br>✓ *Ajude todos os participantes a sentirem-se à vontade para expressar as suas opiniões e fazer perguntas. Assegurar que todos tenham oportunidade de ser ouvidos e que ninguém fale demais.*<br>✓ *Permaneça neutro, respeite as opiniões e preocupações dos membros da comunidade, e encoraje todas as pessoas na reunião a participarem.*<br>✓ *Se algumas pessoas não falarem, dê tempo para que elas também falem. Encorajar as pessoas mais caladas a contribuírem, mas não os force.*<br>✓ *Traga o grupo de volta ao tema central, se a discussão divergir do seu rumo.*<br>✓ *Lembre a todos que devem ouvir em silêncio e com respeito.*

Cartaz: Dicas adicionais para o facilitador do diálogo

- Cada diálogo pode durar até duas horas, se durar mais de duas horas os membros da comunidade podem deixar de prestar atenção.
- Use imagens para mostrar e estimular o debate. Organize o grupo de forma que fiquem todos o melhor instalado possível e que consigam ver as imagens. Se for possível convide-os a sentarem-se em círculo.
- Combine com o grupo para todos falarem alto de forma a se fazerem ouvir. Lembre a todos que devem ouvir com respeito.
- Comunique-se numa língua que todos percebam, use de preferência a língua local.
- Ajude todos os participantes a sentirem-se à vontade para expressarem as suas opiniões e fazerem perguntas. Assegurar que ninguém fale demais.
- Permaneça neutro, respeite as opiniões dos membros da comunidade.
- Encoraje todas as pessoas na reunião a participarem. Se algumas pessoas não falarem, dê tempo para que elas também falem, mas não os force.

Problemas que podem acontecer e como resolver:

- Se algumas questões não puderem ser respondidas durante o diálogo, os facilitadores deveriam anotar e depois perguntar a um profissional de saúde. As dúvidas que não forem respondidas no dia do diálogo deverão ser esclarecidas no próximo diálogo comunitário.
- Se as pessoas começarem a falar de outro assunto, pedir ao grupo para voltar ao tema em discussão, e propor que o outro assunto seja discutido num outro diálogo.

Actividade: Temas de discussão (10 min)

**Actividade**	Familiarização com Temas de discussão
**Objectivos**	Os Participantes estão familiarizados com os temas de discussão
**Duração**	10 minutos
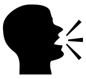	**Apresentação**<br>O Formador explica os 6 temas de discussão que são importantes para o projecto:<br>Não será possível de abordar todas perguntas sobre a bilharziose num único diálogo. E aconselhável de planificar um conjunto de diálogos, ao longo de 6 meses, para discutir da doença, com foco nas acções recomendadas para evita-la. Por exemplo:<br><br>Diálogo 1: Conhecer a bilharziose: como saber se tem a doença? Como se trata? Como se apanha? Como se evita?<br>Diálogo 2: Acção para evitar a bilharziose: como limitar contacto com água infectada?<br>Diálogo 3: Acção para evitar a bilharziose: manuseio seguro de água que pode ser infectada.<br>Diálogo 4: Acção para evitar a bilharziose: boas práticas de higiene<br>Diálogo 5: Antes duma campanha de distribuição de medicamentos: importância e procedimentos da campanha<br>Diálogo 6: Depois duma campanha de distribuição de medicamentos: Vantagens e desafios das campanhas, porque prevenir e evitar a reinfecção.
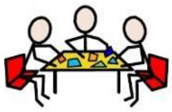	**Pratica**<br><br>O Formador pede aos participantes para formarem grupos.<br>Cada grupo vai escolher 1 tema e rever as perguntas para seleccionar as perguntas que podem usar durante um diálogo dentro da sua comunidade, dependendo de cada comunidade.<br>Cada grupo pode também criar perguntas. As perguntas devem ser abertas e focadas em pedir o ponto de vista dos participantes para incentivá-los a partilhar suas experiencias.
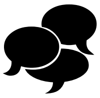	**Discussão em plenário**<br><br>Cada grupo fala em sessão plenária, cada grupo explica como e porquê seleccionou as perguntas.
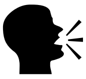	O Formador clarifica cada um dos três passos importantes durante o diálogo e o resultado de cada passo:<br>❒ *Explorar: para dar todos os participantes o mesmo nível de informação*<br>❒ *Identificar acções: para identificar o que as pessoas estão a fazer para prevenir e tratar as doenças, e identificar o que eles poderiam*

	fazer diferente ou mais.<br>❒ *Tomar decisões: para trabalhar em conjunto para resolver problemas, incluindo quem que vai fazer o que? Quando? Como?*
ⓘ	O Formador usa o Guião de Diálogo Comunitário.
🗝	Mensagem chave<br><br>✓ Os temas de discussão são apenas sugestões com exemplos de perguntas que poderiam ser discutidas com os membros da comunidade. Não é necessário para resolver todas as questões listadas no tópico em cada diálogo comunitário.<br>✓ O diálogo com a comunidade deve ser muito flexível, adaptado a cada comunidade. É importante preparar – se de antemão, de modo a seleccionar questões-chave dependendo de cada comunidade.<br>✓ O papel do facilitador é estimular a discussão com perguntas.

Actividade: Uso da ficha de planificação e seguimento (10 min)

**Actividade**	Familiarização com ficha de planificação e seguimento
**Objectivos**	Os Participantes sabem como usar a ficha durante o diálogo
**Duração**	10 minutos
	**Apresentação**<br>O Formador apresenta a ficha de planificação e seguimento dos diálogos.<br>O formador explique que essa ficha serve para recordar das decisões tomadas em cada diálogo e fazer o seguimento das acções implementadas e dos desafios na implementação das acções que foram concordadas.<br>A ficha será usada durante o diálogo nas fases de "identificar acções" e "tomar decisões".<br><br>***Identificar acções***<br>Em cada diálogo, enfocar a discussão sobre uma ou duas acções concretas para reduzir a doença na comunidade.<br>Usando a Ficha de Planificação e seguimento dos diálogos, pedir aos participantes de reportar sobre acções que eles concordaram a fazer no último diálogo, e discutir como ultrapassar dificuldades. Depois, pedir aos participantes que escolham juntos mais uma ou duas acções factíveis que devem ser feitas.<br><br>***Tomar decisões***<br>Os participantes devem concordar com os passos: Quem é que vai fazer o quê? Quando? Como?<br>Anotar na Ficha de planificação e seguimento dos diálogos as decisões tomadas, responsabilidades e ponto de situação se for o caso.
	**Discussão em plenário**<br>Esclarecer qualquer dúvida
	O Formador usa o modelo de plano no Anexo 3: Ficha de planificação e seguimento dos diálogos
	**O formador explique como preencher a ficha**<br><br>Na ficha, há uma linha para cada diálogo;<br>A ficha tem 6 linhas, que permite recordar decisões e seguimento de 6 diálogos.<br><br>O número na primeira coluna deveria corresponder a o número do diálogo (o primeiro, o segundo…etc); a ficha de monitoria também tem uma pequena caixa (acima) para colocar o número do diálogo; (o primeiro, o

	segundo…etc);<br>Como preencher as outras colunas:<br>Uma coluna para a data<br>Uma coluna para Decisões tomadas<br>Uma coluna para Registar o ponto de situação<br>Uma coluna para anotar desafios ou dificuldades, se for o caso
	✓ A ficha de planificação e seguimento deve ser usada durante todos os diálogos: usar a mesma ficha para recordar decisões, acções feitas, resultados e barreiras.<br>✓ Trazer esta ficha preenchida para as reuniões de balanço para rever o progresso e discutir como ultrapassar desafios.

Sessão 5: Prática (3 horas)

Actividade: Dramatização 1 (1 hora e meia)

**Actividade**	Dramatização
**Objectivos**	Os participantes praticam novas habilidades na preparação e realização dum diálogo comunitário.
**Duração**	90 minutos
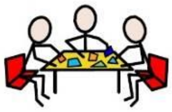	**Prática em grupo**<br>Divide os participantes em 2 grupos de 10 ou mais pessoas.<br>O Formador então pede a cada participante escolher um papel de participante num diálogo (líder comunitário, Mãe, Pai, pastor, APE, curandeiro, idoso, jovem... etc.).
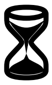	**Preparação**<br>Dar 10 minutos a quem assumir os papéis de facilitadores, em colaboração com os outros participantes (por exemplo, o líder escolhe um tema e chegar ao activista para se preparar, então ele vem visitar a mãe para informá-la sobre, etc.)
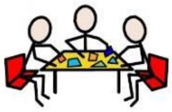	**Dramatização**<br>Quando o grupo estiver pronto, todos os participantes sentam-se em círculo e fazem uma representação de uma sessão de Diálogo comunitário (cerca de 40 min).
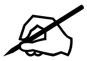	O Formador preenche a Ficha de Monitoria, logo após a sessão, e mantém-na para a próxima sessão.
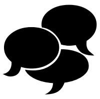	**Discussão em plenário**<br>Em seguida, o grupo discute o que correu bem ou não durante a dramatização.<br>• A sessão foi sob forma duma palestra? Ou duma forma participativa?<br>• O facilitador foi neutro e respeitou todas opiniões?<br>• O facilitador usou o álbum seriado?<br>• O facilitador usou a ficha de planificação?<br>• Quais são os pontos fortes e pontos fracos?<br>• Quais são as sugestões para melhoria?
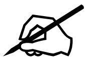	O Formador escreve as sugestões dos participantes no papel gigante em duas colunas (pontos fracos / pontos fortes)
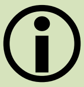	O Formador utiliza as "**dicas**" incluídas no Guião do diálogo comunitário para reflectir sobre habilidades de facilitação necessárias para estimular um diálogo participativo e animado.
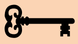	**Mensagem chave**<br>O Formador deve identificar as mensagens-chave que precisam ser repetidas.

Cartaz: Perguntas para avaliação

Nas habilidades de facilitação:

- Todos os participantes expressaram as suas opiniões e fizeram perguntas? Se não, porquê?
- O facilitador foi neutro e respeitou as suas opiniões?
- Como usou o Álbum?

No processo de diálogo comunitário:

- Os 3 passos principais do diálogo (explorar, identificar problemas, planear as acções) foram seguidas ou não?
- Qual foi o passo não abordado e porque?

Avaliação geral:

- Quais são os pontos fortes e pontos fracos?
- Quais são as sugestões para melhoria?

Actividade: Dramatização 2 (1 hora e meia)

**Actividade**	**Dramatização**
**Objectivos**	Os participantes praticam novas habilidades na preparação e realização de um diálogo comunitário.
**Duração**	**90 Minutos**
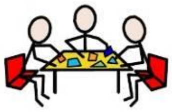	**Dramatização**<br><br>Quando o segundo grupo estiver pronto, todos os participantes sentam-se em círculo e fazem uma representação de uma sessão de Diálogo comunitário (cerca de 30 min).
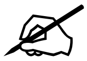	O Formador preenche a Ficha de Monitoria, logo após a sessão, e mantém-na para a próxima sessão.
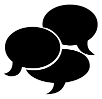	**Discussão em plenário**<br>Em seguida, o grupo discute o que correu bem ou não durante a dramatização, usando as perguntas no Cartaz.
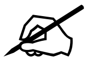	O Formador escreve as sugestões dos participantes no papel gigante em duas colunas (pontos fracos / pontos fortes)
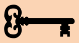	**Mensagem chave**<br>O Formador deve identificar as mensagens-chave que precisam ser repetidas.

Sessão 6: Planificação e Monitoria (2 horas e meia)

Actividade: Primeiro Dialogo (10 min)

**Actividade**	Apresentação do projecto durante o primeiro diálogo
**Objectivos**	Os facilitadores sabem que devem explicar aos participantes e ter consentimento da comunidade durante o primeiro diálogo, e sabem como fazer
**Duração**	**10 Minutos**
	O Formador explique:<br>Este projecto faz parte duma Pesquisa. E obrigatório obter o consentimento dos membros da comunidade em participar neste projecto.<br>Por isso, durante o primeiro diálogo, os facilitadores deveriam explicar o projecto durante a introdução ao diálogo e obter o consentimento dos participantes que eles concordam em participar neste projecto de pesquisa.<br><br>Mensagem chave a passar aos membros da comunidade:<br>• os diálogos são implementados como parte de uma pesquisa<br>• descrever brevemente os objectivos<br>• o facilitador irá informar os membros da comunidade que ele irá fornecer relatórios regulares no âmbito do esquema de monitoria.<br>• um membro da direcção provincial da saúde poderá observar alguns diálogos comunitários.<br>• O facilitador deverá pedir aos participantes e líderes comunitários para confirmar verbalmente o consentimento em participar.<br>**A decisão da comunidade deve ser escrita na ficha de monitoria da primeira sessão de diálogo.**
	Um exemplo de discurso dos facilitadores durante o primeiro encontro de diálogo comunitário esta apresentado no cartaz a seguir.
	**Discussão em plenário**
	**Mensagem chave**<br>✓ *Os **<u>facilitadores</u>** irão pedir consentimento dos membros da comunidade para participarem neste projecto durante o primeiro diálogo.*<br>✓ *Por isso, será importante assegurar que líderes e membros influentes da comunidade irão participar no primeiro diálogo*<br>✓ *Os **<u>facilitadores</u>** irão escrever a* **decisão da comunidade na ficha de monitoria da primeira sessão de diálogo.**

Exemplo de discurso de introdução durante o primeiro diálogo

Bom dia/boa tarde a todos. Meu nome é___________, sou membro do Comité de Saúde desta comunidade. Sejam bem-vindos ao nosso encontro onde vamos discutir sobre a saúde da nossa comunidade.

A nossa comunidade foi escolhida para fazer parte dum projecto de pesquisa que pretende compreender como o uso de diálogos comunitários pode ajudar para a prevenção e controle de doenças tropicais negligenciadas, na província de Nampula. As doenças tropicais negligenciadas são um grupo de doenças que afectam as pessoas que vivem em zonas rurais de Moçambique e de outros países, tal como a Bilharziose.

Este projecto esta a ser conduzido pela Direcção Provincial de Saúde de Nampula em parceria com a Malaria Consortium. O projecto vai durar cerca de 20 meses e será implementado nos distritos de Eráti, Mecuburi,Murrupula e Mogovolas.

No âmbito deste projecto, eu recebi um treino recentemente e vou organizar, junto com os outros membros do nosso comité de saúde e da nossa comunidade, encontros regulares de forma de diálogos comunitários participativos.

Durante esses diálogos, vamos falar sobre a Bilharziose que é uma doença que ataca muitas pessoas na nossa comunidade. Durante o nosso diálogo ou conversa vamos ver alguns desenhos ou imagem sobre situações que acontecem e que tem a ver com a bilharziose. Cada um de nós vai falar sobre o que consegue ver, o que acha sobre a imagem ou cada um de nos vai responder a perguntas que posso colocar durante o nosso encontro. No final vamos decidir o que e como poderemos fazer a partir de hoje para prevenir e tratar esta doença.

Durante os nossos diálogos, eu vou preencher uma ficha de monitoria, anotando os temas que nos discutimos, o número de participantes nos nossos encontros, e as decisões tomadas e resultados na melhoria das práticas de saúde para combater a bilharziose. Também, um supervisor da direcção provincial ou distrital da saúde poderia visitar-nos e observar um diálogo comunitário. Adicionalmente, no âmbito desta pesquisa, alguns membros da nossa comunidade poderiam ser entrevistados pela Malaria Consortium no âmbito de obter as perspectivas dos próprios membros da comunidade sobre esta abordagem de diálogos comunitários ou sobre a bilharziose.

Todas informações serão guardadas sem anotar qualquer nome das pessoas. A informação só estará disponível para os pesquisadores que trabalham no estudo.

Participar no projecto é voluntário. Se você decidir não participar, não haverá consequências negativas. Para se recusar a participar, não precisa de dar nenhum motivo ou justificação.

A participação no estudo é uma oportunidade para nos podermos contar as nossas experiências, opiniões e ideias.

Se você tiver alguma dúvida, por favor pergunte-nos.

(…)

Você também pode também entrar em contacto, se quiser, com nossos supervisores:

Malaria Consortium Nampula, Tel.: 84 389 7300

Direcção Provincial da Saúde Nampula, Tel.: 84 267 0917

Comité Nacional de Bioética para a saúde, Tel.: 824066350

(…)

Agora, queria pedir oralmente se todos nos aqui concordamos em participar?

(…)

Obrigado/a, agora podemos começar.

Actividade: Apoio do projecto aos facilitadores (10 min)

<table>
<tr><th>Actividade</th><th>Apoio do projecto aos facilitadores</th></tr>
<tr><td>Objectivos</td><td>Os participantes sabem qual apoio vão receber</td></tr>
<tr><td>Duração</td><td>10 Minutos</td></tr>
<tr><td>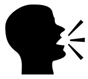</td><td>
O Formador apresenta: Três ferramentas serão usadas para monitoria e documentação dos diálogos comunitários.
Ficha de monitoria do Diálogo Comunitário
A ficha de monitoria será preenchido pelos facilitadores, após a realização de cada diálogo, e permitirá documentar dados básicos. Os facilitadores irão informar sobre os diálogos realizados durante as reuniões trimestrais de balanço.
Ficha de planificação e seguimento dos Diálogos
Os facilitadores deveriam trazer a ficha de planificação e seguimento em cada diálogo para registar as decisões tomadas, as responsabilidades, e fazer o seguimento nos seguintes diálogos para fazer o ponto de situação, junto com os membros da comunidade, sobre as acções e decisões tomadas e implementadas.
Reuniões de balanço ao nível distrital com facilitadores
O projecto irá organizar reuniões regulares de balanço ao nível distrital para permitir aos facilitadores encontrarem-se e partilhar experiências, boas práticas tal como soluções para ultrapassar dificuldades na implementação dos diálogos. Estas reuniões permitirão também esclarecer algumas dúvidas técnicas dos facilitadores sobre as DTNs e outros assuntos levantados pelas comunidades durante os diálogos. A primeira reunião de balanço será realizada em cada distrito depois dos primeiros 3 meses de implementação dos diálogos.
Observação de alguns Diálogos Comunitários
Os facilitadores irão receber um subsídio de comunicação (crédito) e serão incentivados a reportar questões técnicas e desafios logísticos urgentes ao supervisor a nível da província (ponto focal DTNs, DPS).
O supervisor irá também observar um mínimo de dois DCs por mês. DCs a serem observados serão escolhidos de forma aleatória.
</td></tr>
<tr><td>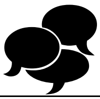</td><td>Discussão em plenário</td></tr>
</table>

	**Mensagem chave**<br>✓ *Os **facilitadores** são responsáveis por conduzir diálogos regulares e preencher um resumo de cada diálogo na ficha de monitoria.*<br>✓ Os ***facilitadores*** irão receber um subsídio de comunicação (crédito) a ser usado para reportar questões técnicas e desafios logísticos urgentes aos supervisores a nível da província (ponto focal DTNs, DPS).<br>✓ *Os **Supervisores** (distrito, DPS e Malaria Consortium) são responsáveis para responder a perguntas técnicas ou ajudar a resolver dificuldades;*<br>✓ *Os supervisores não vão visitar cada um dos facilitadores para visita de observação. Só algumas comunidades serão observadas; os supervisores irão informar com antecedência da visita.*<br>✓ *Reuniões de balanço: trazer registo dos diálogos realizados; compartilhar experiencia; resolver problemas juntos.*

Actividade: Ficha de Monitoria (20 min)

**Actividade**	Ficha da Monitoria
**Objectivos**	Os participantes sabem preencher a ficha de monitoria
**Duração**	**20 Minutos**
	O Formador apresenta a Ficha da Monitoria do Diálogo. Cada participante deve receber uma cópia da Ficha. O formador explica:<br>• *Ao mínimo, um diálogo deve ser realizado uma vez em cada mês (ainda melhor 2 vezes) ou sempre que os membros da comunidade ou líderes da comunidade sentir que há uma necessidade de discutir um tópico.*<br>• *Esta ficha destina-se a recolher informações para saber se o diálogo está acontecendo ou não, e formar uma base para a revisão com facilitadores das questões-chave identificadas ao nível da comunidade e como elas poderiam ser resolvidas. Isto pode ser feito durante as visitas de monitoria ou reuniões de balanço.*<br>O Formador lê alto e explica como ele/ela preencheu a Ficha após as 2 dramatizações.
	**Discussão em plenário**
	**Mensagem chave**<br>✓ *Os **facilitadores** deverão preencher a Lista logo **após** o diálogo, não escrever demais durante o debate (porque é difícil de ouvir, falar e escrever ao mesmo tempo), mas, logo que os participantes deixem o local, preencha a Ficha para que você não se esqueça o que aconteceu e quais decisões foram tomadas, se você não se lembra o suficiente para escrever em cada pergunta, apenas deixe em branco.*<br>✓ *Os **facilitadores** deverão guardar as fichas com eles até que sejam colectadas e discutidas pelos Equipes dos distritos.*<br>✓ *Há uma secção, na Ficha de monitoria, para **recordar as questões técnicas** que não foram respondidas durante o diálogo e outras observações.*<br>✓ *Os facilitadores vão receber crédito telefónico para enviar as perguntas que precisam de ser respondidas urgentemente aos supervisores.*<br>✓ *Os supervisores vão fazer actualizações técnicas trimestrais durante reuniões de balanço com base nas perguntas e questões levantadas.*
	**Usa a Ficha de Monitoria no Anexo 2 deste manual**

Actividade: Planificação (60 min)

**Actividade**	Plano de Acção
**Objectivos**	Os participantes desenham um plano de acção para a implementação de diálogos comunitários nas suas comunidades.
**Duração**	60 Minutos
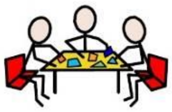	**Pratica**<br>Forma grupos de trabalho por cada área de saúde. Cada grupo deve desenvolver o seu plano de trabalho para 6 meses, usando o modelo abaixo.<br>• Mapeamento: Como os facilitadores vão assegurar que todas famílias da comunidade são abrangidas?<br>• Quantas vezes o diálogo devera ser realizado? Com quem?<br>• Onde pode ser realizado?<br>• Quais serão os temas?<br>• Quais líderes ou membros influentes ou outros membros dos comités de saúde poderem ajudar? E como?
	20 minutos
	**Discussão em plenário**<br>Quando os grupos estiverem prontos, todos os participantes sentam em círculo e cada grupo faz uma apresentação do seu plano.
	**Mensagem chave**<br>Mantenha o seu plano realista.
	**Usa o modelo de plano no Anexo 3: Ficha de planificação e seguimento dos diálogos**

Actividade: Encerramento do treino (60 min)

**Actividade**	**Encerramento**
**Objectivos**	Os participantes são capazes de:<br>• Realizar o Pós-Teste sobre os conhecimentos que adquiriram durante a formação<br>• Avaliar a formação<br>• Receber os certificados de participação e camisetas
**Duração**	60 Minutos
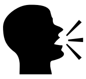	O Facilitador distribui as fichas individuais de pós-teste
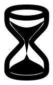	15 minutos
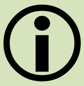	O Facilitador deve compilar e analisar os resultados destes testes e comparar com resultados do pré-teste para verificar se os participantes ganharam conhecimentos, e também para incluir no relatório da formação.
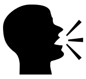	O Facilitador distribui as fichas de avaliação individual do treino
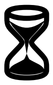	15 minutos
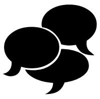	Olhando para o cartaz dos Objectivos do treinamento, o treinador pede aos participantes para avaliar se os objectivos foram cumpridos ou não cumpridos.
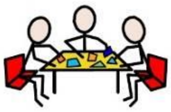	O facilitador pede aos participantes para pensarem sobre o seminário. Peça aos participantes para formar um círculo e cada um dizer uma coisa de que eles gostavam e uma coisa que eles não gostaram.
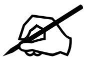	O facilitador escreve as sugestões dos participantes no papel gigante em duas colunas (pontos fracos / pontos fortes); Agradeça aos participantes.
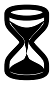	15 minutos
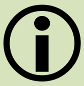	O Facilitador pode usar estas sugestões para escrever o relatório do treinamento.

	Dar Certificado de participação e uma camiseta a cada formando.
	15 minutos
	Encerramento oficial

Anexos

Anexo 1: Ficha de informação sobre o projecto

Diálogos Comunitários para Prevenção e Controlo das Doenças Tropicais Negligenciadas, em Moçambique

1. Que projecto é este?

É um projecto de pesquisa operacional que foi concebido pela Malaria Consortium em parceria com a Direcção Provincial de Saúde de Nampula.

2. O que o projecto pretende?

O projecto pretende por meio de várias actividades saber se o modelo de diálogos comunitários é uma ferramenta útil para reforçar a participação das comunidades na melhoria das suas práticas de Saúde para prevenção e controlo das doenças tropicais negligenciadas (DTNs).

3. O que são diálogos comunitários?

Diálogos comunitários acontecem quando um grupo de pessoas da comunidade se reúne para discutir assuntos que preocupam a sua comunidade. É diferente de uma palestra, de uma actividade de sensibilização pois, nestas as pessoas são ouvintes de uma pessoa que vem falar a comunidade. Nos diálogos comunitários as pessoas partilham suas ideias e experiências sobre o tema ou assunto de formas a tomarem melhores decisões sobre os assuntos tratados.

4. O que são doenças tropicais negligenciadas?

São um grupo de doenças transmissíveis que ocorrem principalmente em ambientes de calor e humidade das zonas do clima tropical, como em Moçambique. Afectam principalmente pessoas em áreas remotas. A bilharziose é uma dessas doenças.

5. Qual vai ser a doença mais focalizada?

O enfoque será para a bilharziose que é muito frequente e está muito espalhada na província de Nampula, mas esta doença pode ser facilmente tratada.

6. Quanto tempo o projecto vai durar?

O projecto vai durar cerca de 20 meses e será implementado nas comunidades dos distritos de Eráti, Mecuburi,Murrupula e Mogovolas.

7. Que actividades serão desenvolvidas?

Serão desenvolvidas as seguintes principais actividades:

7.1. Inquérito de base sobre conhecimentos, atitudes e práticas

Um grupo de pesquisadores treinados pela equipe da Malaria Consortium vai realizar entrevistas usando a língua local. Algumas comunidades serão seleccionadas de forma aleatória e um pequeno número de membros da comunidade será entrevistado. A entrevista será feita a um indivíduo que pode responder a perguntas sobre a saúde de sua família e terá uma duração de cerca de 45 minutes.

7.2. Recrutamento e treinamento de facilitadores comunitários

Os facilitadores comunitários é que vão moderar os diálogos comunitários. Estes serão escolhidos dentro da comunidade através dos Comités de Saúde ou dos líderes comunitários ou dos APEs.

7.3. Implementação dos diálogos comunitários (primeira fase)

Os facilitadores comunitários vão conduzir logo após a sua formação, uma ou duas vezes por mês durante o tempo da duração do projecto.

7.4. Avaliação

Serão usadas informações produzidas e registadas pelos facilitadores comunitários, informações produzidas e registadas pelos supervisores. Adicionalmente serão entrevistados facilitadores comunitários e pequeno número de membros da comunidade. Esta avaliação será conduzida pelo Oficial de pesquisas da Malaria Consortium nas comunidades onde os diálogos ocorrerem.

7.5. Implementação dos diálogos comunitários (segunda fase)

Após a avaliação serão feitas algumas revisões nos materiais e técnicas que os facilitadores vão usar de acordo os resultados que serão obtidos. Na segunda fase os facilitadores comunitários vão conduzir os diálogos comunitários seguindo as recomendações da revisão.

7.6. Inquérito final sobre conhecimentos, atitudes e práticas (CAP)

Com a mesma metodologia do inquérito de base, a informação gerada neste servirá para comparar com as informações geradas do inquérito base de forma a avaliar se os diálogos comunitários produziram melhorias nas práticas sobre saúde na comunidade.

8. Que benefícios o projecto pode trazer para as comunidades?

Este projecto vai despertar as pessoas em relação as DNT que são frequentes e alastradas nas nossas comunidades. As pessoas das nossas comunidades terão mais conhecimentos sobre a bilharziose, suas causas, sintomas, medidas preventivas e o que fazer individual e colectivamente para acabar com esta e outras doenças nas nossas comunidades.

Contactos:

845810401-Valdimar Antonio (Malaria Consortium)

845835570 - Dr. Ercílio Salomão (Direcção Provincial de Saúde-Nampula)

Anexo 2: Ficha de monitoria

Ficha de Monitoria<br>Diálogo Comunitário	Numero
**Nome dos Facilitadores**	
**Distrito**	
**Posto Administrativo**	
**Localidade**	
**Povoado ou Comunidade**	

Local do diálogo: ....

Data do diálogo: ....

Hora de Inicio ……………………………….…. Hora de fim ……………………………………….

Número de participantes: ....

Homens: .... Mulheres: ....

Explorar o tema: Quais foram os temas para o diálogo?

....

....

....

....

Observações:

---

1) Sobre logística

....

………

………

2) Sobre o guião de diálogo ou com o álbum seriado

………

………

………

3) Sobre perguntas técnicas (escreve aqui todas as questões que você ou os membros da comunidade não tem certeza ou não sabem a resposta correta)

………

………

………

4) Outras observações ou sugestões

………

………

………

Próximo diálogo:

Quando será? ………………………………

Qual será o tema? ………………………………

Anexo 3: Ficha de planificação e seguimento dos diálogos

Numero	Data	Decisões tomadas	Responsabilidades: Quem é que vai fazer, e quando?	Ponto de situação ou Resultados	Desafios na implementação das decisões

Anexo 4: Ficha de presença – Treino em Diálogos Comunitários

Distrito de ___________________________

	Nome	Posição social na comunidade/bairro ou profissão	Posto Administrativo	Localidade Comunidade/bairro	Contacto	Assinatura
1						
2						
3						
4						
5						
6						
7						
8						
9						
10						
11						

12						
13						
14						
15						
16						
17						
18						
19						
20						
21						
22						
23						
24						
25						
26						

27						
28						
29						
30						

Verificado por (Nome do Formador): ____

Data: ____

Assinatura: ____

Anexo 5: Ficha individual de PRÉ / PÓS - TESTE

Nome: ________________

Numero

Data: ________________

Por cada pergunta, marcar circulo a sua resposta (ou suas respostas)

1. **Como você apanha bilharziose?**

(Seleccione todas respostas adequadas)

Bebendo água contaminada .... Pescando na água infectada

Comendo alimentos não lavados Falta de higiene/hábitos de saneamento

Tomando banho/nadando no rio Trabalhando em campos de arroz/agricultura

Relações Sexuais/Via Sexual .... Partilhando talheres/pratos Não sabe

2. **Como uma pessoa infectada pode contribuir para a propagação da bilharziose?**

(Seleccione todas respostas adequadas)

Pessoa infectada urinando pelas águas Relações Sexuais/Via Sexual

Pessoa infectada defecando pelas águas Não sabe

3. **Como pode evitar ter bilharziose?**

(Seleccione todas respostas adequadas)

Proteger a fonte de água .... Beber água tratada ou fervida

Não nadar em águas paradas ..... Usar latrinas Não sabe

4. **Quais são os principais sintomas da bilharziose?**

(Seleccione todas respostas adequadas)

Dor ao urinar .... Sangue na urina ou nas fezes Fadiga/Cansaço ....

Barriga grande/Barriga “de água” Diarreia Náusea/vómitos

Borbulhas saem no corpo .... Não sabe

5. **Como se trata a bilharziose?**

(Seleccione apenas 1 resposta)

Ao curandeiro

Com uma dose única de medicamento na Unidade Sanitária

Não se cura

6. **Quais medicamentos são distribuídos durante as campanhas contra a bilharziose?**

(escreve)

7. Quem deve tomar o medicamento durante as campanhas contra a bilharziose?

(Seleccione apenas 1 resposta)

Só crianças

Só adultos

Todas as pessoas a partir de 5 anos de idade

8. Apenas crianças fracas e pessoas idosas podem morrer de bilharziose

(Seleccione apenas 1 resposta)

Sim   Não

9. Diga 3 medidas de como prevenir a bilharziose

(escreve)

---

1) ……………………

2) ……………………….

3) ……………………………

10. **Quais os 3 passos essenciais de discussão durante um diálogo comunitário?**

(Seleccione apenas 3 respostas)

Marcar a hora   Explorar o Tema   Identificar problemas

Tomar decisões   Agradecer aos participantes

Anexo 6: Respostas as perguntas de PRÉ / PÓS - TESTE

1. **Como você apanha bilharziose? (pontuação total 3: 0,5 ponto por resposta correcta)**

(Seleccione todas respostas adequadas)

Bebendo água contaminada
Pescando na água infectada
Comendo alimentos não lavados
Falta de higiene/hábitos de saneamento
Tomando banho/nadando no rio
Trabalhando em campos de arroz/agricultura
Relações Sexuais/Via Sexual
Partilhando talheres/pratos
Não sabe

2. **Como uma pessoa infectada pode contribuir para a propagação da bilharziose? (pontuação total 1: 0,5 ponto por resposta correcta)**

(Seleccione todas respostas adequadas)

Pessoa infectada urinando pelas águas
Pessoa infectada defecando pelas águas
Relações Sexuais/Via Sexual
Não sabe

3. **Como pode evitar ter bilharziose? (pontuação total 3: 1 ponto por resposta correcta)**

(Seleccione todas respostas adequadas)

Proteger a fonte de água
Beber água tratada ou fervida
Não nadar em águas paradas
Usar latrinas
Não sabe

4. **Quais são os principais sintomas da bilharziose? (pontuação total 3: 0,5 ponto por resposta correcta)**

(Seleccione todas respostas adequadas)

Dor ao urinar
Sangue na urina ou nas fezes
Fadiga/Cansaço
Barriga grande/Barriga "de água"
Diarreia
Náusea/vómitos
Borbulhas saem no corpo/corpo fica vermelho
Não sabe

5. **Como se trata a bilharziose? (pontuação: 1 ponto)**

(Seleccione apenas 1 resposta)

Ao curandeiro
Com uma dose única de medicamento na Unidade Sanitária
Não se cura

6. **Quais medicamentos são distribuídos durante as campanhas contra a bilharziose? (pontuação: 1 ponto)**

(escreve) **Praziquentel**

---

7. **Quem deve tomar o medicamento durante as campanhas contra a bilharziose? (pontuação: 1 ponto)**

(Seleccione apenas 1 resposta)
Só crianças

Só adultos

Todas as pessoas a partir de 5 anos de idade

8. **Apenas crianças fracas e pessoas idosas podem morrer de bilharziose (pontuação: 1 ponto)**

(Seleccione apenas 1 resposta)
Sim
Não

9. **Diga 3 medidas de como prevenir a bilharziose (pontuação total 3: 1 ponto por resposta correcta)**

(escreve)

---

4) **Não ficar na água parada durante muito tempo.**
5) **Também, deve se cuidar bem da água e da higiene individual e colectiva:**
6) **Participar nas campanhas do Ministério da Saúde que tratam dessa doença:**

10. **Quais os 3 passos essenciais de discussão <u>durante</u> um diálogo comunitário? (pontuação 3: 1 ponto por resposta correcta)**

(Seleccione apenas 3 respostas)
Marcar a hora

Explorar o Tema

Identificar problemas

Tomar decisões

Agradecer aos participantes

Anexo 7: Ficha de compilação de resultados individuais do pre e pós test

***Instruções**: para cada participante, inserir na coluna "pré-teste" a pontuação total obtida pelo participante durante o pré-teste no início do treino; inserir na coluna "pós-teste" a pontuação total obtida pelo participante durante o pós-teste no fim do treino; calcular a evolução como a seguir; (pontuação do pós-teste) menos (pontuação do pré-teste).*

Número de Ordem	Nomes dos participantes	Pré Teste	Pós Teste	Evolução
1				
2				
3				
4				
5				
6				
7				
8				
9				
10				
11				
12				
13				
14				
15				
16				
17				
18				
19				
20				
21				
22				
23				
24				
25				
	**TOTAL**			

Nome do Formador: …………………………………………………………… Data: ……………………………………..

Assinatura:…………………………………………………………………………………

Anexo 8: Ficha de avaliação individual do treino

Este questionário pretende aferir o seu nível de satisfação da acção de formação “**Técnicas de Supervisão de Apoio para Agentes Polivalentes Elementares e Manejo Integrado de Casos na Comunidade**”. O presente questionário é anónimo, não existem para o efeito respostas certas ou erradas, tendo como principal objectivo conhecer a sua opinião. Com base na seguinte escala:

Bom ☺ Médio 😐 Não bom ☹

**Perguntas**	☺	😐	☹
Como foi o formador?			
Motivado e motivador			
Expõe os temas de forma clara			
Dinâmico e criativo			
Metodologia de ensino			
Como foi a logística?			
Refeições			
Organização de espaço / da sala			
Duração das sessões			
Como foi o conteúdo de aprendizagem?			
Ganhou novos conhecimentos sobre a Bilharziose			
Ganhou novos conhecimentos sobre a facilitação de encontros comunitários			
Criou-se Capacidade de aplicação dos conhecimentos			
Eu me sente capaz de falar com outras pessoas da comunidade sobre a Bilharziose			
Eu me sente capaz de organizar e facilitar diálogos sobre a Bilharziose na minha comunidade			

Por fim, escreva se necessário, aspectos a melhorar e/ou sugestões:

____

____

____

____.

Obrigado pela sua atenção!

Anexo 9: Ficha de resumo da avaliação do treino

O formador deve inserir em cada coluna o numero de participantes que jugaram cada item como Bom ☺, Médio 😐, ou Não bom ☹. Verifique que o total corresponde ao total de participantes que preencheram a ficha de avaliação do treino.

**Perguntas**	☺	😐	☹	**Total**
Como foi o formador?				
Motivado e motivador				
Expõe os temas de forma clara				
Dinâmico e criativo				
Metodologia de ensino				
Como foi a logística?				
Refeições				
Organização de espaço / da sala				
Duração das sessões				
Como foi o conteúdo de aprendizagem?				
Ganhou novos conhecimentos sobre a Bilharziose				
Ganhou novos conhecimentos sobre a facilitação de encontros comunitários				
Criou-se Capacidade de aplicação dos conhecimentos				
Eu me sente capaz de falar com outras pessoas da comunidade sobre a Bilharziose				
Eu me sente capaz de organizar e facilitar diálogos sobre a Bilharziose na minha comunidade				

Principais sugestões da parte dos participantes no treino:

....

....

....

Nome do Formador: .... Data: ....

Assinatura:..

Anexo 10: Modelo de relatório do treino

**Nome do Formador**: _________________
**Distrito**: _________________
**Datas** do treino: do dia_____-até o dia ____ do mês de ___________ do ano 2014
**Local** do treino: _________________
**Numero total de participantes**:__________________<br>Homens: ____________ Mulheres: ______________
**Ficha de presença anexada?** SIM___ NÃO ___
**Ficha de compilação de resultados individuais do pré e pós teste anexada?** SIM___ NÃO ___
**Ficha de resumo da avaliação do treino anexada?** SIM___ NÃO ___
**Pontos fortes:**

**Pontos fracos:**
**Recomendações**
**Data**
**Assinatura**

O relatório deve ser preenchido e enviado dentro de 5 dias depois de cada treino a:

84 851 04 01 - Valdimar António (Malaria Consortium)

ou

845835570 - Dr. Ercílio Salomão (DPS-Nampula)

Anexo 11: Modelo de certificado de participação no Treino

Cada participante deveria receber um certificado assinado pela Malaria Consortium e DPS-Nampula, com o seu nome.

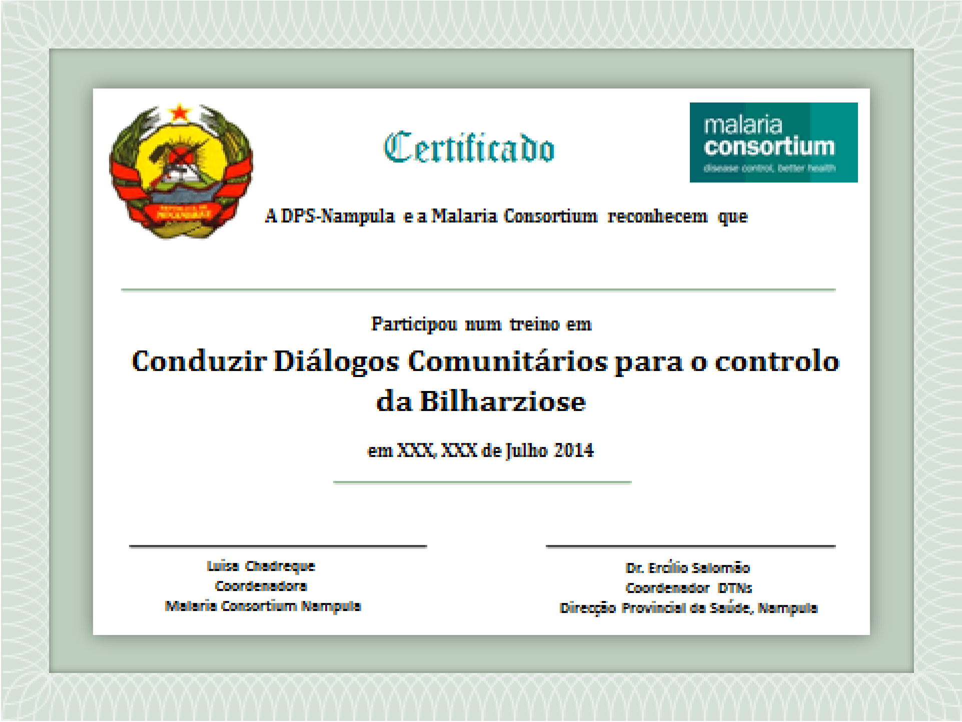

Supplement: S1 Appendix — (PDF) [file pone.0255647.s001.pdf]
