# Supplementary material for: Evaluating the feasibility and acceptability of a community dialogue intervention in the prevention and control of schistosomiasis in Nampula province, Mozambique
Source: PLoS One. 2021 Aug 5;16(8):e0255647. doi: 10.1371/journal.pone.0255647 (PMC8341517; doi:10.1371/journal.pone.0255647)

Vamos combater a Bilharziose

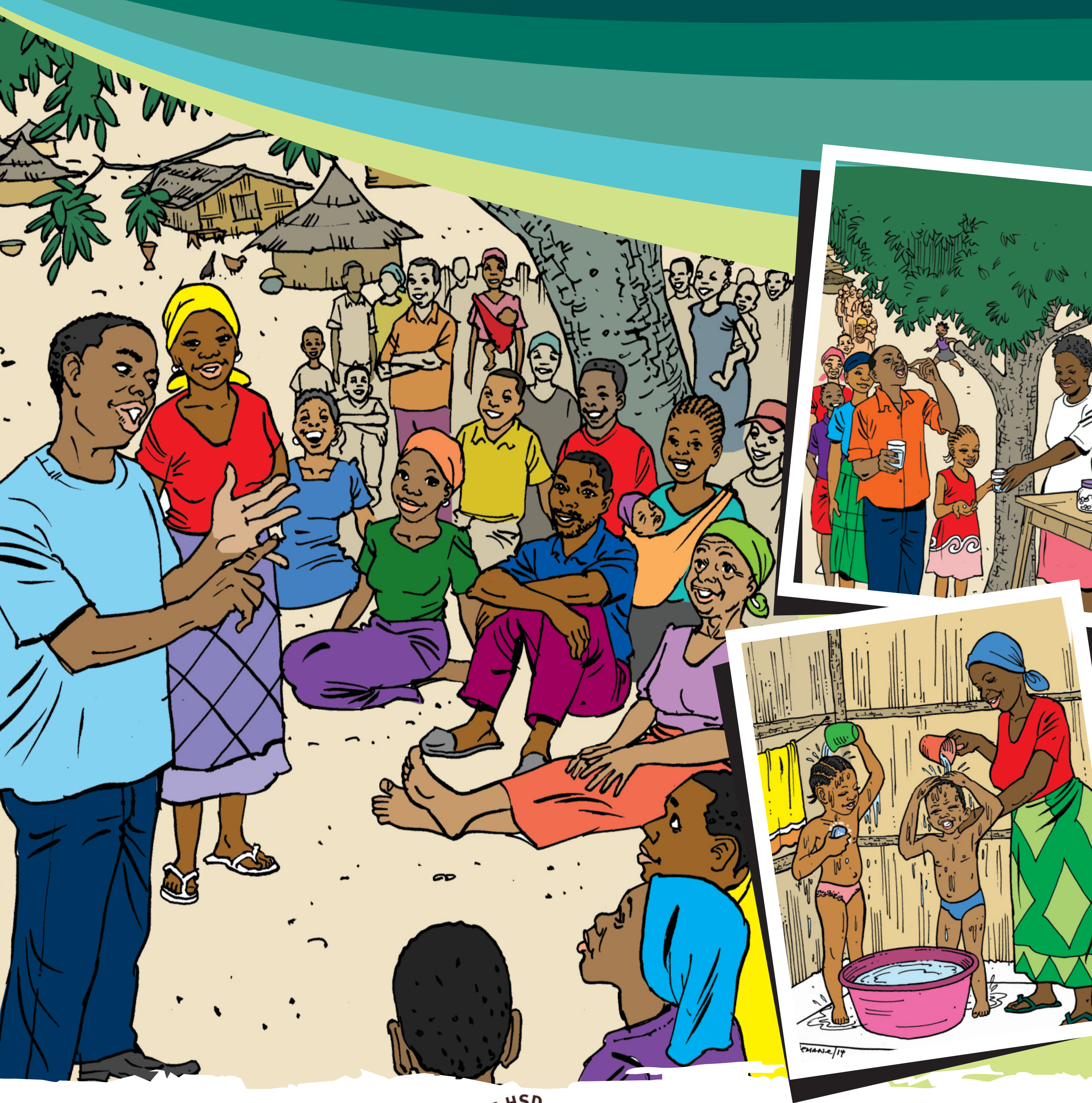

malaria consortium
disease control, better health

COMDIS-HSD • RESEARCH & DEVELOPMENT FOR EFFECTIVE SERVICE DELIVERY

MINISTÉRIO DA SAÚDE
DIREÇÃO PROVINCIAL DE SAÚDE - Nampula

Diálogos comunitários para prevenção e controlo das doenças tropicais negligenciadas, em Moçambique

O presente Álbum Seriado foi desenvolvido para ajudar os facilitadores de diálogos comunitários a estimular a discussão participativa sobre bilharziose e acções simples que as famílias e comunidades podem tomar para prevenir e tratar essa doença.

FICHA TÉCNICA

Autores: Malaria Consortium e DPS Nampula

Ilustrações e *layout*: Zacarias Chemane

Tiragem: 200 Exemplares

2ª Edição: 2015

Projecto: Diálogos comunitários para prevenção e controlo das doenças tropicais negligenciadas, em Moçambique

Financiamento: Fundação Bill & Melinda Gates (www.grandchallenges.org) e COMDIS-HSD (comdis-hsd.dfid.gov.uk)

O que é Bilharziose?

A bilharziose é uma doença muito comum nas nossas comunidades, onde pessoas usam a água dos rios, lagoas ou charcos. Muitas pessoas estão infectadas com a doença: adultos, crianças, idosos. Mas muitas pessoas não sabem que têm bilharziose, porque a doença pode ser confundida com outros problemas. A bilharziose pode ser muito grave a longo prazo, incluindo a infertilidade, e até matar, se não for tratada. Mas existem soluções para evitar e tratar a bilharziose.

É importante que os membros da comunidade partilhem informações e experiências sobre a doença para resolver juntos este problema de bilharziose, através de soluções comuns e factíveis, para melhorar a saúde da comunidade.

O que é Diálogo Comunitário?

Um diálogo comunitário acontece quando os membros da comunidade se reúnem para discutir questões de seu interesse.

"O diálogo comunitário" é diferente da "sensibilização da comunidade" ou palestra. Numa palestra, usualmente, uma pessoa fala para a comunidade sobre um tema. Num diálogo, todos os participantes podem partilhar informação e experiências sobre o tema. No final do diálogo, os participantes devem chegar a acordo sobre acções a serem tomadas pela comunidade e como essas acções serão implementadas pela própria comunidade para contribuir resolver os problemas de saúde.

10 Passos para Conduzir um Diálogo

Antes do diálogo:

1. Conhecer o seu Guião de Diálogo Comunitário, e as imagens que podem ser usadas para estimular a discussão.

2. Falar com líderes, membros influentes e membros do comité de saúde que podem ajudar no diálogo.

3. Marcar a data, hora e tema, com todos os participantes.

No dia do diálogo:

4. Introdução

Depois dos cumprimentos dos participantes, apresentar o tema e o objectivo do diálogo. Verificar o cumprimento das decisões tomadas no encontro anterior.

5. Explorar o tema

Estimule um debate, deixando as pessoas a partilhar os seus conhecimentos e experiências pessoais sobre a bilharziose, usando exemplos de perguntas no seu Guião de Dialogo, e mostrando imagens do Álbum.

Pode distribuir algumas imagens a pessoas do grupo. Pedir a cada pessoa para descrever a imagem e mostra-la ao grupo. Depois, pedir aos participantes para debaterem.

Antes de passar para a imagem seguinte confirme se foram discutidas as informações básicas (texto atrás das imagens do seu Álbum).

6. Identificar acções

Pedir aos participantes que escolham uma ou duas acções concretas e factíveis que devem ser feitas pela própria comunidade para reduzir a doença na comunidade.

Usando a Ficha de Planificação e Seguimento dos diálogos, pedir aos participantes de reportar sobre acções que eles concordaram a fazer no último diálogo, e discutir como ultrapassar dificuldades. Depois, pedir aos participantes que escolham juntos mais uma ou duas acções factíveis que devem ser feitas.

7. Tomar decisões

Os participantes devem concordar com os passos: Quem é que vai fazer o quê? Quando?

Anotar na Ficha de Planificação e Seguimento dos diálogos as decisões tomadas, responsabilidades dos membros da comunidade e ponto de situação se for o caso.

8. Resumir as informações usando o Álbum junto aos participantes. Lembrar a todos as decisões tomadas.

9. Agradecer aos participantes por terem partilhado as suas opiniões e marcar o próximo encontro.

No fim do diálogo:

10. Preencher a Ficha de Monitoria do diálogo e guardar até alguém do projecto pedir.

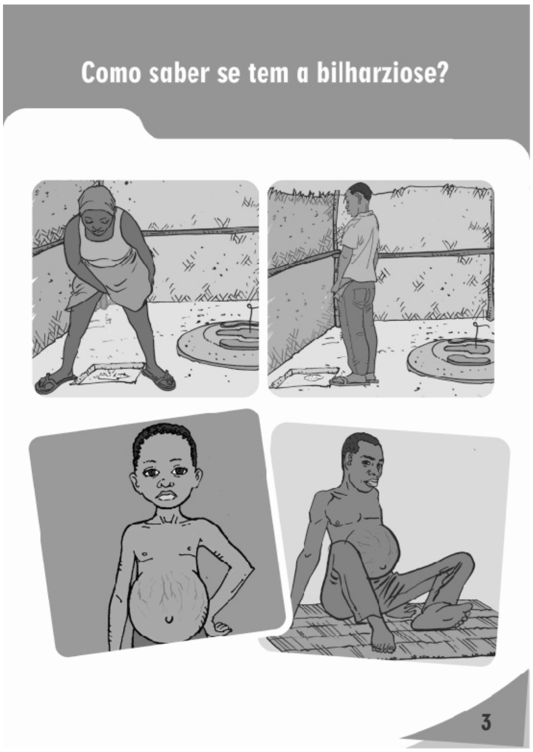

Como saber se tem a bilharziose?

A doença afecta a bexiga, e as vezes a barriga, por causa de bichos dentro do corpo: o primeiro sinal de bilharziose é ter sangue na urina e dor ao urinar; depois, outros sinais são: a barriga pode ficar muito grande (barriga de água), ou com dores; a pessoa doente pode ter diarreia.

Muitas pessoas podem confundir esta doença com outros problemas, particularmente doenças sexuais.

Pessoas que tem esse tipo de sinais no corpo ou ao urinar deveriam ir a Unidade Sanitária, para fazer um teste simples de urina que vai descobrir se a pessoa tem a bilharziose ou outra doença.

Como saber se tem a bilharziose?

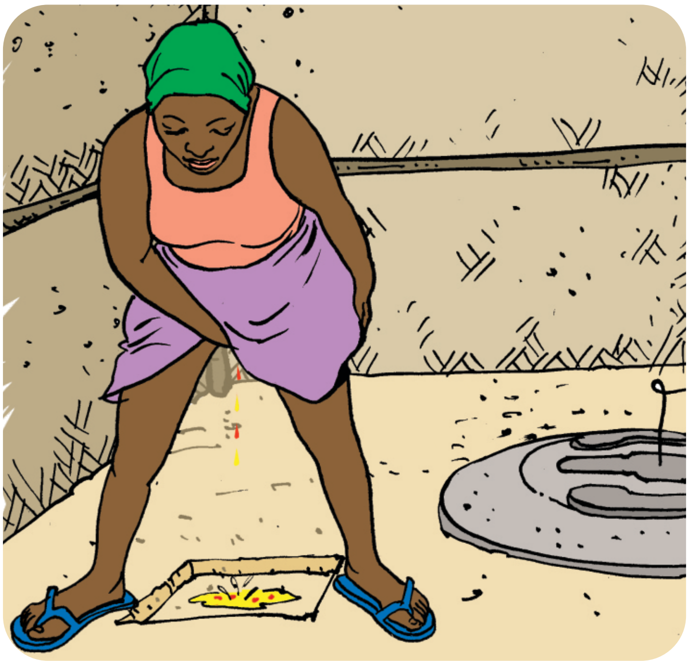

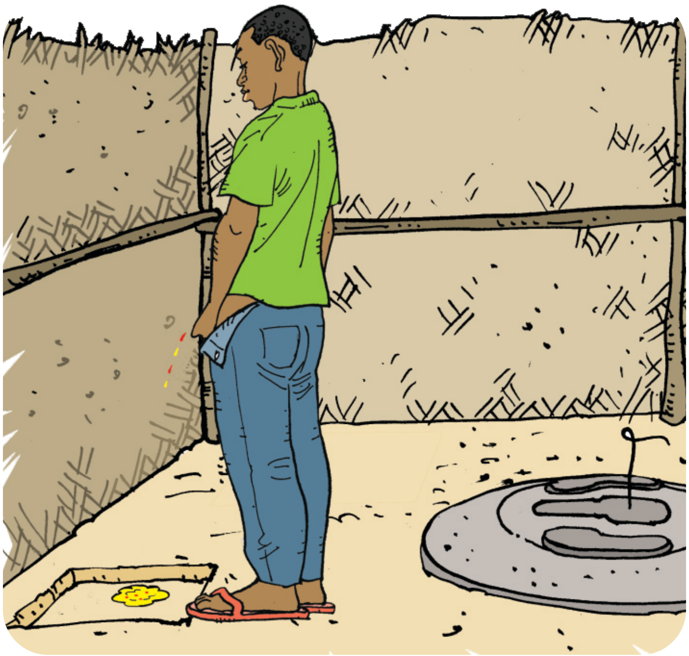

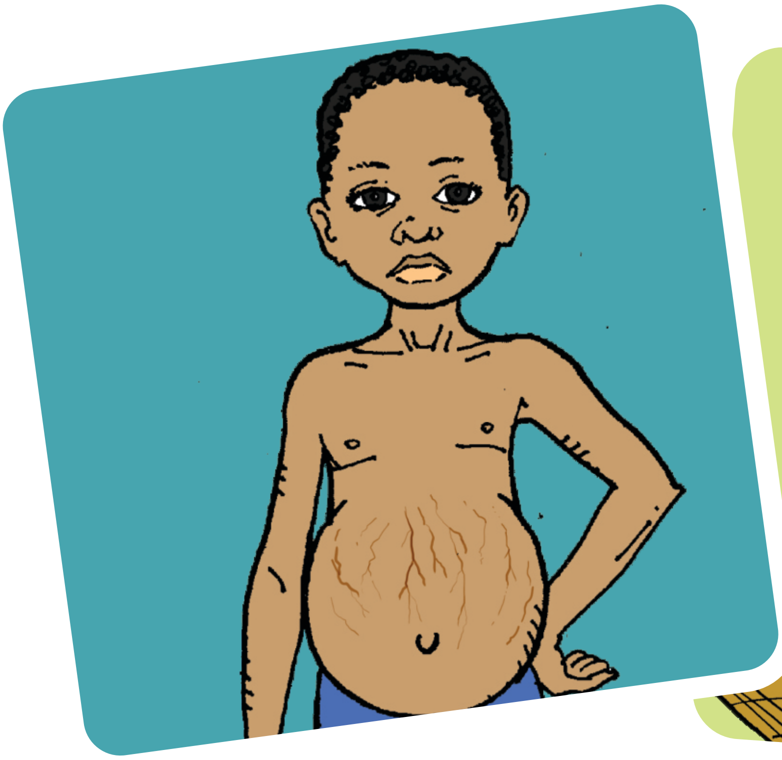

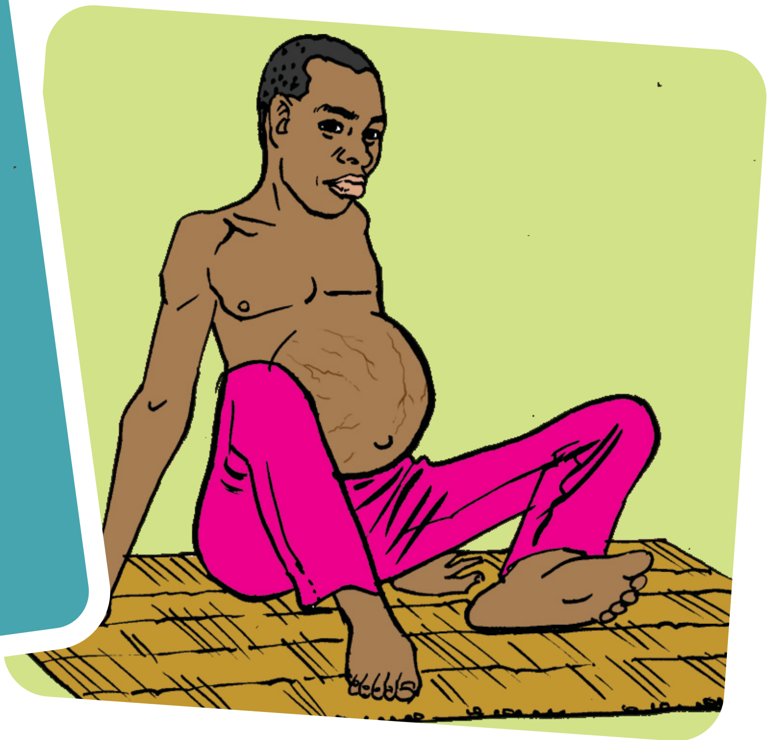

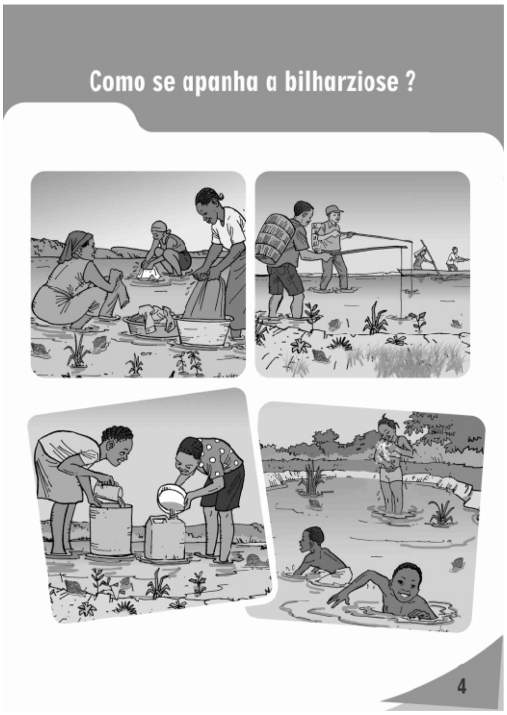

Como se apanha a bilharziose?

A doença se apanha através de pequenos bichos que vivem dentro de caracol nas águas paradas, como pequenos rios e charcos ou lagoas. Quando as pessoas ficam na água, por exemplo para pescar, lavar roupas, tirar água, tomar banho, ou pântanos (machambas de arroz), os bichos entram no corpo e se instalam.

Depois, os bichos se reproduzem dentro do corpo e causam problemas na barriga e na urina. As pessoas infectadas, que estão a defecar ou urinar perto das fontes de água, também deixam bichos que podem infectar outras pessoas.

Os bichos são invisíveis ao olho; então, olhando para a água, não podemos saber se a água está infectada. Geralmente, os bichos vivem só nas águas paradas, como pequenos rios e charcos ou lagoas. A melhora forma para matar os bichos é ferver a água ou tratar com cloro ou certeza.

Como se apanha a bilharziose ?

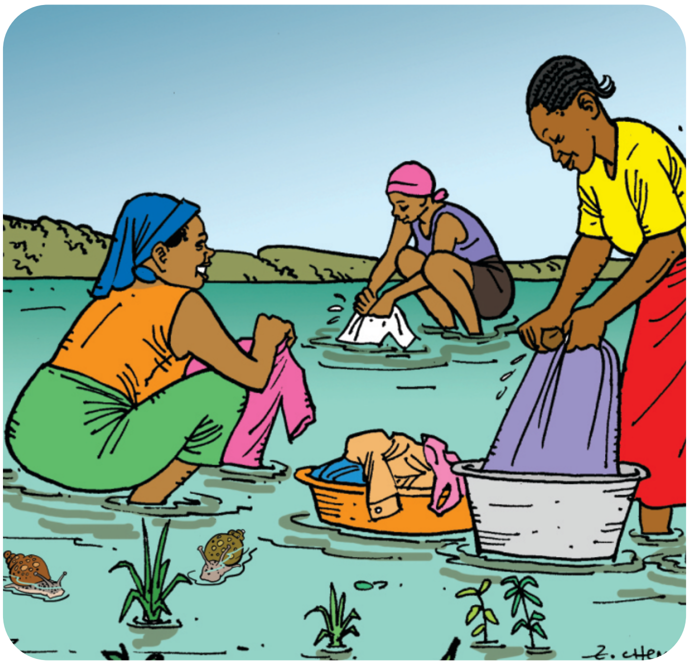

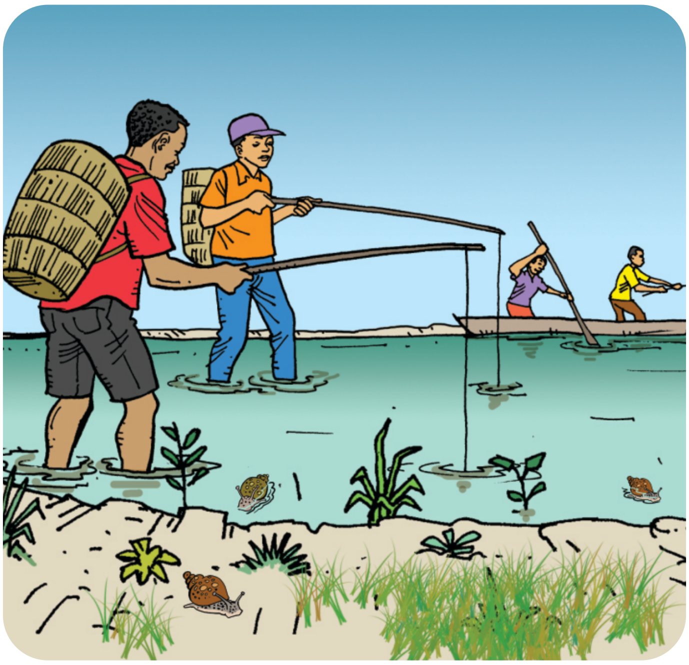

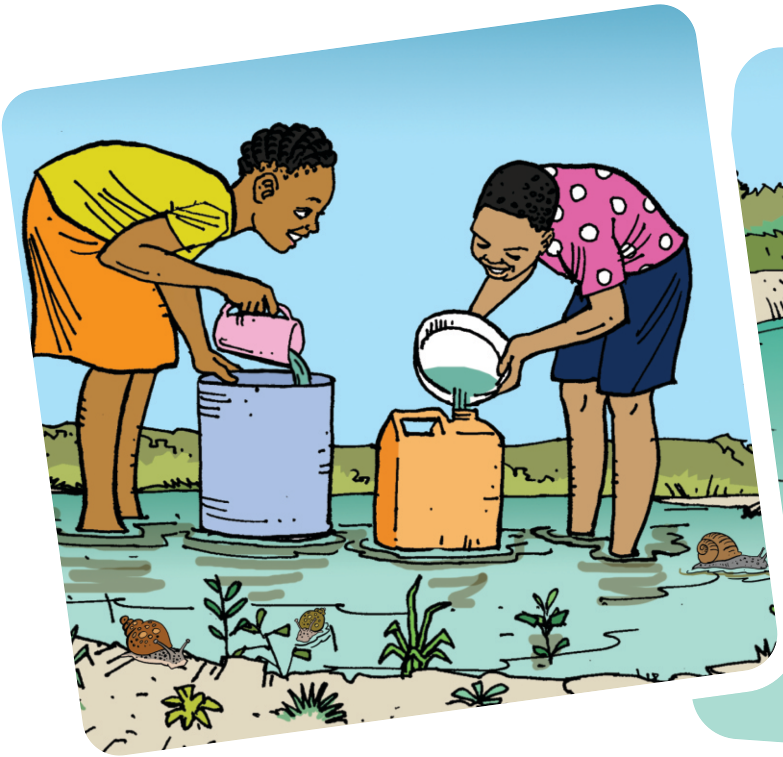

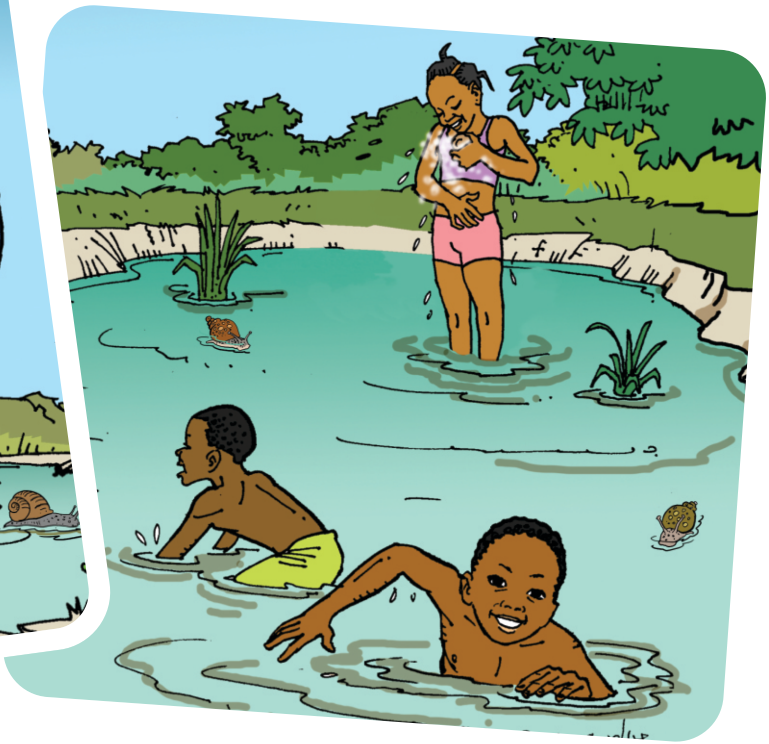

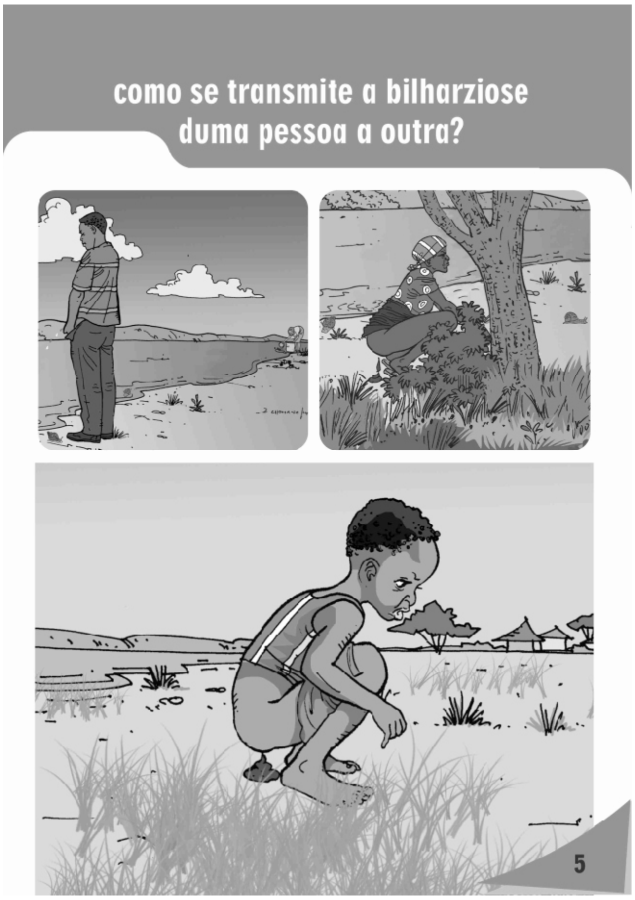

Como se transmite a bilharziose duma pessoa a outra?

Os bichos se reproduzem dentro do corpo e causam problemas na barriga e na urina. As pessoas infectadas, que estão a defecar ou urinar perto das fontes de água, também deixam bichos que podem infectar outras pessoas.

Como se transmite a bilharziose duma pessoa a outra?

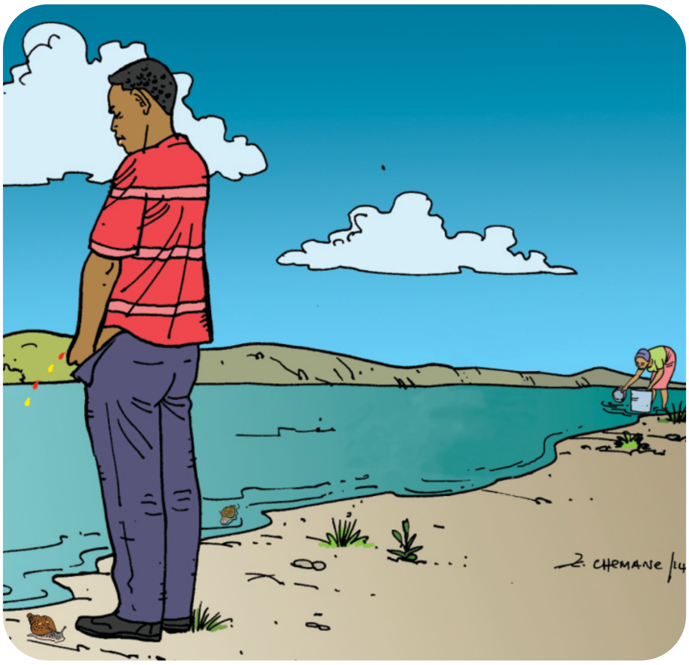

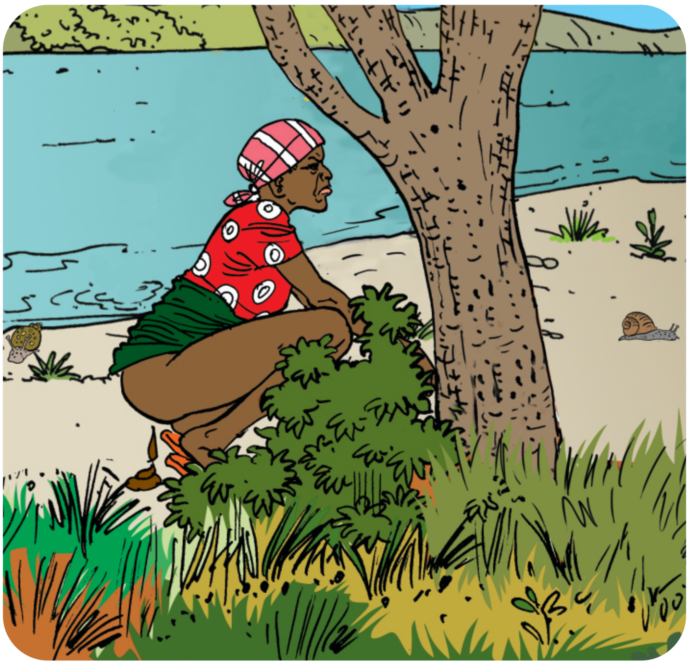

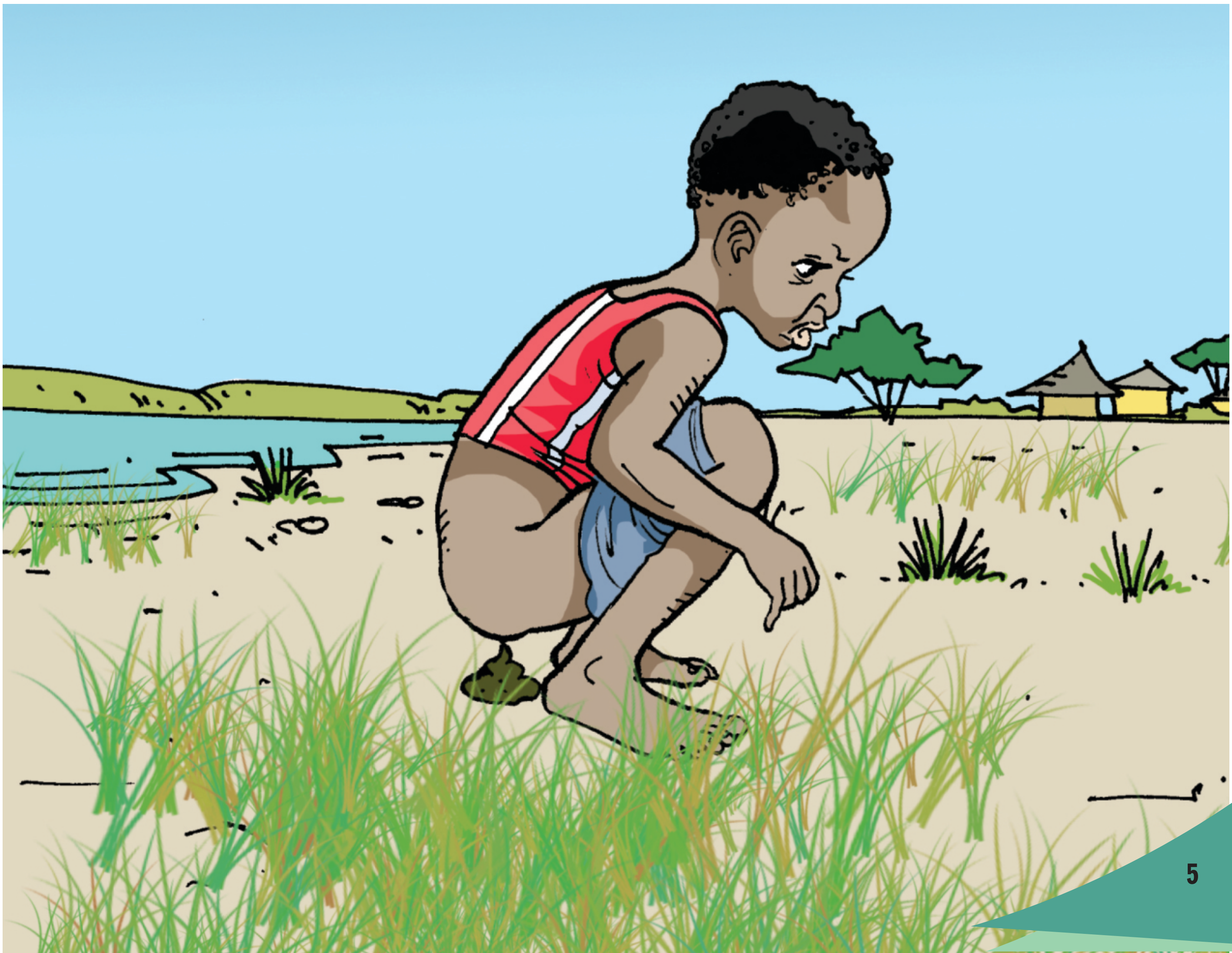

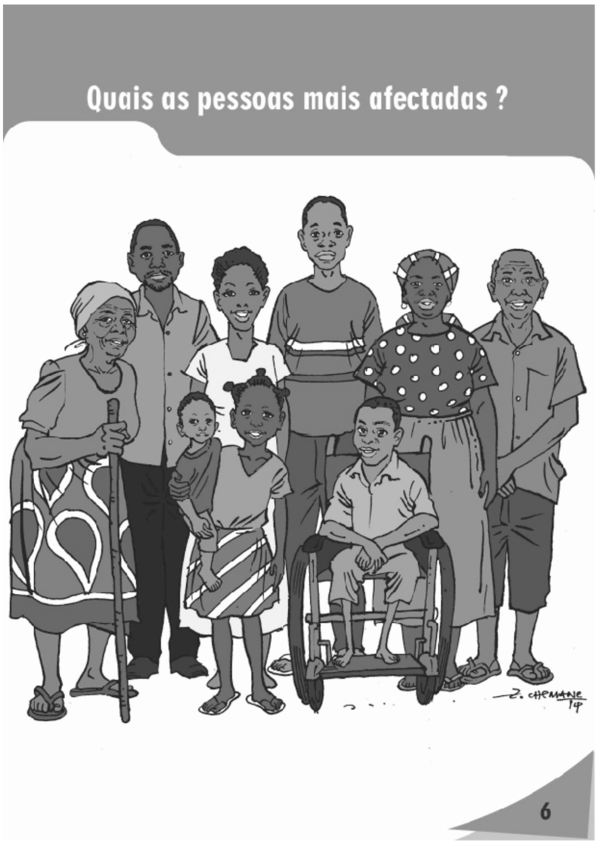

Quais as pessoas mais afectadas?

Todas pessoas que têm contacto com água parada podem apanhar a doença, sejam criança, mulheres, homens, ou idosos. Por exemplo: crianças a tomar banho ou brincar nos charcos; pescadores que ficam com os pês na água; mulheres que lavam roupas com os pés na água; pessoas que trabalham nas machambas com muita água (como machambas de arroz), sem botas, com os pés na água.

Quais as pessoas mais afectadas ?

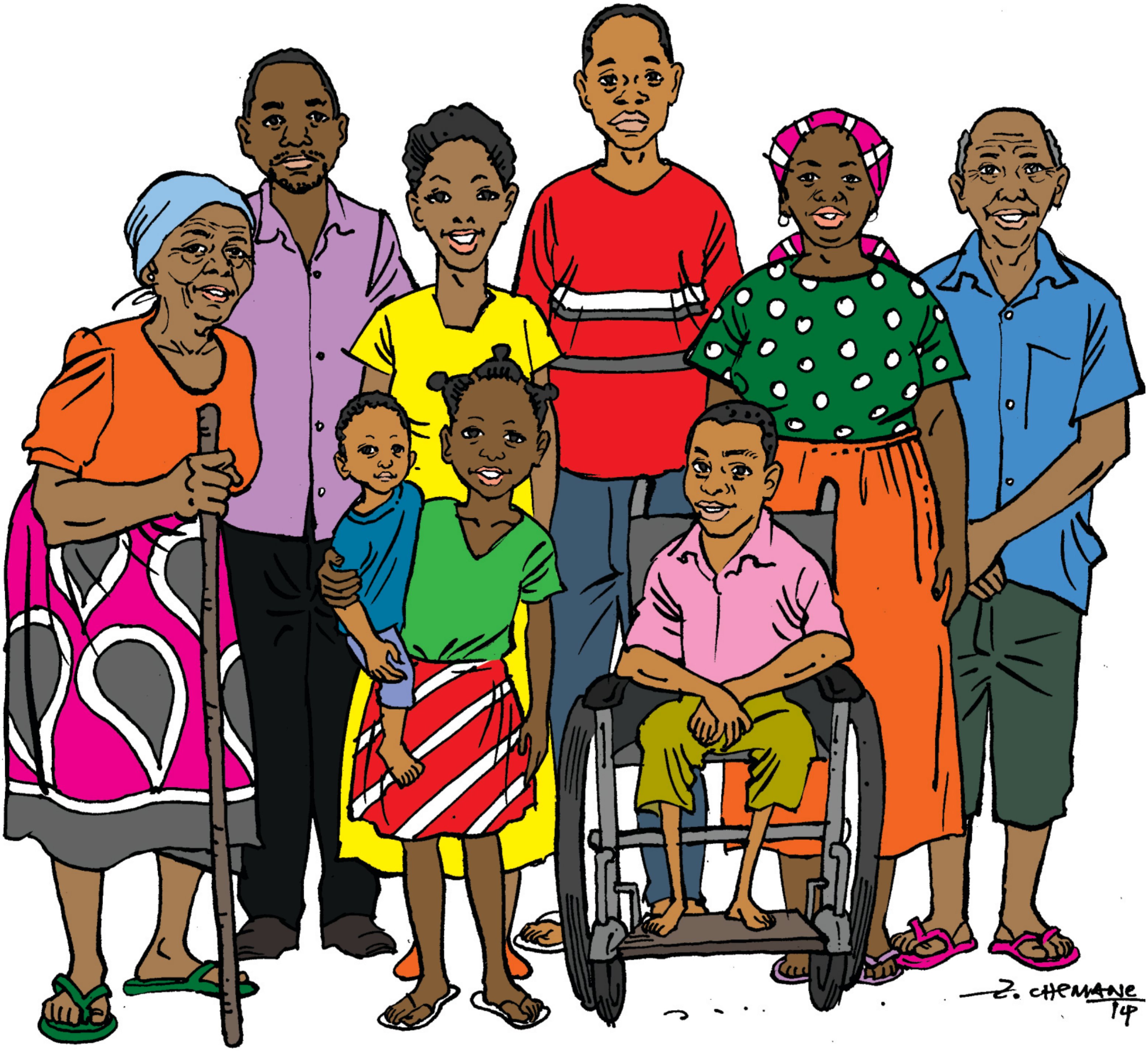

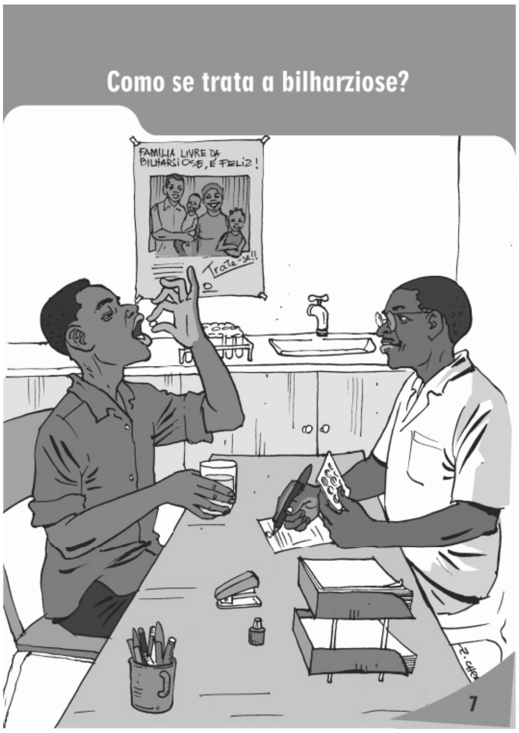

Como se trata a bilharziose?

A bilharziose pode se curar rapidamente, quando for tratada de forma atempada, com uma toma de medicamentos, uma vez só. O medicamento se chama Praziquentel, em forma de comprimidos. O número de comprimidos a ser tomados depende do peso da pessoa doente ou da altura. O Praziquentel mata os bichos que estão dentro do corpo.

Algumas pessoas podem ter uma forma avançada de doença: quando a pessoa tem bichos dentro do corpo desde muito tempo sem ser tratada, e os bichos já atacaram órgãos e criaram problemas; neste caso, mesmo se o Praziquentel mata os bichos, outros problemas de saúde devem ser tratados pelos trabalhadores de saúde.

Depois de tomar o medicamento, a pessoa deve prevenir-se para evitar apanhar a doença novamente.

Como se trata a bilharziose?

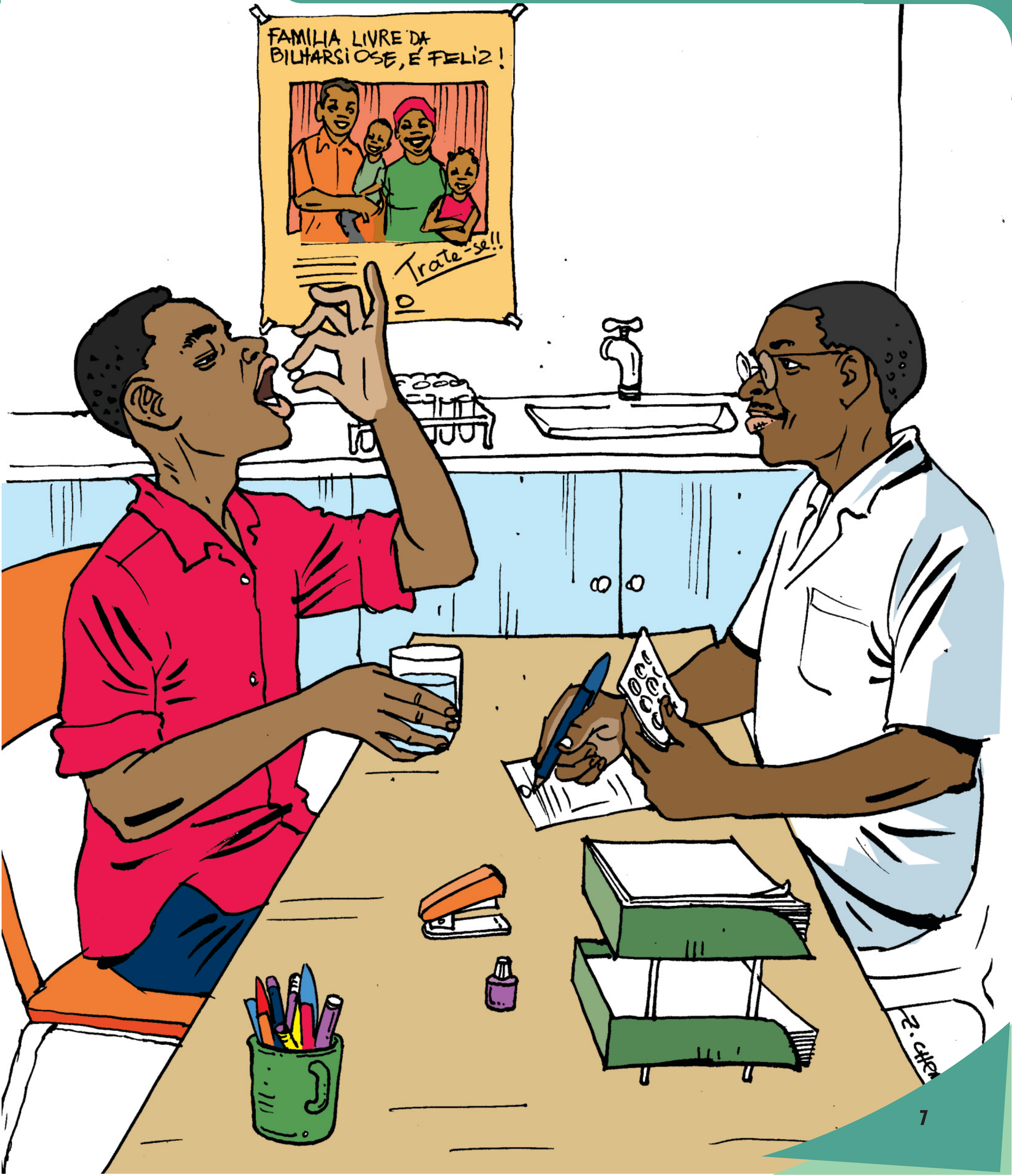

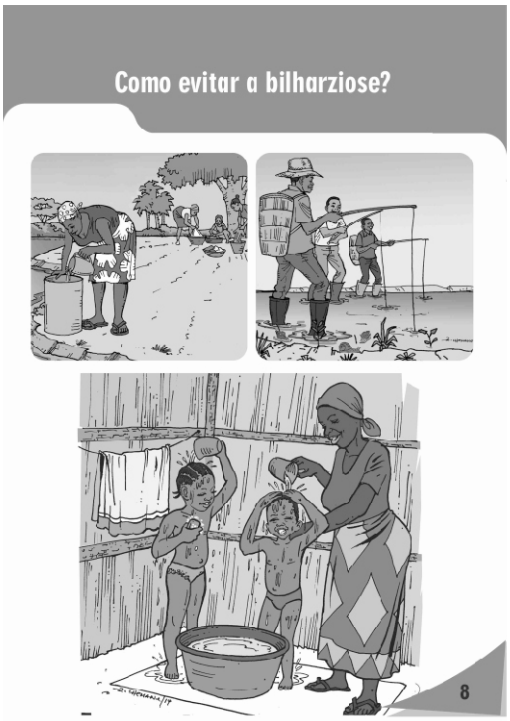

Como evitar a bilharziose?

Há 3 acções importantes para proteger a sua família e a sua comunidade:

1. Não ficar na água parada.

Por exemplo:

- As crianças ou adultos, não devem tomar banho nas águas paradas. É melhor tirar água para tomar banho fora do rio ou do charco.

- Ao tirar água, os pés devem ficar fora do rio ou do charco.

- As mulheres não deveriam lavar as roupas dentro do rio ou da lagoa. E melhor tirar água para lavar roupas num lugar um pouco distante do rio ou do charco.

- Pessoas que trabalham nos pântanos ou que pescam nas lagoas deveriam usar botas, ou parar nas margens para pescar, e não ficar com os pés na água por muito tempo.

- Não ficar nas águas infectadas.

- Ferver água.

- Não defecar ou urinar ao ar livre.

- Tomar medicamento Praziquantel durante campanhas.

Como evitar a bilharziose?

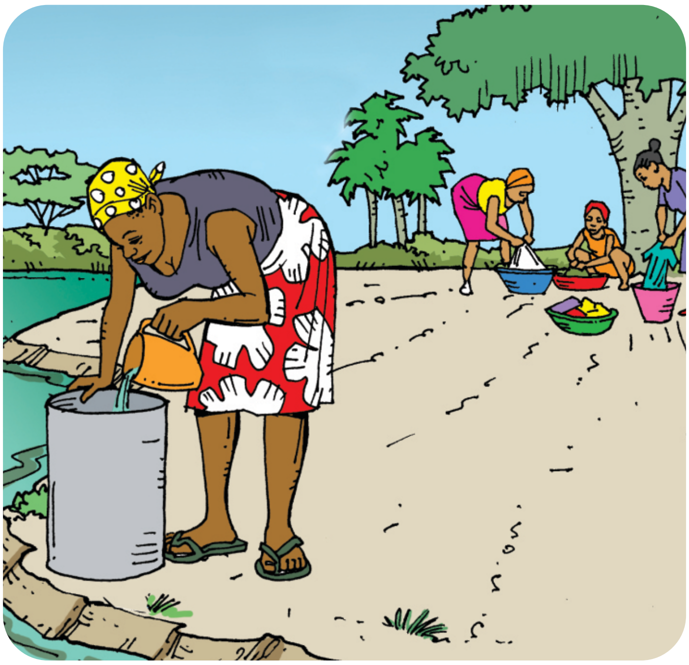

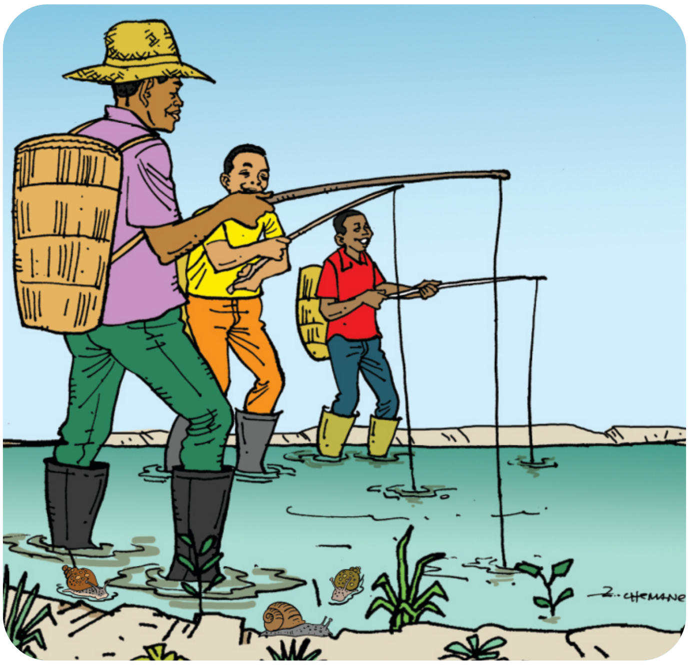

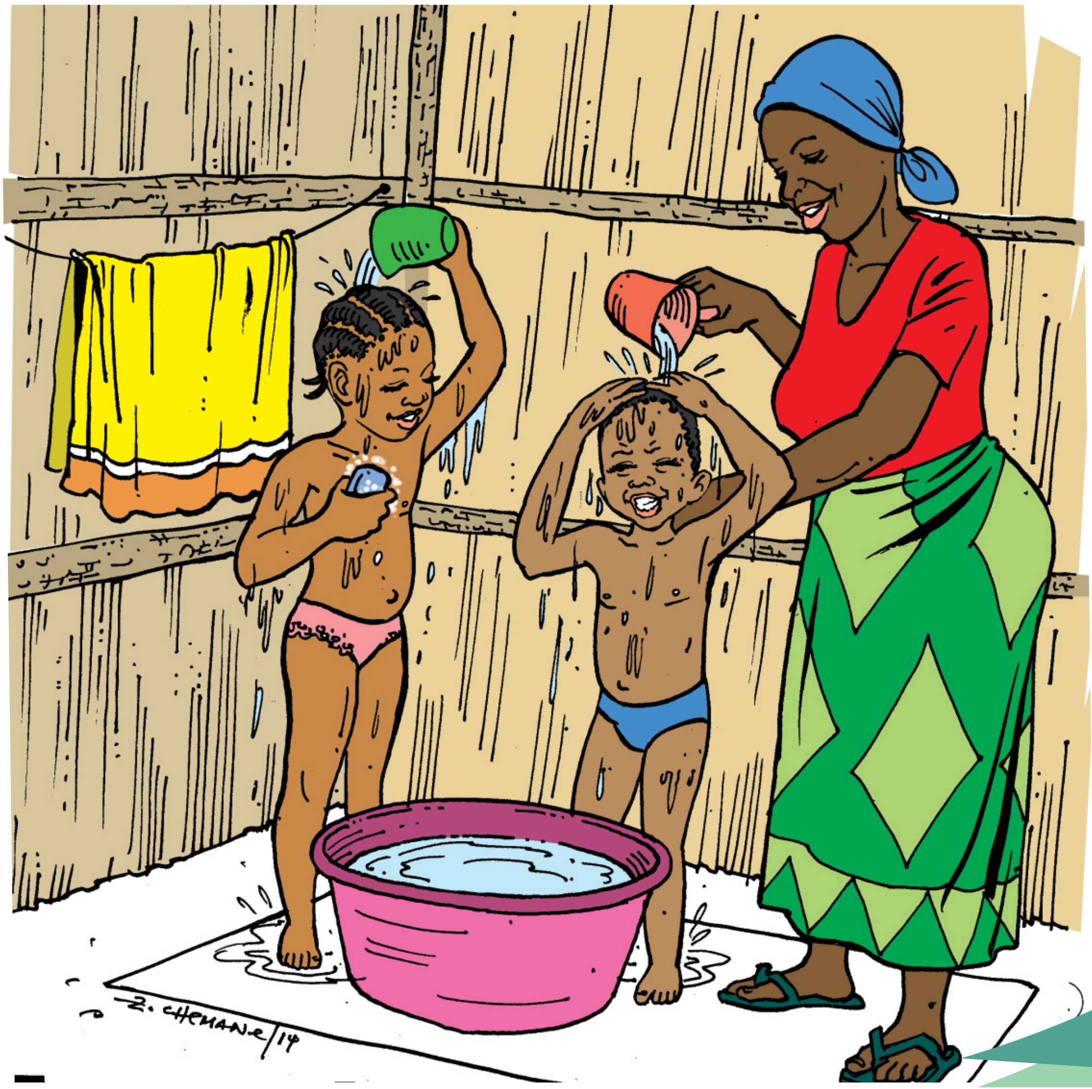

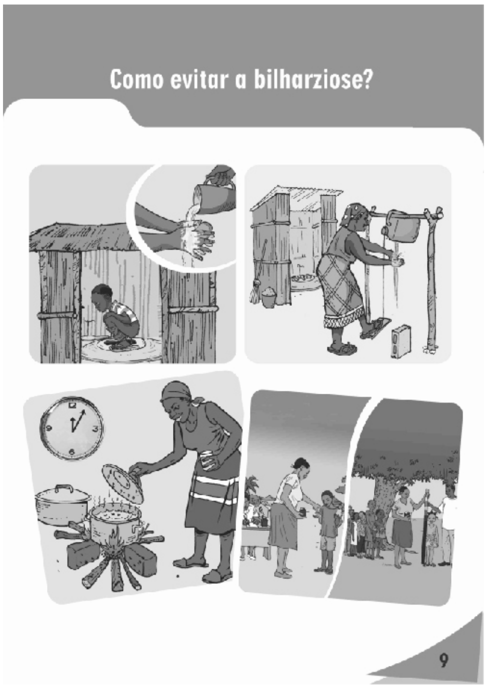

2. Também, deve se cuidar bem da água e da higiene individual e colectiva:

- A água para beber sempre deveria ser tratada com certeza (javel) ou fervida: depois do início da fervura, deixar ferver pelo menos mais 5 minutos.

- Todas pessoas devem usar latrinas para urinar e defecar, e não sujar o ambiente, os rios e lagoas.

3. Participar nas campanhas do Ministério da Saúde que tratam dessa doença:

- Assegurar que os membros da família tomem o medicamento de acordo com a indicação do pessoal da saúde

- Ajudar a mobilizar e informar as pessoas para participarem nas campanhas de distribuição de medicamentos.

Como evitar a bilharziose?

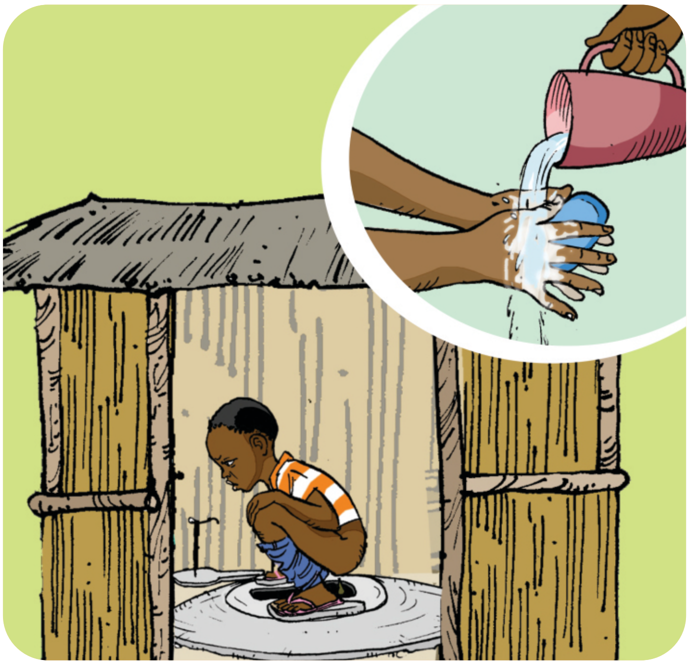

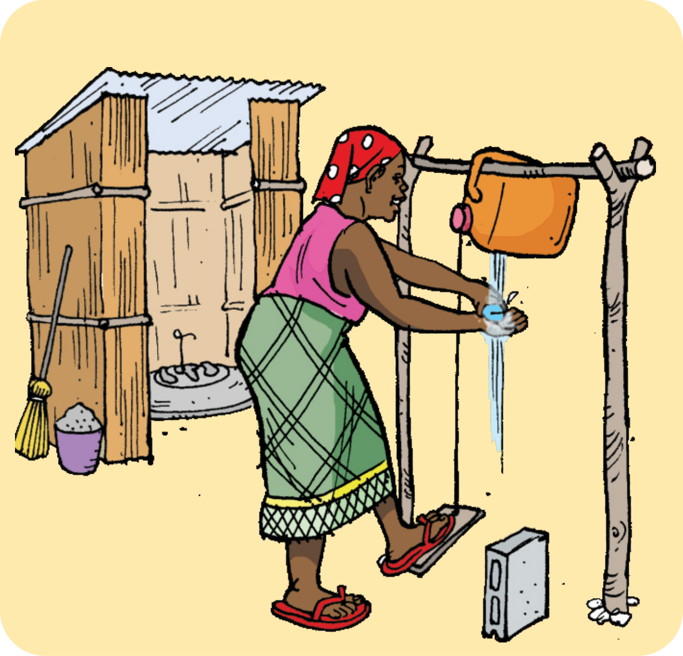

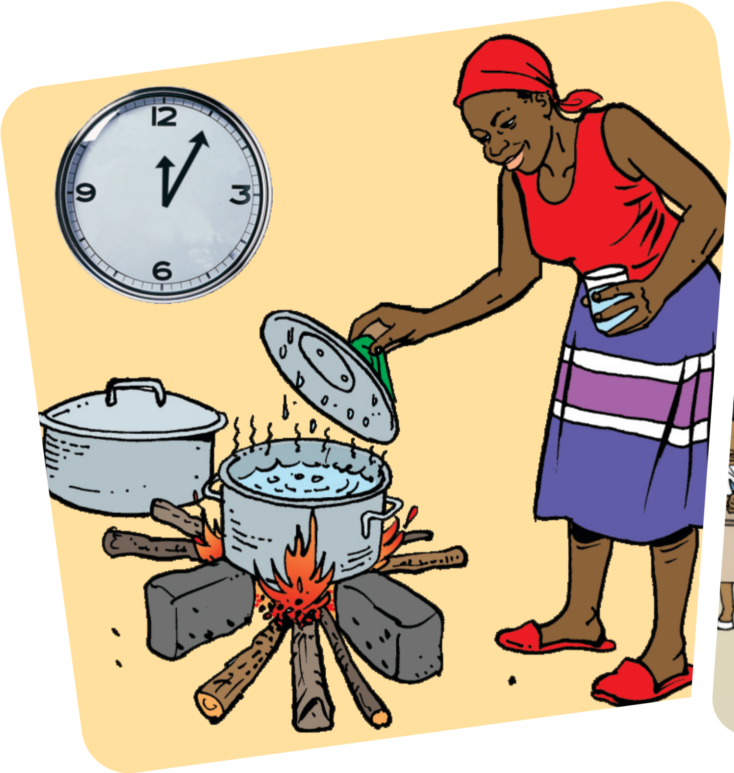

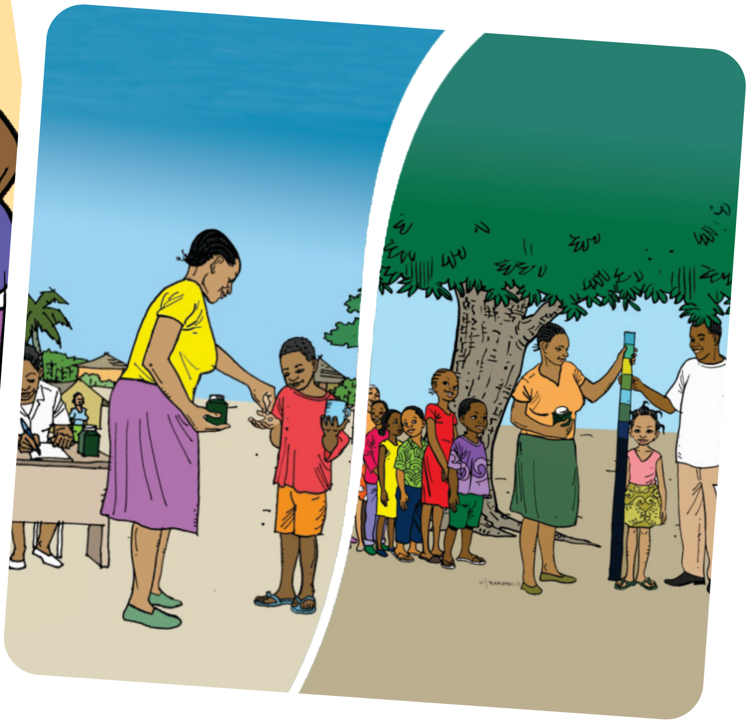

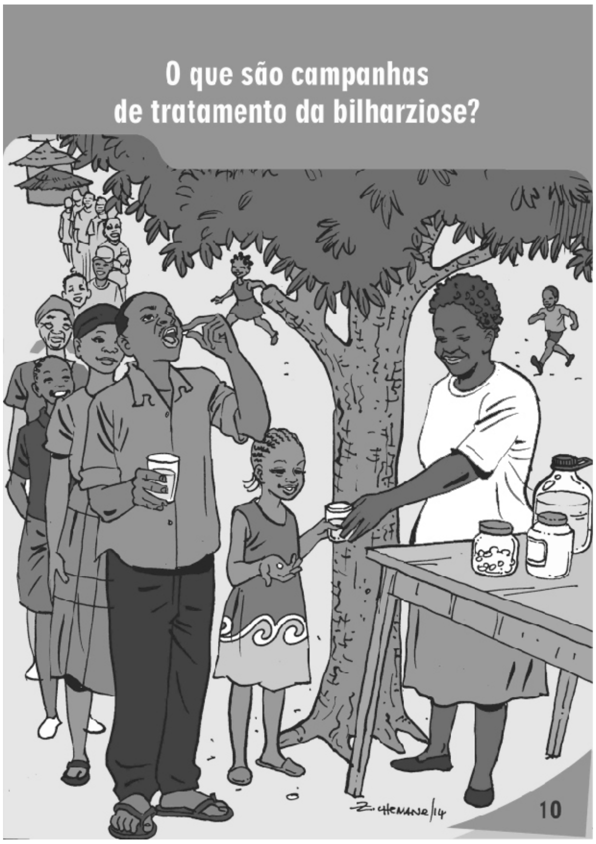

O que são as campanhas de tratamento da bilharziose

A direcção provincial da saúde está a conduzir várias campanhas ao longo do ano para trazer serviços de saúde até a população incluindo campanhas contra a bilharziose.

Durante essas campanhas, o medicamento dado gratuitament se chama Praziquentel e mata bichos que causam a bilharziose.

As campanhas servem para ajudar as comunidades a reduzir a doença, mas as comunidades tem responsabilidade da prevenção da doença. E sempre melhor para o nosso corpo prevenir do que tratar.

O Praziquantel é um medicamento muito seguro que mata bichos que causam a bilharziose. As vezes, algumas pessoas podem sentir desconfortos quando tomarem (como vómitos, dor de barriga, dor de cabeça, comichão na pele); mas estes não duram por muito tempo, apenas algumas horas. Se alguém tiver essas reacções por mais de um dia, deve ir a uma unidade sanitária para ser avaliado pelo médico.

O que são campanhas de tratamento da bilharziose?

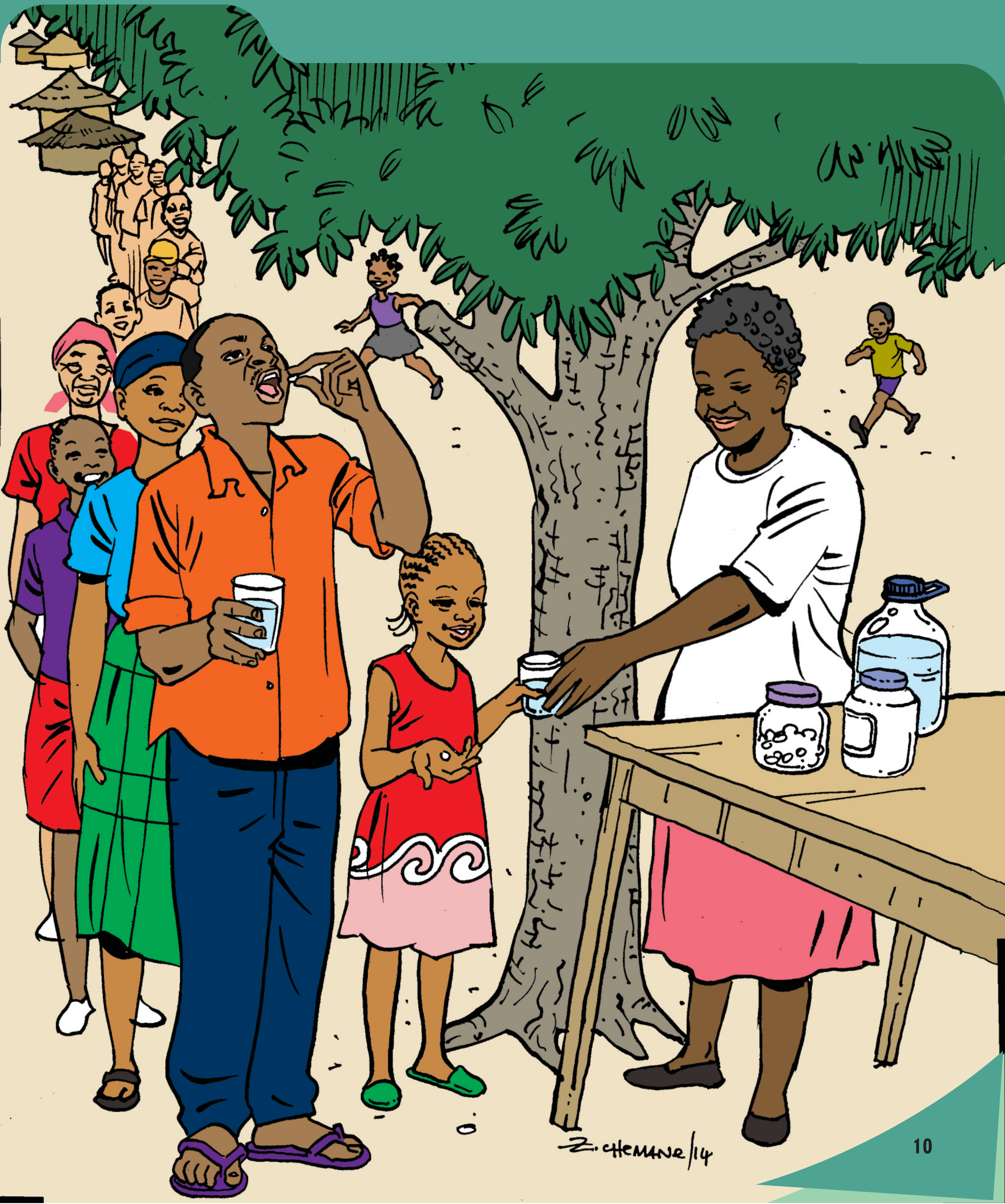

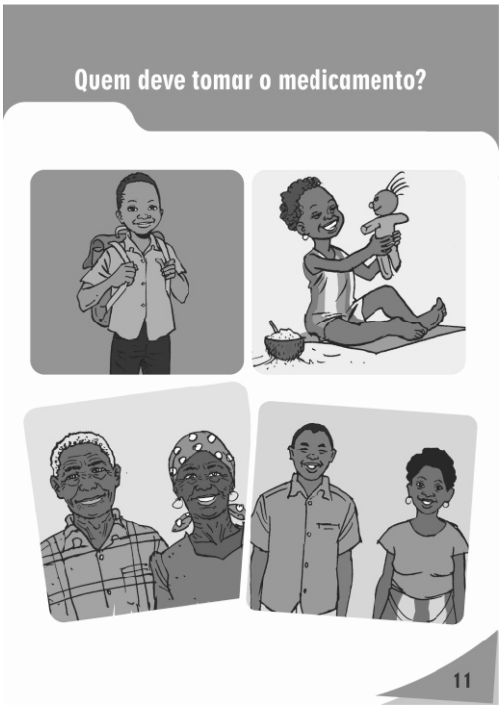

Quem deve tomar o medicamento?

Todas as pessoas a partir de 5 anos de idade podem tomar o medicamento.

Só algumas pessoas não devem tomar: mulheres grávidas, mães que têm bebés recém-nascidos há menos de 7 dias atrás, crianças menores de 5 anos ou abaixo de 90 cm de altura, e doentes graves.

O mesmo medicamento (se chama Praziquentel) é dado a todas pessoas; mas o número de comprimidos a ser tomados depende da altura da pessoa.

- E sempre melhor comer alguma coisa antes de tomar o medicamento.
- Ferver com antecedência um pouco de água e trazer ao ponto de distribuição para tomar o medicamento.
- Não force as crianças a engolir o comprimido. Pode-se esmagar o comprimido e misturá-lo com um pouco de água num copo para ser mais fácil para as crianças tomar.

Quem deve tomar o medicamento?

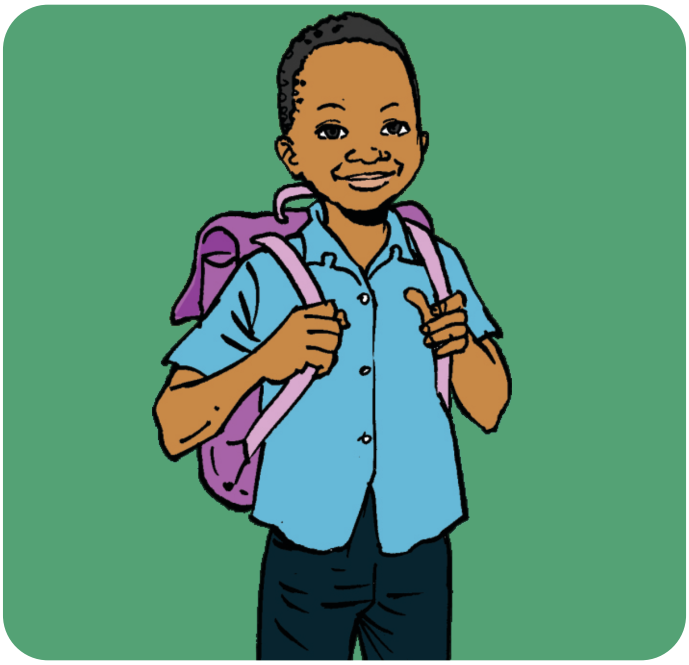
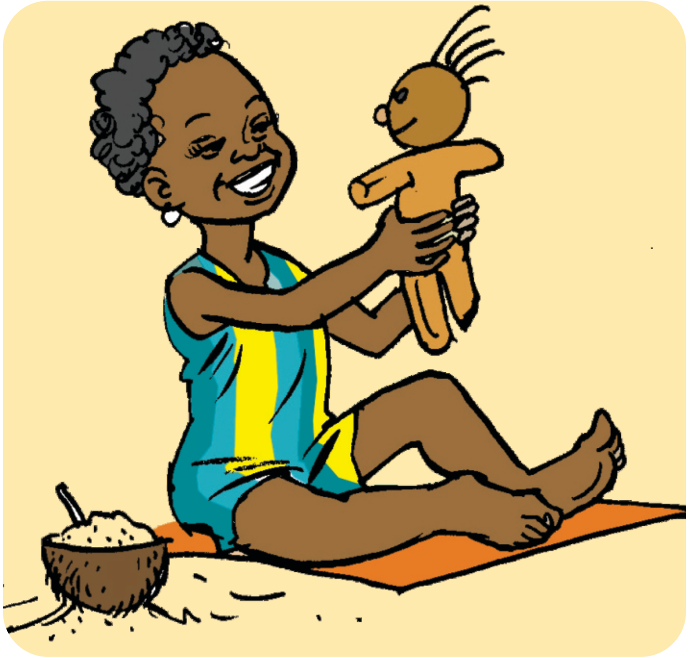
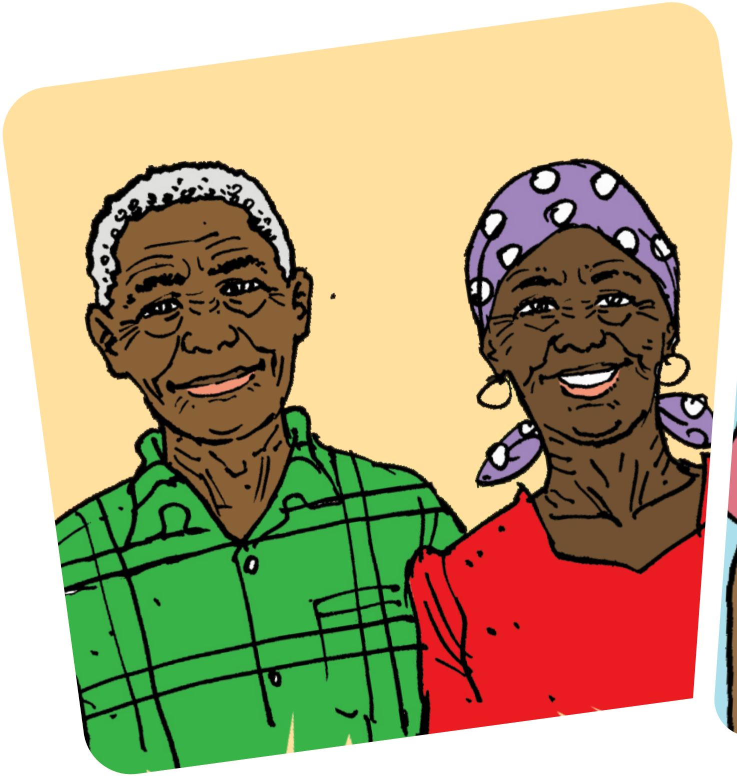
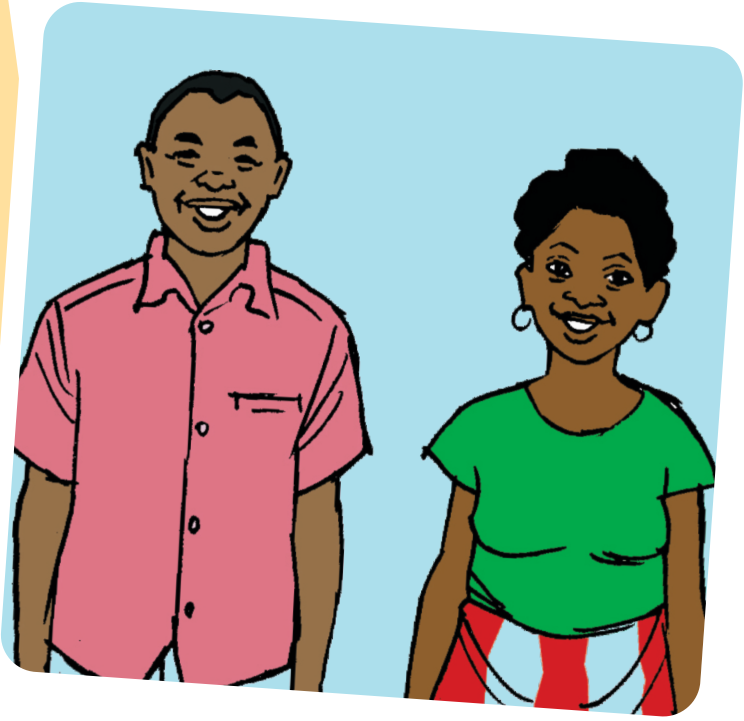

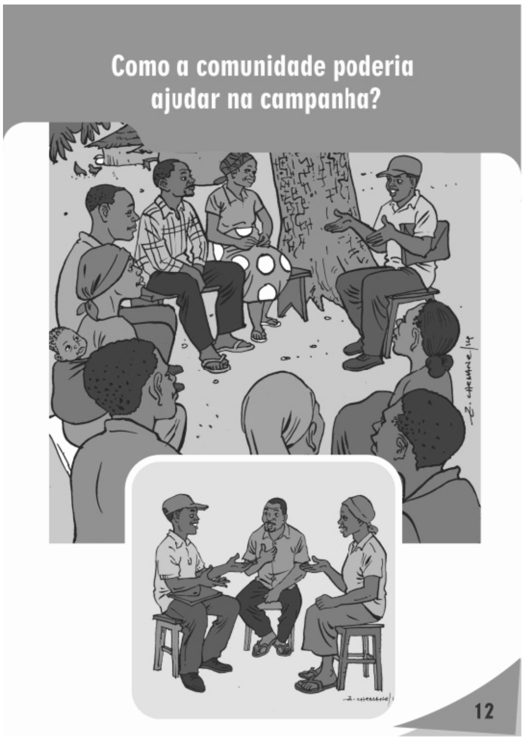

Como a comunidade poderia ajudar na campanha?

Membros da comunidade podem ajudar:

- Assegurar que os membros da família tomem o medicamento de acordo com a indicação do pessoal da saúde;

- Encorajar vizinhos e amigos a participar nas campanhas;

- Ajudar os líderes comunitários e membros do comité de saúde na disseminação da informação sobre o local, a data e hora da distribuição de medicamentos.

- Lembrar a todos que as campanhas servem para ajudar as comunidades a reduzir a doença, mas que as pessoas têm responsabilidade da prevenção da doença.

Como a comunidade poderia ajudar na campanha?

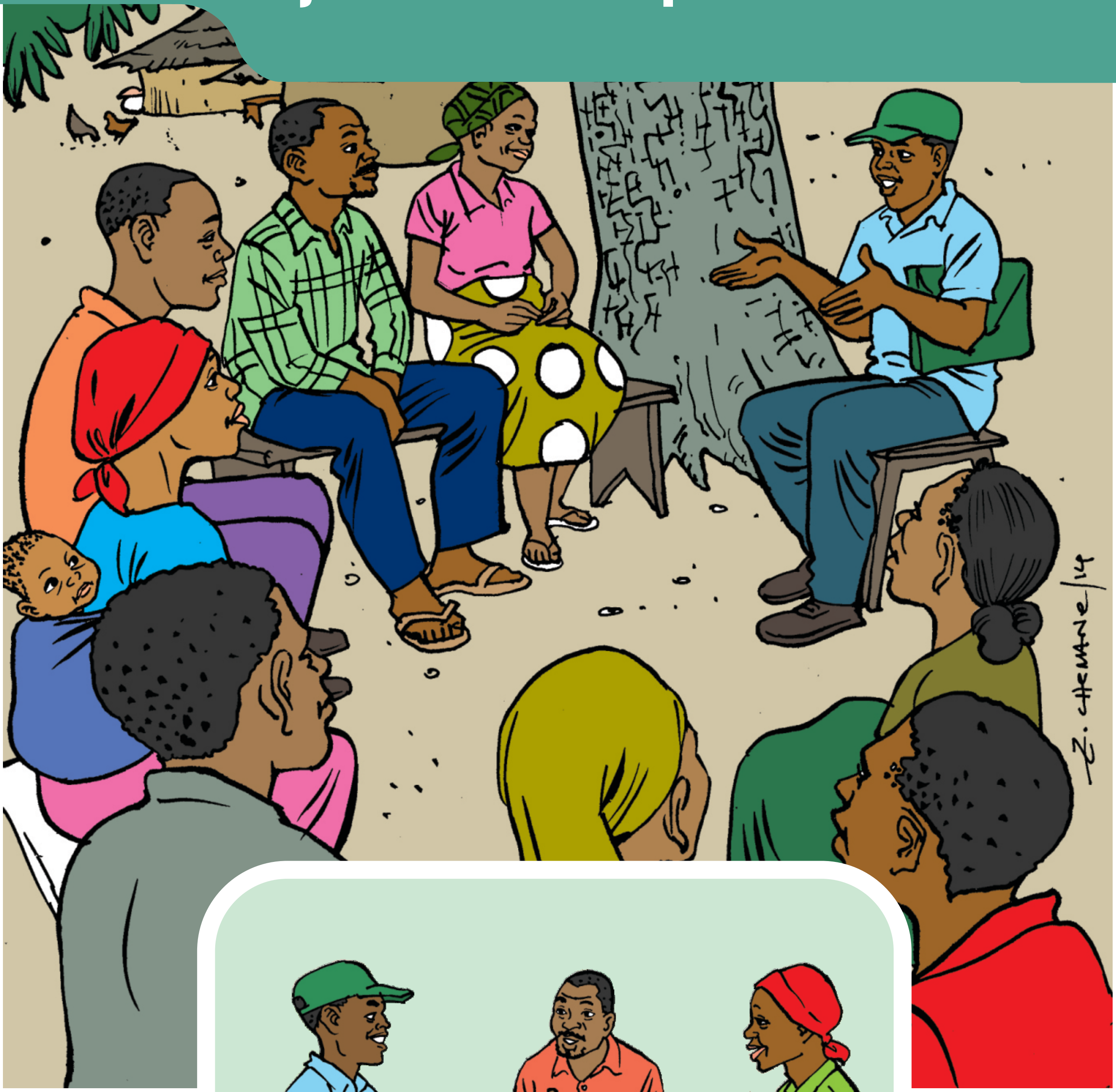

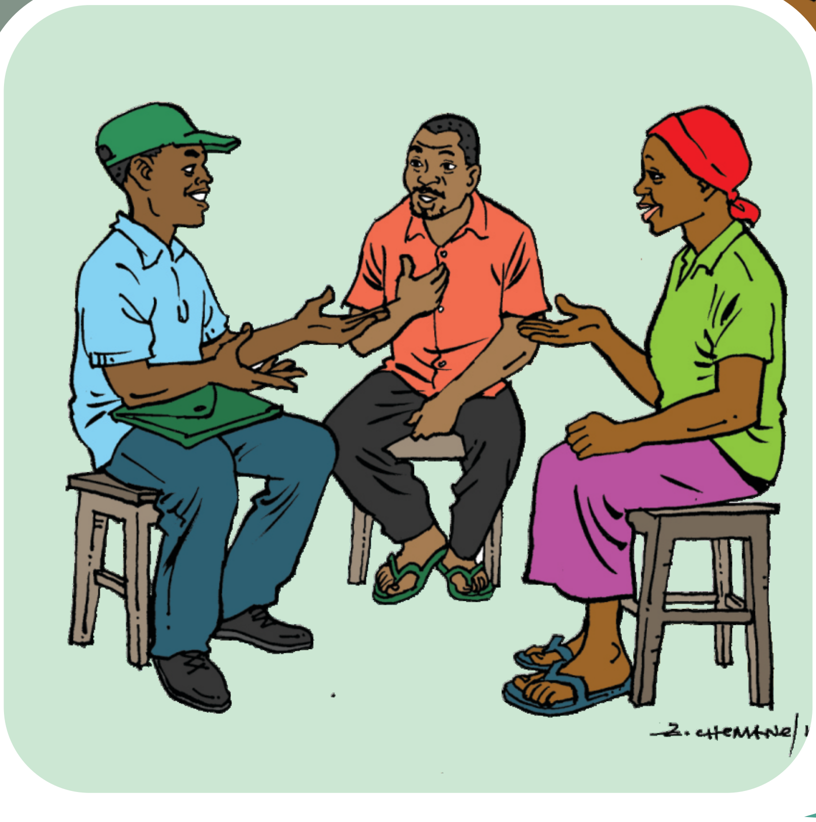

Supplement: S2 Appendix — (PDF) [file pone.0255647.s002.pdf]
