# Supplementary material for: Evaluating the feasibility and acceptability of a community dialogue intervention in the prevention and control of schistosomiasis in Nampula province, Mozambique
Source: PLoS One. 2021 Aug 5;16(8):e0255647. doi: 10.1371/journal.pone.0255647 (PMC8341517; doi:10.1371/journal.pone.0255647)
Supplement: S3 Appendix — (PDF) [file pone.0255647.s003.pdf]

malaria consortium
disease control, better health

Vamos Todos Combater a Bilharziose

GUIÃO DE DIÁLOGO COMUNITÁRIO

Para os facilitadores comunitários

DIRECÇÃO PROVINCIAL DE SAÚDE

DE NAMPULA

Moçambique, Julho 2014

Conteúdo

Ficha de informação sobre o projecto …………………………………………………………….………página 3

Informações básicas sobre a bilharziose …………………………………………………………..…. página 5

Informações básicas sobre as campanhas de tratamento da bilharziose ……..……. página 8

O que e Diálogo Comunitário? ……………………………………………………………………………… página 10

10 Passos para Conduzir um Diálogo Comunitário ………………………………………….…... página 12

Dicas para os facilitadores ……………………………………………………………………………….…. página 14

Exemplos de guião de discussão para diálogos sobre bilharziose ……………………..…. página 15

Instruções para o primeiro diálogo …………………………………………………………..…. página 16

Exemplo de Diálogo - Tema: Conhecer a bilharziose ………………………….….…… página 18

Exemplo de Diálogo - Tema: Acção para evitar a bilharziose: como limitar contacto com água infectada? ………………………………………………………………………………………………. página 22

Exemplo de Diálogo -Tema: Campanha de distribuição de medicamentos .. página 25

Ficha de Monitoria ………………………………………………………………………………………….. página 28

Ficha de planificação e seguimento dos diálogos ………………………………….……….… página 30

Ficha de informação sobre o projecto

Diálogos Comunitários para Prevenção e Controlo das Doenças Tropicais Negligenciadas, em Moçambique

1. Que projecto é esse?

É um projecto de pesquisa operacional que foi concebido pela Malaria Consortium em parceria com a Direcção Provincial de Saúde de Nampula.

2. O que o projecto pretende?

O projecto pretende por meio de várias actividades saber se o modelo de diálogos comunitários é uma ferramenta útil para reforçar a participação das comunidades na melhoria das suas práticas de Saúde para prevenção e controlo das doenças tropicais negligenciadas (DTNs).

3. O que são diálogos comunitários?

Diálogos comunitários acontecem quando um grupo de pessoas da comunidade se reúne para discutir assuntos que preocupam a sua comunidade. É diferente de uma palestra, de uma actividade de sensibilização pois, nestas as pessoas são ouvintes de uma pessoa que vem falar a comunidade. Nos diálogos comunitários as pessoas partilham suas ideias e experiências sobre o tema ou assunto de formas a tomarem melhores decisões sobre os assuntos tratados.

4. O que são doenças tropicais negligenciadas?

São um grupo de doenças transmissíveis que ocorrem principalmente em ambientes de calor e humidade das zonas do clima tropical, como em Moçambique. Afectam principalmente pessoas em áreas remotas. A bilharziose é uma dessas doenças.

5. Qual vai ser a doença mais focalizada?

O enfoque será para a bilharziose que é muito frequente e está muito espalhada na província de Nampula, mas esta doença pode ser facilmente tratada.

6. Quanto tempo o projecto vai durar?

O projecto vai durar cerca de 20 meses e será implementado nas comunidades dos distritos de Eráti, Mecuburi,Murrupula e Mogovolas.

7. Que actividades serão desenvolvidas?

Serão desenvolvidas as seguintes principais actividades:

7.1. Inquérito de base sobre conhecimentos, atitudes e práticas

Um grupo de pesquisadores treinados pela equipe da Malaria Consortium vai realizar entrevistas usando a língua local. Algumas comunidades serão seleccionadas de forma aleatória e um pequeno número de membros da comunidade será entrevistado. A entrevista será feita a um indivíduo que pode responder a perguntas sobre a saúde de sua família e terá uma duração de cerca de 45 minutes.

7.2. Recrutamento e treinamento de facilitadores comunitários

Os facilitadores comunitários é que vão moderar os diálogos comunitários. Estes serão escolhidos dentro da comunidade através dos Comités de Saúde ou dos líderes comunitários ou dos APEs.

7.3. Implementação dos diálogos comunitários (primeira fase)

Os facilitadores comunitários vão conduzir logo após a sua formação, uma ou duas vezes por mês durante o tempo da duração do projecto.

7.4. Avaliação

Serão usadas informações produzidas e registadas pelos facilitadores comunitários, informações produzidas e registadas pelos supervisores. Adicionalmente serão entrevistados facilitadores comunitários e pequeno número de membros da comunidade. Esta avaliação será conduzida pelo Oficial de pesquisas da Malaria Consortium nas comunidades onde os diálogos ocorrerem.

7.5. Implementação dos diálogos comunitários (segunda fase)

Após a avaliação serão feitas algumas revisões nos materiais e técnicas que os facilitadores vão usar de acordo os resultados que serão obtidos. Na segunda fase os facilitadores comunitários vão conduzir os diálogos comunitários seguindo as recomendações da revisão.

7.6. Inquérito final sobre conhecimentos, atitudes e práticas (CAP)

Com a mesma metodologia do inquérito de base, a informação gerada neste servirá para comparar com as informações geradas do inquérito base de forma a avaliar se os diálogos comunitários produziram melhorias nas práticas sobre saúde na comunidade.

8. Que benefícios o projecto pode trazer para as comunidades?

Este projecto vai despertar as pessoas em relação as DNT que são frequentes e alastradas nas nossas comunidades. As pessoas das nossas comunidades terão mais conhecimentos sobre a bilharziose, suas causas, sintomas, medidas preventivas e o que fazer individual e colectivamente para acabar com esta e outras doenças nas nossas comunidades.

Contactos: **845810401-Valdimar Antonio (Malaria Consortium)**

845835570 - Dr. Ercílio Salomão (Direcção Provincial de Saúde-Nampula)

Informações básicas sobre a bilharziose

O QUE É A BILHARZIOSE?

A bilharziose é uma doença muito comum nas nossas comunidades, onde pessoas usam a água dos rios, lagoas ou charcos. Muitas pessoas estão infectadas com a doença: adultos, crianças, idosos. Mas muitas pessoas não sabem que têm bilharziose, porque a doença pode ser confundida com outros problemas. Esta doença pode ficar no corpo durante muitos anos, sem as pessoas descobrirem.

A bilharziose pode ser muito grave a longo prazo, incluindo a infertilidade, e até matar, se não for tratada. Mas existem soluções para evitar e tratar a bilharziose.

As pessoas podem evitar e tratar a doença através de acções discutidas e tomadas juntamente.

Nas comunidades, há pessoas que tem informações ou experiências desta doença que serão muito úteis para todos percebermos melhor o que é esta doença, como se reconhece, como se trata e como se evita.

É importante que os membros da comunidade partilhem informações e experiências sobre a doença para juntos resolver este problema de bilharziose, através de soluções comuns e factíveis, para melhorar a saúde da comunidade.

Como saber se tem a doença?

A doença afecta a bexiga, e as vezes a barriga, por causa de bichos dentro do corpo: o primeiro sinal de bilharziose é ter sangue na urina e dor ao urinar; depois, outros sinais são: a barriga pode ficar muito grande (barriga de água), ou com dores; a pessoa doente pode ter diarreia.

Muitas pessoas podem confundir esta doença com outros problemas, particularmente doenças sexuais.

Pessoas que tem esse tipo de sinais no corpo ou ao urinar deveriam ir a Unidade Sanitária, para fazer um teste simples de urina que vai descobrir se a pessoa tem a bilharziose ou outra doença.

Como se apanha a doença?

A doença se apanha através de pequenos bichos que vivem dentro de caracol nas águas paradas, como pequenos rios e charcos ou lagoas. Quando as pessoas ficam na água, por

exemplo para pescar, lavar roupas, tirar água, tomar banho, ou pântanos (machambas de arroz), os bichos entram no corpo e se instalam.

Depois, os bichos se reproduzem dentro do corpo e causam problemas na barriga e na urina. As pessoas infectadas, que estão a defecar ou urinar perto das fontes de água, também deixam bichos que podem infectar outras pessoas.

Quais as pessoas mais afectadas?

Todas pessoas que têm contacto com água parada podem apanhar a doença, sejam criança, mulheres, homens, ou idosos. Por exemplo: crianças a tomar banho ou brincar nos charcos; pescadores que ficam com os pés na água por muito tempo; mulheres que lavam roupas com os pés na água; pessoas que trabalham nas machambas com muita água (como machambas de arroz), sem botas, com os pés na água.

Como se trata a bilharziose?

A bilharziose pode se curar rapidamente, quando for tratada de forma atempada, com uma toma de medicamentos, uma vez só. O medicamento se chama Praziquantel, em forma de comprimidos. O número de comprimidos a ser tomados depende do peso da pessoa doente ou da altura. O Praziquantel mata os bichos que estão dentro do corpo.
Algumas pessoas podem ter uma forma avançada de doença: quando a pessoa tem bichos dentro do corpo desde muito tempo sem ser tratada, e os bichos já atacaram órgãos e criaram problemas; neste caso, mesmo se o Praziquantel mata os bichos, outros problemas de saúde devem ser tratados pelos trabalhadores de saúde.

Como evitar a doença?

Há 3 acções importantes para proteger a sua família e a sua comunidade:

1. **Não ficar na água parada durante muito tempo.**

Por exemplo:

- As crianças ou adultos, não devem tomar banho nas águas paradas. É melhor tirar água para tomar banho fora do rio ou do charco.
- Ao tirar água, os pés devem ficar fora do rio ou do charco.
- As mulheres não deveriam lavar as roupas dentro do rio ou da lagoa. E melhor tirar água para lavar roupas num lugar um pouco distante do rio ou do charco.
- Pessoas que trabalham nos pântanos ou que pescam nas lagoas deveriam usar botas, ou parar nas margens para pescar, e não ficar com os pés na água por muito tempo.

2. **Também, deve se cuidar bem da água e da higiene individual e colectiva:**

- A água para beber sempre deveria ser tratada com certeza (javel) ou fervida: depois do início da fervura, deixar ferver pelo menos mais 5 minutos.

- Todas pessoas devem usar latrinas para urinar e defecar, e não sujar o ambiente, os rios e lagoas.

3. Participar nas campanhas do Ministério da Saúde que tratam dessa doença:

- Assegurar que todos os membros da sua família tomam o medicamento preventivo.
- Ajudar a mobilizar e informar as pessoas para participarem nas campanhas de distribuição de medicamentos

Como podemos saber se a água está infectada?

Os bichos são invisíveis ao olho; então, olhando para a água, não podemos saber se a água está infectada. Geralmente, os bichos vivem só nas águas paradas, como pequenos rios e charcos ou lagoas. A melhora forma para matar os bichos é ferver a água ou tratar com cloro ou certeza.

Informações básicas sobre as campanhas de tratamento da bilharziose

Quem está conduzir as campanhas de tratamento da bilharziose?

É a Direcção Provincial da Saúde de Nampula.

Quando e onde é que essas campanhas acontecem?

As campanhas acontecem geralmente duas vezes ao longo do ano nas nossas comunidades ou nos nossos bairros para trazer serviços de saúde até a população.

Quais medicamentos são distribuídos durante essas campanhas?

Durante essas campanhas, dois tipos de medicamentos são dados a todas pessoas, gratuitamente, em forma de comprimidos: um medicamento que se chama Praziquantel e que mata bichos que causam a bilharziose; um outro medicamento que se chama Albendazol que mata outros vermes que ficam no nosso corpo e que causam outras doenças.

O que o medicamento faz no corpo exactamente?

O Praziquantel é um medicamento muito seguro que mata os bichos que estão dentro do corpo e que provocam a bilharziose.

As vezes, algumas pessoas podem sentir desconfortos quando tomarem (como vómitos, dor de barriga, dor de cabeça, comichão na pele); mas estes não duram por muito tempo, apenas algumas horas. Se alguém tiver essas reacções por mais de um dia, deve ir a uma unidade sanitária para ser avaliado pelo médico.

Quem deve tomar o medicamento?

Todas as pessoas a partir de 5 anos de idade devem tomar o medicamento.

Só algumas pessoas não devem tomar: mulheres grávidas, mães que têm bebés recém-nascidos há menos de 7 dias atrás, crianças menores de 5 anos ou abaixo de 90 cm de altura, e doentes graves.

Existe dosagem diferente para várias pessoas?

O mesmo medicamento (se chama Praziquantel) é dado a todas pessoas; mas o número de comprimidos a ser tomados depende da altura da pessoa.

A bilharziose pode se curar rapidamente, quando for tratada de forma atempada, com uma toma de medicamentos, uma vez só. Quando uma pessoa fica muito tempo sem tratar a bilharziose, depois da toma dos medicamentos o Praziquantel pode matar os bichos, mas outros problemas de saúde podem existir no corpo da pessoa. Nestes casos, a pessoa deve ir a unidade sanitária para ser tratada.

O que deve fazer ou trazer para tomar o medicamento?

E sempre melhor comer alguma coisa antes de tomar o medicamento.

Ferver com antecedência um pouco de água e trazer ao ponto de distribuição para tomar o medicamento.

Não force as crianças a engolir o comprimido. Pode-se esmagar o comprimido e misturá-lo com um pouco de água num copo para ser mais fácil para as crianças tomar.

Porque deve tomar o medicamento mesmo se não me sinto doente?

Muitas pessoas estão infectadas e não sabem. Mesmo assim, as pessoas que não se sentem doentes devem tomar o medicamento para matar os bichos da bilharziose no corpo e não deixar bichos (ao defecar ou urinar) que podem infectar outras pessoas.

Como a comunidade poderia ajudar na campanha?

Assegurar que todos os membros da família tomam o medicamento;

Encorajar vizinhos e amigos a participar nas campanhas;

Ajudar os líderes comunitários e membros do comité de saúde na disseminação da informação sobre o local, a data e hora da distribuição de medicamentos;

Diferença entre diálogo e palestra

Um diálogo comunitário acontece quando os membros da comunidade se reúnem para discutir questões de seu interesse. Este Diálogo Comunitário já está a acontecer na maioria das comunidades para discutir vários temas durante as reuniões da comunidade.

"O diálogo comunitário" é diferente da "sensibilização da comunidade" ou palestra. Numa palestra, usualmente, uma pessoa fala para a comunidade sobre um tema. Num diálogo, todos os participantes podem partilhar informação e experiências sobre o tema. Durante o Diálogo, os participantes tem que discutir em conjunto o que a comunidade deveria fazer para resolver os problemas de saúde e assim melhorar a situação. No final do diálogo, os membros da comunidade devem chegar a acordo sobre acções a serem tomadas e como essas acções serão implementadas.

O que e Diálogo Comunitário?

O objectivo é estimular a discussão participativa e a tomada de decisões sobre as acções saudáveis simples que as famílias e comunidades podem tomar para reduzir a doença através do tratamento e das medidas preventivas.

Cada diálogo serve para:

- **Partilhar conhecimento sobre como reduzir** a bilharziose e promover uma comunidade saudável;
- **Identificar acções** factíveis para tratar e prevenir a bilharziose nas famílias e nas comunidades.
- **Concordar e implementar** acções individuais e colectivas para efectivamente reduzir a doença.

Quem pode conduzir um Diálogo Comunitário?

Facilitadores Comunitários que receberam um treino sobre diálogos comunitários sobre a bilharziose podem organizar e conduzir diálogos: sejam religiosos, curandeiros, membros do Comité de Saúde, ou outras pessoas influentes.

Quem deve participar nos diálogos?

- Representantes das famílias que vivem na comunidade, que são responsáveis pelo bem-estar da sua família.
- Os líderes comunitários, líderes religiosos e membros das organizações locais: eles vão ajudar as pessoas a fazer a ligação com a tradição e provérbios locais, que complementam os comportamentos saudáveis.
- Activistas de saúde e APEs que poderão responder as questões sobre saúde.

Onde pode se realizar um diálogo comunitário?

Em qualquer lugar onde os participantes se sentem bem para discutir abertamente, podem se reunir: na escola, nos centros comunitários; nos bairros, nos lugares onde rezam, no posto de saúde.

Quantas vezes devem ser realizados os diálogos comunitários?

Pelo menos um diálogos por mês, durante 6 meses, ou sempre que os membros ou líderes da comunidade sentirem que há necessidade de discutir um problema, podem se encontrar.

Quais temas deveriam ser discutidos?

Não será possível de abordar todas perguntas sobre a bilharziose num único diálogo. E aconselhável de planificar um conjunto de diálogos, ao longo de 6 meses, para discutir da doença, com foco nas acções recomendadas para evita-la.

Exemplo de temas a ser discutidos:

Diálogo 1: Conhecer a bilharziose: como saber se tem a doença? Como se trata? Como se apanha? Como se evita?

Diálogo 2: Acção para evitar a bilharziose: como limitar contacto com água infectada?

Diálogo 3: Acção para evitar a bilharziose: manuseio seguro de água que pode ser infectada.

Diálogo 4: Acção para evitar a bilharziose: boas práticas de higiene

Diálogo 5: Antes duma campanha de distribuição de medicamentos: importância e procedimentos da campanha

Diálogo 6: Depois duma campanha de distribuição de medicamentos: Vantagens e desafios das campanhas, porque prevenir e evitar a reinfecção.

10 Passos para Conduzir um Diálogo Comunitário

Antes do diálogo:

1. Conhecer o seu Guião de Diálogo Comunitário, e as imagens que podem ser usadas para estimular a discussão.

2. Falar com líderes, membros influentes e membros do comité de saúde que podem ajudar no diálogo.

3. **Marcar a data, hora e tema**, com todos os participantes.

No dia do diálogo:

4. **Introdução**

Depois dos cumprimentos dos participantes, apresentar o tema e o objectivo do diálogo. Poderia usar uma canção ou história de interesse de todos para abrir o diálogo duma maneira motivadora.

5. Explorar o tema

Estimule um debate, deixando as pessoas a partilhar os seus conhecimentos e experiências pessoais sobre a bilharziose, usando exemplos de perguntas no seu Guião de Dialogo, e mostrando imagens do Álbum.

Pedir a algumas pessoas para descreverem a imagem e mostra-la ao grupo. Depois, pedir aos participantes para debaterem.

Antes de passar para a imagem seguinte confirme se foram discutidas as informações básicas (texto atrás das imagens do seu Álbum).
Perguntar se todos entendem as frases, e clarificar se for necessário.

6. Identificar acções

Em cada diálogo, enfocar a discussão sobre uma ou duas acções concretas para reduzir a doença na comunidade.

Usando a Ficha de Planificação e seguimento dos diálogos, pedir aos participantes para reportar sobre acções que eles concordaram em fazer no último diálogo, e discutir como ultrapassar as dificuldades. Depois, pedir aos participantes que escolham juntos mais uma ou duas acções factíveis que devem ser feitas.

7. **Tomar decisões**

Os participantes devem concordar com os passos: Quem é que vai fazer o quê? Quando? Como?

Anotar na Ficha de planificação e seguimento dos diálogos as decisões tomadas, responsabilidades e ponto de situação se for o caso.

8. Resumir as informações usando o Álbum Seriado. Lembrar a todos sobre as decisões tomadas.

9. Agradecer aos participantes por terem partilhado as suas opiniões e marcar o próximo encontro.

No fim do diálogo:

10. Preencher a Ficha de Monitoria do diálogo.

"Durante o Diálogo, os participantes tem que discutir em conjunto o que a comunidade deveria fazer para resolver os problemas de saúde e assim melhorar a situação"

Dicas para os facilitadores

- Cada diálogo pode durar até duas horas, se durar mais de duas horas os membros da comunidade podem deixar de prestar atenção.
- Usa imagens para mostrar e estimular o debate. Organize o grupo de forma que fiquem todos o melhor instalado possível e que consigam ver as imagens. Se for possível convide-os a sentarem-se em círculo.
- Combine com o grupo para todos falarem alto de forma a se fazerem ouvir. Lembre a todos que devem ouvir com respeito.
- Comunique-se numa língua que todos percebam, use de preferência a língua local.
- Ajude todos os participantes a sentirem-se à vontade para expressarem as suas opiniões e fazerem perguntas. Assegurar que ninguém fale demais.
- Permaneça neutro, respeite as opiniões dos membros da comunidade.

  Encoraje todas as pessoas na reunião a participarem. Se algumas pessoas não falarem, dê tempo para que elas também falem, mas não os force.

FAZER PERGUNTAS:

• Dar tempo às pessoas para responder a cada pergunta. Se ninguém responde, faça a mesma pergunta usando outras palavras.

• Quando alguém dá uma resposta que é errada, continue perguntando se alguém tem uma resposta diferente. Quando for dada a resposta correcta, repita-a, para que todos possam retê-la.

Problemas que podem acontecer e como resolver:

- Se algumas questões não puderem ser respondidas durante o diálogo, os facilitadores deveriam anotar e depois perguntar a um profissional de saúde. As dúvidas que não forem respondidas no dia do diálogo deverão ser esclarecidas no próximo diálogo comunitário.
- Se as pessoas começarem a falar de outro assunto, pedir ao grupo voltar ao tema em discussão, e propor que o outro assunto seja discutido num outro diálogo.

Exemplos de guião de discussão para diálogos sobre Bilharziose

- Instruções para o primeiro diálogo
- Exemplo de Diálogo - Tema: Conhecer a bilharziose
- Exemplo de Diálogo - Tema: Acção para evitar a bilharziose: como limitar contacto com água infectada?
- Exemplo de Diálogo -Tema: Campanha de distribuição de medicamentos

Instruções para o primeiro diálogo

Este projecto faz parte duma Pesquisa. É obrigatório obter o consentimento dos membros da comunidade em participar neste projecto.

Por isso, durante o primeiro diálogo, os facilitadores deveriam explicar o projecto durante a introdução ao diálogo e obter o consentimento dos participantes que eles concordam em participar neste projecto de pesquisa.

Mensagem chave a passar aos membros da comunidade:

- Os diálogos são implementados como parte de uma pesquisa
- Descrever brevemente os objectivos
- O facilitador irá informar os membros da comunidade que ele irá fornecer relatórios regulares no âmbito do esquema de monitoria.
- Um membro da direcção provincial da saúde poderá observar alguns diálogos comunitários.
- O facilitador devera pedir aos participantes e líderes comunitários de confirmar verbalmente o consentimento em participar.

A decisão da comunidade deve ser escrita na ficha de monitoria do primeiro diálogo.

Exemplo de discurso ao início do primeiro diálogo

Bom dia/boa tarde a todos. Meu nome é (seu nome), sou membro do Comité de Saúde desta comunidade. Sejam bem-vindos ao nosso encontro onde vamos discutir sobre a saúde da nossa comunidade.

A nossa comunidade foi escolhida para fazer parte dum projecto de pesquisa que pretende compreender como o uso de diálogos comunitários pode ajudar para a prevenção e controle de doenças tropicais negligenciadas, na província de Nampula. As doenças tropicais negligenciadas são um grupo de doenças que afectam as pessoas que vivem em zonas rurais de Moçambique e de outros países, tal como a Bilharziose.

Este projecto esta a ser conduzido pela Direcção Provincial de Saúde de Nampula em parceria com a Malaria Consortium. O projecto vai durar cerca de 20 meses e será implementado nos distritos de Eráti, Mecuburi, Murrupula e Mogovolas.

No âmbito deste projecto, eu recebi um treino recentemente e vou organizar, junto com os outros membros do nosso comité de saúde e da nossa comunidade, encontros regulares de forma de diálogos comunitários participativos.

Durante esses diálogos, vamos falar sobre a Bilharziose que é uma doença que ataca muitas pessoas na nossa comunidade. Durante o nosso diálogo ou conversa vamos ver alguns desenhos ou imagem sobre situações que acontecem e que tem a ver com a bilharziose. Cada um de nós vai falar sobre o que consegue ver, o que acha sobre a imagem ou cada um de nos vai responder a perguntas que posso colocar durante o nosso encontro. No final vamos decidir o que e como poderemos fazer a partir de hoje para prevenir e tratar esta doença.

Durante os nossos diálogos, eu vou preencher uma ficha de monitoria, anotando os temas que nos discutimos, o número de participantes nos nossos encontros, e as decisões tomadas e resultados na melhoria das práticas de saúde para combater a bilharziose. Também, um supervisor da direcção provincial ou distrital da saúde poderia visitar-nos e observar um diálogo comunitário. Adicionalmente, no âmbito desta pesquisa, alguns membros da nossa comunidade poderiam ser entrevistados pela Malaria Consortium no âmbito de obter as perspectivas dos próprios membros da comunidade sobre esta abordagem de diálogos comunitários ou sobre a bilharziose.

Todas informações serão guardadas sem anotar qualquer nome das pessoas. A informação só estará disponível para os pesquisadores que trabalham no estudo.

Participar no projecto é voluntário. Se você decidir não participar, não haverá consequências negativas. Para se recusar a participar, não precisa de dar nenhum motivo ou justificação.

A participação no estudo é uma oportunidade para nos podermos contar as nossas experiências, opiniões e ideias.

Se você tiver alguma dúvida, por favor pergunte-nos. (…)

Você também pode também entrar em contacto, se quiser, com nossos supervisores:

Malaria Consortium Nampula, Tel.: 84 389 7300

Direcção Provincial da Saúde Nampula, Tel.: 84 267 0917

Comité Nacional de Bioética para a saúde, Tel.: 824066350

Agora, queria pedir oralmente se todos nos aqui concordamos em participar? (…)
Obrigado/a, agora podemos começar.

Exemplo de Diálogo - Tema: Conhecer a bilharziose

Introdução do Diálogo Comunitário:

Cumprimentar aos participantes, por exemplo:

Bom dia/boa tarde a todos. Meu nome é___________, sou membro do Comité de Saúde desta comunidade. Sejam bem-vindos ao nosso encontro onde vamos discutir sobre a saúde da nossa comunidade.

Vamos falar sobre a Bilharziose que é uma doença que ataca muitas pessoas na nossa comunidade. Esta doença provoca saída de urina com sangue, dores de barriga, e se não for tratada a tempo pode levar a pessoa que tem esta doença a não ter a capacidade de ter filhos e até a morte.

Apresentar o tema, por exemplo:

Hoje vamos falar em conjunto, cada um de nós vai falar o que sabe ou o que ouviu falar sobre a bilharziose, o que é a bilharziose, quais são as causas, como se apanha, quais são os sintomas ou sinais de uma pessoa que tem esta doença, o que fazer para tratar e prevenir-se da bilharziose, o que as pessoas da nossa comunidade faziam antes, o que fazem hoje. Durante o nosso diálogo ou conversa vamos ver alguns desenhos ou imagem sobre situações que acontecem e que tem a ver com a bilharziose. Cada um de nós vai falar sobre o que consegue ver, o que acha sobre a imagem ou cada um de nos vai responder a perguntas que posso colocar durante o nosso encontro.

No final vamos decidir o que e como poderemos fazer a partir de hoje para prevenir e tratar esta doenca. Podemos começar?

Explorar o tema: Estimule um debate através de perguntas, como por exemplo:

- *Vocês já ouviram falar de bilharziose?*
- *O que é? Como se manifesta?*

Deixar vários participantes a partilhar os seus conhecimentos e experiências.

Pois, mostrar ou passar as imagens dos sinais e sintomas da bilharziose.

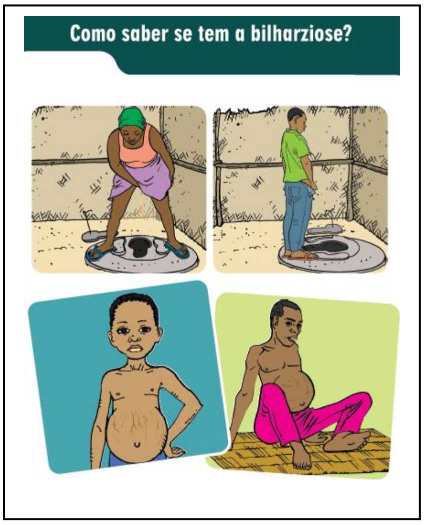

Perguntar:

- *O que se trata nestas imagens?*
- *Vocês já tiveram experiência pessoal desses problemas? Ou conhecem alguém que tenha tido esses problemas?*

Deixar vários participantes a partilhar as suas opiniões.

Depois, explicar que as imagens são os sinais mais comuns da doença bilharziose, usando as "Informações básicas sobre a bilharziose" (Como saber se tem a doença?) deste guião de diálogo comunitário.

A seguir, perguntar ao grupo:

- *Como se apanha a bilharziose?*

Deixar vários participantes a partilhar as suas opiniões.

Depois, mostrar ou passar as imagens dos comportamentos de risco:

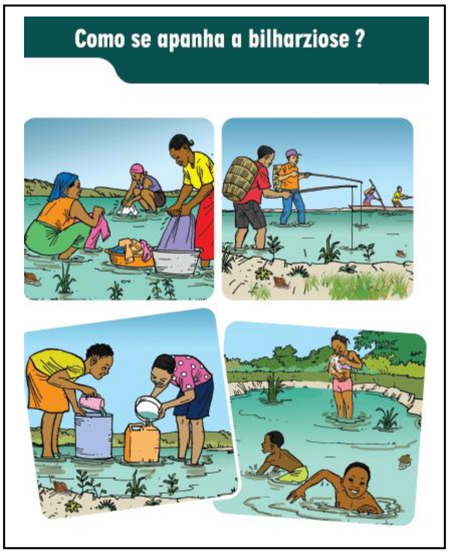

Perguntar:

- *O que estão a ver nestas imagens?*
- *É comum na vossa comunidade acontecer isto? Este comportamento é bom ou é de risco?*
- *O que fazem quando isto acontece na vossa comunidade?*

Deixar vários participantes partilhar as suas opiniões.

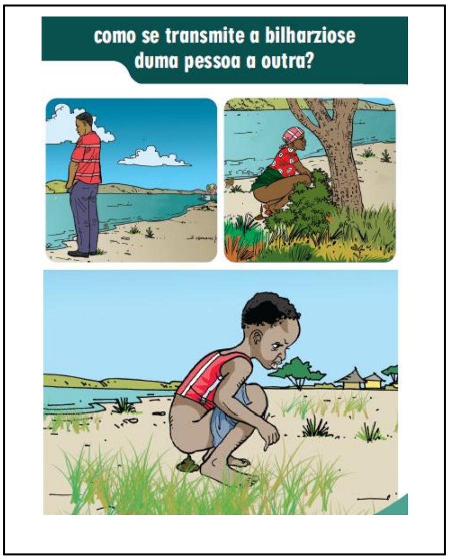

Depois, explicar cada imagem, e como as pessoas podem apanhar a doença através destes comportamentos de risco, usando as "Informações básicas sobre a bilharziose" (Como apanha a doença?) deste guião de diálogo comunitário.

se

Identificar Acções:

A seguir, perguntar ao grupo:

- *Quais pessoas afectadas pela Bilharziose?*
- *Como se trata a bilharziose?*
- *Quando vocês estavam doentes, o que faziam*?
- *Tinham sucesso com o tratamento e cura? Se não passou, por quê? Quais foram as dificuldades?*

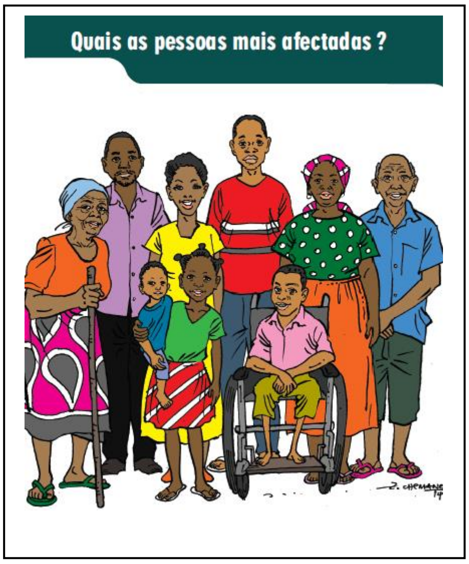

Deixar vários participantes partilhar as suas opiniões.

Depois, explicar como as pessoas com esta doença de bilharziose podem ser tratadas e curadas usando as "Informações básicas sobre a bilharziose" (Como se trata a bilharziose?) deste guião de diálogo comunitário.

Perguntar:

- *O que acham dessas ideais? Estes comportamentos são factíveis na nossa comunidade?*
- *Há alguma dificuldade para praticar estes comportamentos?*
- *Como ultrapassar essas dificuldades?*
- *Tem outras sugestões?*

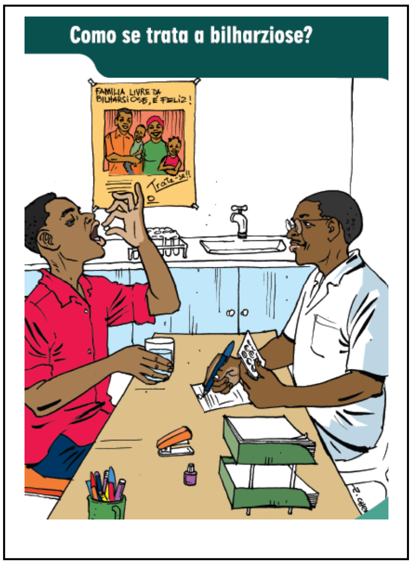

Pedir aos participantes que escolham uma ou duas acções concretas e factíveis que devem ser feitas em primeiro lugar.

Tomar decisões:

Perguntar aos participantes se concordam com os passos de acção para implementar as acções concretas que escolheram. Perguntar:

- *O que deve ser feito de concreto?*
- *Quem deve fazer isto? Como? Até quando?*

O facilitador pode sugerir acções ao grupo. Por exemplo: cada um dos participantes vai partilhar essas informações que foram discutidas com outros membros da família, e com amigos e vizinhos

Fazer um resumo:
O resumo vai depender do que foi discutido durante o diálogo. Depois, o facilitador deveria lembrar a todos as decisões tomadas pelo grupo durante a discussão.

Esclarecer as dúvidas: perguntar se os participantes têm perguntas; anotar as perguntas técnicas que não podem ser respondidas no diálogo, para ser abordadas no próximo encontro.

Agradecer aos participantes por terem partilhado as suas opiniões e marcar o próximo encontro.

Exemplo de Diálogo - Tema: Acção para evitar a bilharziose: como limitar contacto com água infectada?

Introdução do Diálogo Comunitário:

Cumprimentar aos participantes, por exemplo:

Bom dia. Bem-vindo aos que já participaram no nosso último diálogo, e bem-vindo aos que vão participar hoje pela primeira vez. Como sabemos, esta reunião não é uma aula; esta reunião é uma conversa para todos nós partilharmos os nossos conhecimentos e experiências, e discutirmos em conjunto o que a nossa comunidade poderia fazer para combater a bilharziose.

Apresentar o tema, por exemplo:

Hoje, vamos discutir mais em detalhes o que podemos fazer juntos para não ficarmos nas águas infectadas com bichos da bilharziose.

Fazer um resumo do que foi discutido anteriormente:

Antes de começar, queria pedir se há pessoas que já participaram nas nossas reuniões sobre bilharziose e que poderiam explicar aos outros o que é essa doença, como se previne e como se cura.

Pedir aos participantes a usarem as imagens para explicar o que eles aprenderam sobre a bilharziose. Corrigir ou completar as informações dadas, se for necessário, usando as "Informações básicas sobre a bilharziose" deste guião de diálogo comunitário.

Explorar o tema:

Estimular um debate através de perguntas, como por exemplo:

- *Já ouvimos que uma das maneiras de evitar a doença bilharziose é não ficar nas águas paradas. Agora, o que se deve fazer para não manter contacto com água infectada? Como?*
- *O que vocês têm feito nas vossas casas ou aqui na comunidade para evitar esse contacto?*
- *O que acham sobre o que tem feito, será que tem bons resultados? Quais foram os resultados?*

Deixar vários participantes a partilhar as suas sugestões e experiências. Pois, passar as imagens de comportamentos de risco.

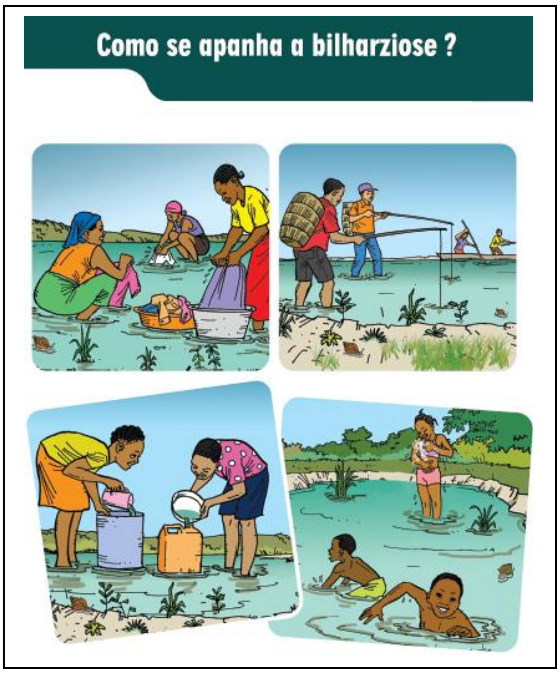

Perguntar:

- *O que estão a ver nesta imagem? Isto acontece na comunidade?*
- *Este comportamento é bom? Será que pode limitar o contacto com água contaminada?*
- *O que pode acontecer com estas pessoas?*

Deixar vários participantes partilhar as suas sugestões e experiências.

Identificar Acções:

Em seguida, explicar que há 3 acções para evitar a doença:

1. Não ficar na água parada durante muito tempo.
2. Também, deve se cuidar bem da água e da higiene individual e colectiva:
3. Participar nas campanhas do Ministério da Saúde que tratam dessa doença:

Perguntar aos participantes:

- *Quais são as maneiras mais seguras e factíveis na nossa comunidade de tirar água, fazer necessidades, pescar, lavar?*

Deixar os participantes fazer as suas sugestões. Depois, passar as imagens de comportamentos preventivos.

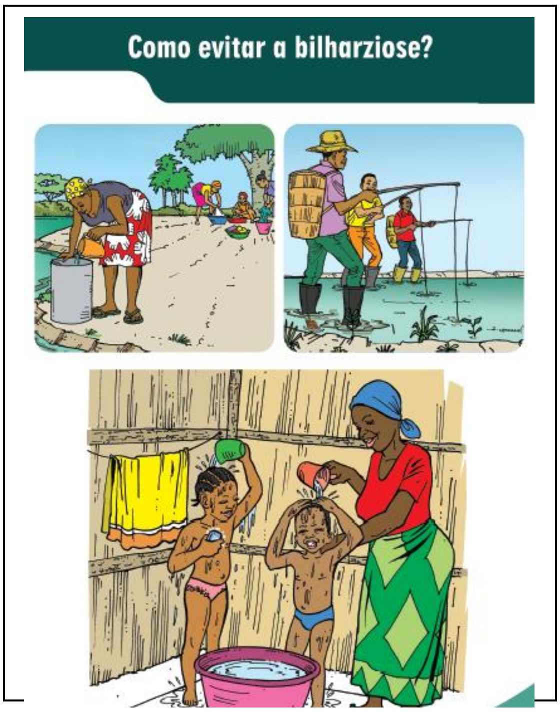

Fazer perguntas:

- *O que estão a ver nesta imagem?*
- *Isto acontece na comunidade? Este comportamento é bom? Será que pode ser eficaz para evitar a bilharziose?*
- *O que acham dessas ideais? Estes comportamentos são factíveis na nossa comunidade?*
- *Há alguma dificuldade para praticar estes comportamentos?*
- *Como ultrapassar essas dificuldades?*

- *Tem outras sugestões?*

Pedir aos participantes que escolham uma ou duas acções concretas e factíveis que devem ser feitas em primeiro lugar.

Tomar decisões:

Perguntar aos participantes se concordam com os passos de acção para implementar as acções concretas que escolheram. Perguntar:

- *O que deve ser feito de concreto?*
- *Quem deve fazer isto? Como? Até quando?*

O facilitador pode sugerir acções ao grupo. Por exemplo: cada um dos participantes vai partilhar essas informações que foram discutidas com outros membros da família, e com amigos e vizinhos

Fazer um resumo.
Por exemplo:
Hoje aprendemos juntos que existem alguns comportamentos que podem nos deixar doentes da bilharziose como por exemplo ficar com os pés na água dos charcos, no momento em que se lava roupa e pesca. Vimos que estas práticas são comuns aqui na nossa comunidade e decidimos que devemos tirar a água e lavar a roupa distante das águas do rio. Quando vamos ao rio pescar devemos usar botas ou parar nas margens do rio num lugar de terra firme e seco. Decidimos também que devemos falar com nossos familiares, amigos e vizinhos da comunidade para assumirmos os comportamentos de prevenção na nossa comunidade, participarmos nas campanhas de saúde para termos mais conhecimentos que podem ajudar-nos a melhorar a nossa saúde na comunidade, e ainda prometemos que cada um deve servir de exemplo na comunidade.

Esclarecer as dúvidas.

Por exemplo:
Alguém tem uma dúvida ou quer perguntar alguma coisa?

Agradecer e marcar o próximo encontro.

Por exemplo:
Chegamos ao fim do nosso diálogo hoje e o nosso próximo encontro será no próximo mês de Julho dia 20; se existir alguma alteração poderão saber de mim ou de outras pessoas que estão aqui que podem visitar-vos nas vossas casas. Muito obrigado a todos.

Exemplo de Diálogo -Tema: Campanha de distribuição de medicamentos

Introdução do Diálogo Comunitário:

Cumprimentar aos participantes, por exemplo:

Bom dia. Bem-vindo aos que já participaram no nosso último diálogo, e bem-vindo aos que vão participar hoje pela primeira vez. Como sabemos, esta reunião não é uma aula; esta reunião é uma conversa para todos nós partilharmos os nossos conhecimentos e experiências, e discutirmos em conjunto o que a nossa comunidade poderia fazer para combater a bilharziose.

Apresentar o tema, por exemplo:

Hoje vamos falar das campanhas de distribuição de medicamentos. Estas campanhas são feitas pelo Ministério da Saúde na nossa comunidade para tratar várias doenças incluindo a bilharziose e informar as pessoas como é que estas doenças são tratadas, o que fazer quando alguém está doente, onde é que devemos tratar. Estas campanhas são muito importantes para nós porque podem ajudar-nos a tratar pessoas que sofrem de bilharziose e outras doenças que atacam aqui na comunidade e também dar-nos muita informação.

Fazer um resumo do que foi discutido anteriormente, por exemplo:
Antes de começar, queria pedir se há pessoas que já participaram nas nossas reuniões sobre bilharziose e que poderiam explicar aos outros o que é essa doença, como se previne e como se cura.

Pedir aos participantes a usarem as imagens para explicar o que eles aprenderam sobre a bilharziose. Corrigir ou completar as informações dadas, se for necessário, usando as "Informações básicas sobre a bilharziose" deste guião de diálogo comunitário.

Explorar o tema:

Estimular um debate através de perguntas, como por exemplo:

- *Vocês já ouviram falar de campanhas de distribuição de medicamentos contra a bilharziose?*
- *O que é feito nas campanhas?*
- *Vocês já participaram em alguma campanha?*

Deixar as pessoas a darem as suas opiniões. Depois, passar as imagens de campanhas:

Fazer perguntas:

- *O que está acontecendo nestas imagens?*
- *Já viram algo como estes (mostrar imagens de campanhas) acontecendo?*
- *O que acharam das campanhas? Foram boas? O que não funcionou bem?*
- *Aqui na comunidade acontecem campanhas?*
- *O que se fala nas campanhas?*
- *Quais medicamentos são dados durante essas campanhas?*
- *Quem deve tomar o medicamento?*
- *Porque se deve tomar o medicamento mesmo sem sentir-se doente?*

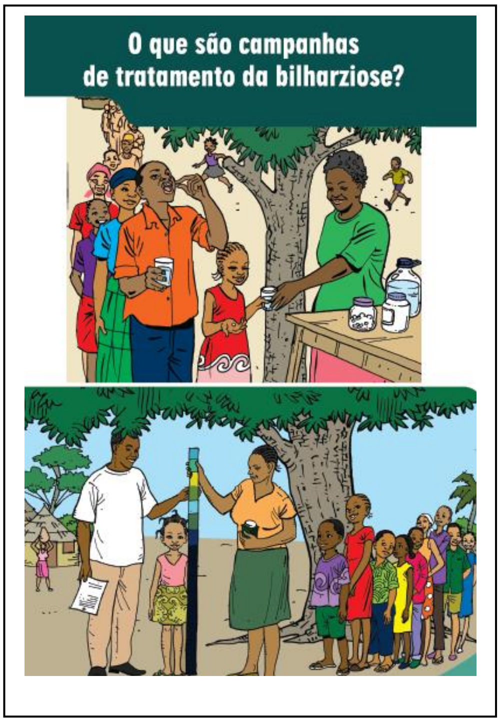

Depois, explicar cada imagem, usando as "Informações básicas sobre campanhas de tratamento da bilharziose" deste guião de diálogo comunitário.

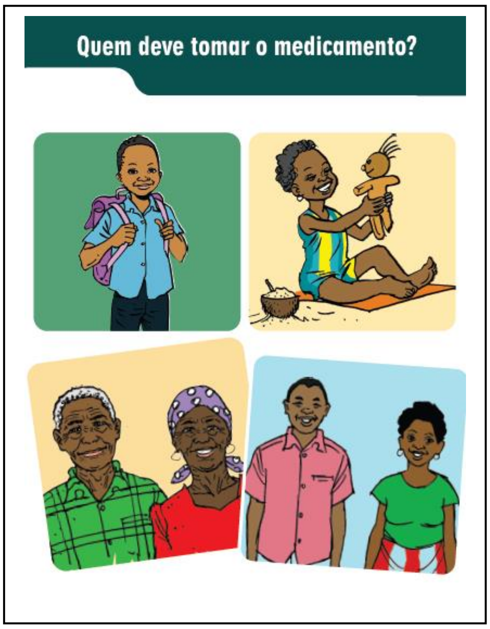

Identificar Acções:

Em seguida, estimular um debate:

- *Quando isto acontece as pessoas aqui na comunidade participam? Como?*
- *Como a comunidade poderia ajudar na campanha?*
- *O que deve ser feito para que mais pessoas participem nas campanhas de distribuição de medicamentos?*

Depois, explicar Como a comunidade poderia ajudar na campanha, usando as "Informações básicas sobre campanhas de tratamento da bilharziose" deste guião de diálogo comunitário.

Por exemplo:
Assegurar que todos os membros da família tomam o medicamento;

Encorajar vizinhos e amigos a participar também;

Ajudar os líderes comunitários e membros do comité de saúde na disseminação da informação sobre o local, a data e hora da distribuição de medicamentos;

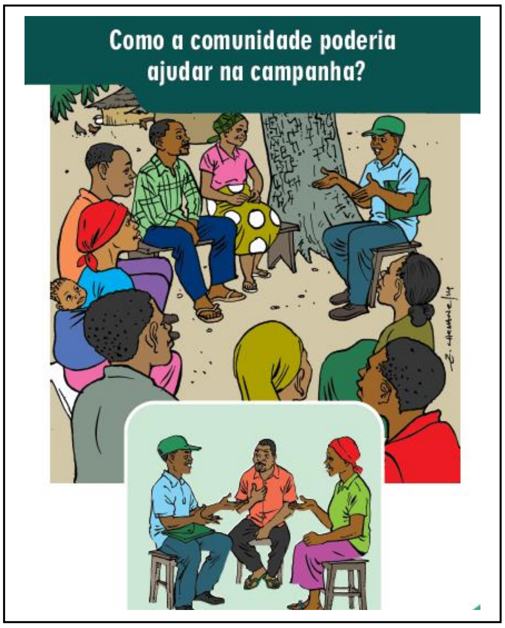

Pedir aos participantes que escolham uma ou duas acções concretas e factíveis que devem ser feitas em primeiro lugar.

Tomar decisões:

Perguntar aos participantes se concordam com os passos de acção para implementar as acções concretas que escolheram. Perguntar:

- *O que deve ser feito de concreto?*
- *Quem deve fazer isto? Como? Até quando?*

O facilitador pode sugerir acções ao grupo. Por exemplo: cada um dos participantes vai partilhar essas informações que foram discutidas com outros membros da família, e com amigos e vizinhos

Fazer um resumo. Por exemplo:
Depois deste diálogo que tivemos, sabemos na nossa comunidade algumas pessoas participaram nas campanhas outras não participaram porque não sabiam. Ouvimos que devemos fazer sensibilização na nossa família, grupo de amigos e vizinhos para mais pessoas participarem nas campanhas. Através dessas campanhas podemos ter mais conhecimentos sobre como nos prevenirmos das doenças, incluindo a bilharziose. Por meio dessas campanhas podemos tomar medicamentos que matam os bichos dentro do corpo e deixam-nos mais saudáveis.

Esclarecer as dúvidas.

Por exemplo: *Existem perguntas para fazer?*

Agradecer e marcar o próximo encontro. Por exemplo:

Muito obrigado. Terminamos o nosso diálogo hoje e voltaremos a nos ver no dia 25 de Agosto. Lembrem que decidimos que vamos sensibilizar nossos vizinhos e família para participarem nas campanhas. No próximo encontro, vamos ouvir primeiro a experiência de cada um de nós na sensibilização comunitária para mais parentes (familiares), amigos e vizinhos participarem nas campanhas. Muito obrigado!

Ficha de Monitoria<br>Diálogo Comunitário	Numero

**Nome dos Facilitadores**	
**Distrito**	
**Posto Administrativo**	
**Localidade**	
**Comunidade**	

Local do diálogo: ....

Data do diálogo: ....

Hora de Inicio …………………………………….. Hora de fim ……………………………………………

Número de participantes: ....

Homens: .... Mulheres: ....

Explorar o tema: Quais foram os temas para o diálogo?

....

....

....

Observações:

---

1) Sobre logística

....

....

....

2) Sobre o guião de diálogo ou com o álbum seriado

....

....

....

3) Sobre perguntas técnicas (escreve aqui todas as questões que você ou os membros da comunidade não tem certeza ou não sabem a resposta correta)

....

....

....

4) Outras observações ou sugestões

....

....

....

Próximo diálogo:

Quando será? ....

Qual será o tema? ....

Ficha de planificação e seguimento dos diálogos

**Numero**	**Data**	**Decisões tomadas**	**Responsabilidades: Quem é que vai fazer, e quando?**	**Ponto de situação ou Resultados**	**Desafios na implementação das decisões**
