# Supplementary material for: Evaluating the feasibility and acceptability of a community dialogue intervention in the prevention and control of schistosomiasis in Nampula province, Mozambique
Source: PLoS One. 2021 Aug 5;16(8):e0255647. doi: 10.1371/journal.pone.0255647 (PMC8341517; doi:10.1371/journal.pone.0255647)
Supplement: Attachment — Submitted filename: 2020-06-30 Response to Reviewers.docx [file pone.0255647.s005.docx]

**Engaging affected communities in the prevention and control of schistosomiasis: evaluating the feasibility and acceptability of a Community Dialogue intervention in Nampula province, Mozambique**

Sandrine Martin, Christian Rassi, Valdimar Antonio, Kirstie Graham, Jordana Leitão, Rebecca King, Ercilio Jive

**Manuscript submitted for review to PLOS ONE (PONE-D-19-34788)**

**Authors’ response to editor and reviewers’ comments**

Dear Mr. Enrique Castro-Sánchez,

We thank you and the reviewers very much for a thorough reading and constructive and helpful criticism of our manuscript and for the opportunity to revise and resubmit.

Thank you for the comments of the reviewers and the editors. We found the comments very constructive, and sincerely appreciate the time and attention dedicated to this work.

We are pleased to submit the improved research article for your consideration.

Below, we have addressed each of these comments and give a point-by-point response. We have numbered the reviewers’ comments and respond using bold font. Note that line numbers in our responses refer to the revised manuscript without tracked changes.

Journal Requirements

Query 1: Please ensure that your manuscript meets PLOS ONE's style requirements, including those for file naming.

**Thank you for pointing this out. Accordingly, we have reviewed the guidelines and adjusted the manuscript’s style to the journal’s requirements, with special attention to file naming, formatting and citing of figures, tables and supplementary files.**

Query 2: Please include the registration number for the clinical trial referenced in the manuscript.

**No clinical trial was referenced in the paper. The word ‘trialled’ used at Line 279 refers to ‘other CD models previously trialled and published in Mozambique’, with reference to two studies, none of which were clinical trials. As stated under the Ethics section of the manuscript, for this study, ‘Ethical approval for the study was granted by the University of Leeds School of Medicine Research Ethics Committee (SoMREC/13/071) and the Comité Nacional de Bioética para Saúde in Mozambique (42/CNBS/2014)’.**

Query 3: We note that you have indicated that data from this study are available upon request.

**Indeed, as per the consent forms used in this study and approved by the ethics committees referenced in the article, the use of individual participant data for future research purposes is conditional on ethical approval for additional research questions. Data access requests will be reviewed by Malaria Consortium’s Research Group, which can be contacted at** [**research.lead@malariaconsortium.org**](mailto:research.lead@malariaconsortium.org)**. Interested researchers do not need membership with Malaria Consortium’s Research Group to gain access to data. The Research Group will review if ethical approval is required and if it has been obtained before making the data available to the researcher.**

**Equivalent statements have been included in two previous articles relating to this study and published in PLOS journals:**

**Rassi C, Kajungu D, Martin S, Arroz J, Tallant J, Zegers de Beyl C et al. Have you heard of schistosomiasis? Knowledge, attitudes and practices in Nampula province, Mozambique. PLoS Neglected Tropical Diseases. 2016;10(3): e0004504.**

**Rassi C, Martin S, Graham K, de Cola MA, Christiansen-Jucht C, Smith LE et al. Knowledge, attitudes and practices with regard to schistosomiasis prevention and control: Two cross-sectional household surveys before and after a Community Dialogue intervention in Nampula province, Mozambique. PLoS Negl Trop Dis. 2019;13(2): e0007138.**

Query 4: We note that [Figure 2] in your submission contain map images which may be copyrighted.

**A new map has been designed for this publication, and consent to publish has been obtained from the designer (see uploaded file: 2020-09 Content Permission Form Fig 2.pdf)**

Reviewer 1

1.1. Comment asking to check the consistent use of acronyms and definition of acronyms.

**This was corrected, specifically on line 94 WHO acronym and line 275 (CDF acronym).**

1.2. Comment recommending to re-consider the value of including already-published findings from the pre-versus post household surveys.

**Thank you for the suggestion. However, as per Reviewer 2’s request for clarity and focus, supported by the Editor, we have re-focused the paper on the feasibility and acceptability of the CDA. Consequently, we decided not to elaborate further on previously published findings relating the outcomes of the CDA on knowledge, attitudes and practices.**

Reviewer 2

2.1. Comment requesting to re-consider the paper’s focus and formulate firmer and clearer aims and objectives.

**We agree with the reviewer’s assessment. Accordingly, we have reviewed the scope of the paper to focus on feasibility and acceptability of the CDA in the context of NTD programming. Accordingly, throughout the manuscript, we made the necessary edits where needed to ensure that the respective aims and objectives of the CDA, the pilot study, and this manuscript are clearly spelt out as follow:**

**The CDA, in the context of schistosomiasis, aims at improving communities’ uptake of recommended prevention and treatment measures, such as MDA adherence, seeking help from qualified health care providers and adopting basic hygiene and sanitation protective practices (intervention description Lines 161 to 164)**

**The study** **aimed to test the CDA in the context of schistosomiasis prevention and control, and to evaluate its feasibility, acceptability and potential to improve communities’ level of knowledge, attitudes and practices, with the overall aim of improving schistosomiasis prevention and control (Introduction, lines139 to 142).**

**This paper presents a detailed description of the CD intervention, and summarise findings with regard to its feasibility and acceptability, drawing on the qualitative and process evaluation data collected throughout the study (Introduction, lines 147 to 149).**

**The main findings summarized in the conclusion (lines 1270 to 1277) have been re-aligned to speak to the feasibility and acceptability of the intervention.**

**The revised focus on feasibility and acceptability is reflected in substantial modifications made in the Results and Discussion sections, to ensure that the data presented supports the key findings.**

2.2. Comment asking for definitions of the concepts of 'feasibility' and 'acceptability'.

**Definitions have been included in this revised copy in the Methods section, Study design, lines 325 to 338. Feasibility is conceived and assessed along the five dimensions recommended by UK Medical Research Council’s guidance: 1) fidelity, 2) reach, 3)dose delivered, 4) adaptation and 5) mechanisms of impact. We define acceptability as per Peters et al. (2008) which in the context of health interventions, refers to the degree of responsiveness of the intervention to the social and cultural expectations of individuals and communities.**

2.3. Comment asking to review the salient literature on community participation and the use of educational strategies in the public health sector both globally and in Mozambique.

**Thank you for this suggestion. The salient literature on community participation in health programmes was reviewed, including past and recent systematic literature and umbrella reviews (Questa et al. 2020; Draper et al. 2010; Farnsworth et al. 2014; Rosato et al. 2008; Rifkin et al. 2014; Rifkin et al. 2000), and referenced in the paper (Introduction, lines 117to 122).**

2.4. Comment requesting for a nuanced conceptualisation of community participation and situating the use of the CDA within this concept.

**We fully agree on the importance of defining and measuring community participation. We wish to clarify that it was beyond the scope of this study to increase community participation in NTD programme. The purpose of testing the CDA was to improve the efficiency and reach of health promotion efforts of the NTD programme. Our assumption was that the use of participatory communication techniques could improve knowledge, attitudes and practices, and contribute to improving communities’ uptake of recommended prevention and treatment measures (as stated in the intervention description section).**

**Considering that the CDA could provide a platform for the NTD programme to engage with communities, we agree with the need to situate the CDA within the community participation continuum, and have done so in the Discussion section of the paper at lines 1085 to 1092. We situated the CDA using the modified scale proposed by Draper et al. ranging from mobilisation to collaboration and empowerment. The CDA can be located at the lower level of participation, described as community mobilisation: it involves enabling selected community members to conduct participatory CDs. However, topics and tools were predetermined outside of the community.**

**We agree that it would have been interesting to explore this aspect in more depth. We attempted to apply Draper et al.’s framework for the assessment of community participation. However, the depth of the data collected (see limitations mentioned at lines 1231 to 1233) was not sufficient to conduct a meaningful analysis of the five dimensions of community participation (needs assessment, leadership, management, organisation and resource mobilisation). Consequently, we decided not to include this dimension in the paper.**

2.5. Comments asking for revision of some of the language included in the paper.

**Thank you for pointing this out. This was addressed as outlined below.**

- ‘The authors refer to a "fragile country". This is an ideologically loaded term’.

**This problematic wording has been removed as follows:**

- ‘The authors use the term "developing country" which also carries problematic meanings’.

**At line 120, the term ’developing country’ was used, it has been replaced by ‘low and middle income countries (LMIC) in this revised copy.**

2.6. Comments pointing out unaddressed limitations in the methodology of the study.
- Reviewer: ‘In the methodology section it would be instructive to review briefly the difficulties and limitations of focus group discussions especially as they relate to the question of participant response set and the dominance of vocal voices’.

**We have added this aspect as an additional limitation of the study at lines 1247 to 1248.**

- Reviewer: ‘On page 19 the authors state: "discussion guides were not translated into Macua, as there is no tradition of reading or writing in this language...." In the absence of contextual details and information on modes of knowledge making and transmission in the specific cultural context, such a claim may be interpreted as problematic’.

**Contextual information has been added at lines 415 to 416. In Mozambique, Portuguese was adopted after colonization as the official language and is the general language of education; only in 2018 local languages were introduced in primary schools . Consequently, adults who are literate are more comfortable reading and writing in Portuguese and often do not read and write easily in their local language.**

- Reviewer: ‘Lastly, did the researchers do any back translations of the transcripts from English to Macua to ensure optimal accuracy of the translation of texts?’

**As stated at lines 420 to 426, all FGDs were conducted in local language, audio-recorded and subsequently transcribed verbatim, including non-verbal clues, into Portuguese from the audio recording. All the data set, including transcripts, was produced in Portuguese, and not translated into English.** **Only quotes originally in Portuguese in the transcripts have been translated into English for this publication. These quotes were not back translated into Macua. A sentence was added in the limitations section lines 1235 to 1237 of the manuscript to reflect this potential weakness.**

We hope these revisions meet the editor and reviewers’ expectations. On behalf of my co-authors, I thank you for your consideration of this resubmission. We appreciate your time and look forward to your response.

Sincerely yours,

Sandrine Martin
