# Supplementary material for: Evaluating the feasibility and acceptability of a community dialogue intervention in the prevention and control of schistosomiasis in Nampula province, Mozambique
Source: PLoS One. 2021 Aug 5;16(8):e0255647. doi: 10.1371/journal.pone.0255647 (PMC8341517; doi:10.1371/journal.pone.0255647)
Supplement: Attachment — Submitted filename: PLOS ONE Review.docx [file pone.0255647.s006.docx]

1. This is a very good and important study. However, I have a few concerns that I think if addressed would improve the quality of the paper.
2. The title of this article should be changed, it’s too long, it should read.

- Evaluating the feasibility and acceptability of a Community Dialogue intervention in the prevention and control of schistosomiasis in Nampula province, Mozambique.

3. In the abstract

- Check and revise the tense.
- In the results, I would expect a sentence or two on the mechanisms of how the dialogue increased knowledge, demand and utilization.

4. I notice the authors stating feasibility, acceptability and potential to…. in the objectives. I think the main objective should be edited in the abstract, author summary and the introduction. It should state as follows;

- To evaluate the acceptability and feasibility of using the CDA to improve communities’ level of knowledge, attitudes and practices, and participation in wider schistosomiasis control efforts.

5. Methods

- I think the two data analysis sections should differentiated. One can state ‘Qualitative data analysis’ and the Other one can read ‘Analysis of routine monitoring and evaluation data’

6. I think there should be section where you explain the implementation outcomes of interest to this study. But most importantly it is how conceptualize feasibility and acceptability in this study. That should be clearly explained. I think you will benefit from reading a paper by Deborah J, Bowen, called How we design feasibility studies. Also, you need to have a clear relook at Outcomes of implementation research by proctor. I think these papers should help you decide whether to stick with evaluating feasibility and acceptability, or evaluating the implementation process of the community dialogue approach in your context.
7. Results

- I would suggest that the authors use some numbering system for the various themes for clarity in the presentation of the themes
- The presentation of results could be improved. Firstly, I would want to see a coding structure or a table depicting the thematic areas and how they relate to each before starting to describe the results. Here is my suggestion of how this table would look like given the results and theme the authors present. However, I would suggest that the authors don’t just replicate this but reflect on my comment below with regards to overlapping themes and repeated narrations. So, they would have to decide what themes to merge or not.

| Main theme | Sub-theme |
| --- | --- |
| 1. Fidelity | Sensitization |
|  | Volunteer selection |
|  | CDF training |
| 2. Adaptation of the CD intervention | CDF catchment area (could be reach depending on how you narrate) |
|  | Topics discussed |
|  | Support to the CDF |
| 3. Reach CD intervention | CDs conducted |
|  | CDs attendance |
|  | CDF recruited |
|  | Communities reached |
| 4. Acceptability CD intervention | Your narration should be split in sub-themes |
| 5. Mechanisms of CD intervention impact | Ten-step methodology (to what?) |
|  | Exploration (of what) |
|  | Identifying action (for what) |
|  | Decision making (on what?) |
|  | Participatory discussion |
| 6. CD intervention outcomes | Knowledge and misconception of schisto |
|  | Hygiene and sanitation practices |

- Line 549 should be ‘appeared’
- Some of the thematic areas are lacking quotes like CD attendance, support to CDF, CD attendance should have a quote because there is data from FGDs being described. For instance, a quote on CD attendance should be one describe what benefits people derived from attending the CD.
- Line 729 says exploration, exploration of what? A them must be self-explanatory. And you do this consistently in the results section. Please edit that. Ensure the thematic heading is clear to someone with no knowledge of your project.
- I notice some overlaps and repetitions in the description of the themes, and it happens throughout the result section. For example, you talk about knowledge under acceptability as well as under outcomes. You need to package your presentation in such a way that each narration is one place only and not repeated. Some themes are very much similar, I would advise that you merge them.
- Line 636 states adaptations, adaptations of what?
- Line 934 says outcomes? What kind of outcomes are these? That should be qualified

8. Discussion

- This section will require substantial modification to align with the reorganization of the results.
