# Supplementary material for: Evaluating the feasibility and acceptability of a community dialogue intervention in the prevention and control of schistosomiasis in Nampula province, Mozambique
Source: PLoS One. 2021 Aug 5;16(8):e0255647. doi: 10.1371/journal.pone.0255647 (PMC8341517; doi:10.1371/journal.pone.0255647)
Supplement: Attachment — Submitted filename: 2021-04-25 Response to Reviewers.docx [file pone.0255647.s007.docx]

**Evaluating the feasibility and acceptability of a Community Dialogue intervention in the prevention and control of schistosomiasis in Nampula province, Mozambique**

Sandrine Martin, Christian Rassi, Valdimar Antonio, Kirstie Graham, Jordana Leitão, Rebecca King, Ercilio Jive

**Manuscript submitted for review to PLOS ONE (PONE-D-19-34788)**

**Authors’ response to reviewers’ comments**

Dear Mr. David Joseph Diemert,

We thank you and the reviewer very much for a thorough reading and constructive and helpful criticism of our manuscript and for the opportunity to revise and resubmit.

We found the comments very constructive, and sincerely appreciate the time and attention dedicated to this work.

We are pleased to submit the improved research article for your consideration.

Below, we have addressed each of these comments and give a point-by-point response. We have numbered the reviewers’ comments and respond using bold font. Note that line numbers in our responses refer to the revised manuscript without tracked changes.

Reviewer 3

1 Title is too long.

**The title was edited and shortened.**

2 Abstract: Check tense and insert a sentence or two on mechanisms of impact.

**The tense was harmonized throughout and a sentence on mechanism of impact was inserted in both the abstract and the author summary.**

3 Methods: differentiate two data analysis sections; conceptualize feasibility and acceptability and decide whether to stick with focus on feasibility and acceptability or evaluating implementation process.

**In the Methods section, the data analysis sub-headings were edited to specific data analysis of monitoring data and data analysis of qualitative data.**

**We agree with the reviewer’s advice for clarity and focus of the paper and overall conceptualization of the study. Accordingly, we have revised the scope of the paper to focus on feasibility and acceptability of the CDA in the context of NTD programming.**

**Throughout the abstract (lines 29-32), author summary (lines 60-64) and introduction (lines 145-148) sections of the manuscript, we made the necessary edits to ensure that the aims and objectives of study are consistently spelt out.**

**Definitions of the concepts of 'feasibility' and 'acceptability' have been included in this revised copy in the Methods section, Study design, lines 338 to 353. Feasibility is conceived and assessed along the five dimensions recommended by UK Medical Research Council’s guidance: 1) fidelity, 2) reach, 3) dose delivered, 4) adaptation and 5) mechanisms of impact. We define acceptability as per Peters et al. (2008) which in the context of health interventions, refers to the degree of responsiveness of the intervention to the social and cultural expectations of individuals and communities.**

4 Results:
  4.1 Improve the presentation and organisation of results for clarity of the themes and sub-themes

**As per reviewer’s suggestion, a table has been inserted at the beginning of the results section which summarizes the main themes and sub-themes under each component (feasibility and acceptability) of the evaluation; a numbering system of the main themes and sub-themes was also adopted as per reviewer’s suggestion, to help the reader navigate the findings. We hope this has brought clarity.**

4.2 Ensure themes and sub-themes headings are self-explanatory

**In line with the organization of main themes and sub-themes, respective headings and sub-headings were inserted were missing and edited where needed to ensure they can be understood by reader without prior knowledge of the intervention.**

4.3 Some thematic areas lacked quotes

**Relevant quotes were added to the sub-themes of ‘CD attendance’ (lines 675-679) and ‘support to CDFs’ (lines 752-755) which draw on FGD data.**

4.4 Reduce repetitions and overlaps across themes

**With the re-focusing of the paper on the feasibility and acceptability dimensions of the intervention’s evaluation, and the re-organization of themes and sub-themes under these two components, repetitions and overlap have been addressed.**

5 Discussion requires substantial modifications to align with the reorganization of the results.

**The revised focus on feasibility and acceptability is reflected in substantial modifications made in the Discussion section, to ensure that the data presented supports the key findings. The main findings summarized in the conclusion (lines 1317 to 1327) have also been re-aligned to speak to the feasibility and acceptability of the intervention.**

We hope these revisions meet the editor and reviewers’ expectations. On behalf of my co-authors, I thank you for your consideration of this resubmission. We appreciate your time and look forward to your response.

Sincerely yours,

Sandrine Martin
